# Studying Genome Heterogeneity within the Arbuscular Mycorrhizal Fungal Cytoplasm

**DOI:** 10.1093/gbe/evv002

**Published:** 2015-01-07

**Authors:** Eva Boon, Sébastien Halary, Eric Bapteste, Mohamed Hijri

**Affiliations:** ^1^Département de Sciences Biologiques, Institut de Recherche en Biologie Végétale, Université de Montréal, Quebec, Canada; ^2^CNRS, UMR7138, Institut de Biologie Paris-Seine, Paris, France; ^3^Sorbonne Universités, UPMC Univ Paris 06, Institut de Biologie Paris-Seine (IBPS), Paris, France

**Keywords:** genome evolution, network analysis, genome heterogeneity, arbuscular mycorrhizal fungi, symbiosis, next generation sequencing

## Abstract

Although heterokaryons have been reported in nature, multicellular organisms are generally assumed genetically homogeneous. Here, we investigate the case of arbuscular mycorrhizal fungi (AMF) that form symbiosis with plant roots. The growth advantages they confer to their hosts are of great potential benefit to sustainable agricultural practices. However, measuring genetic diversity for these coenocytes is a major challenge: Within the same cytoplasm, AMF contain thousands of nuclei and show extremely high levels of genetic variation for some loci. The extent and physical location of polymorphism within and between AMF genomes is unclear. We used two complementary strategies to estimate genetic diversity in AMF, investigating polymorphism both on a genome scale and in putative single copy loci. First, we used data from whole-genome pyrosequencing of four AMF isolates to describe genetic diversity, based on a conservative network-based clustering approach. AMF isolates showed marked differences in genome-wide diversity patterns in comparison to a panel of control fungal genomes. This clustering approach further allowed us to provide conservative estimates of *Rhizophagus* spp. genomes sizes. Second, we designed new putative single copy genomic markers, which we investigated by massive parallel amplicon sequencing for two *Rhizophagus irregularis* and one *Rhizophagus* sp. isolates. Most loci showed high polymorphism, with up to 103 alleles per marker. This polymorphism could be distributed within or between nuclei. However, we argue that the *Rhizophagus* isolates under study might be heterokaryotic, at least for the putative single copy markers we studied. Considering that genetic information is the main resource for identification of AMF, we suggest that special attention is warranted for the study of these ecologically important organisms.

## Introduction

The multicellular individual is a functionally integrated assemblage of cells that share the same evolutionary fate, and can also be referred to as an organism. Even though there is no consensus on how to define “the individual,” many definitions depend on genome homogeneity, that is, all cells in an individual are expected to contain the same nuclear genome (Santelices 1999). The popularity of this criterion is based on the assumption that intraorganismal genetic heterogeneity (IGH) leads to conflict within the organism and thus stands in the way of its survival. IGH can indeed be detrimental to the multicellular organism (Biesecker and Spinner 2013). However, recent reviews on IGH in nonmodel systems question the ubiquity of the genetically homogeneous organism and multiple occurrences of heterokaryosis have been reported (Santelices 1999; Pineda-Krch and Lehtila 2004; Pepper and Herron 2008; Folse and Roughgarden 2010).

A group of organisms that undoubtedly evokes questions about the defining criteria of individuality are the root-inhabiting arbuscular mycorrhizal fungi (AMF), which form their own phylum, the Glomeromycota (Schussler et al. 2001). AMF improve nutrient uptake in their host plants, and buffer the plant against abiotic and biotic stresses (Van Der Heijden and Sanders 2002). These fungi significantly increase plant growth rates, although benefits vary depending on the composition of the AMF and plant community (Van der Heijden et al. 1998). AMF are of great potential interest to agriculture, yet advances in understanding the genetics and biology of these organisms have been slow (Sanders and Croll 2010).

As obligate symbionts with a long generation time, AMF are challenging study organisms. Limited data are available for only a subset of taxa for which axenic cultures or pot cultures have been established from single spores (so-called “isolates”) (Tisserant et al. 2012, 2013).

A major criticism against the possibility of heterokaryosis in AMF is that polymorphism could also be structured within nuclei, as duplicated genes (Hosny et al. 1999; Rosendahl and Stukenbrock 2004). A population genetic study of the highly polymorphic *PLS* gene has suggested that the observed genetic diversity (13 *PLS* alleles) occurred within each nucleus (Pawlowska and Taylor 2004). However, this evidence was debated: The copy number of the *PLS* marker used to demonstrate homokaryosis in AMF was found to be lower than its intraisolate allelic diversity (Hijri and Sanders 2005), and other hypotheses fit the data equally well (Bever and Wang 2005). The evidence against heterokaryosis brought forward by Stukenbrock and Rosendahl (2005a) is based on single-strand conformation polymorphism and does not offer the resolution to distinguish between nucleotide differences within variants. Furthermore, for one of the markers used in this study, the Large Subunit rDNA, more alleles were recovered within the same isolate than the estimated gene copies per nucleus (Boon et al. 2010), indicating genetic differentiation between genomes for at least this locus. Recent publications of the *Rhizophagus irregularis* genome (Tisserant et al. 2013) and single nucleus sequencing (Lin et al. 2014) report evidence in favor of homokaryosis, but it is unclear whether the approach adopted in these studies is sufficient to provide a definite answer to the debate.

In contrast, several recent studies are in support of the heterokaryosis hypothesis. First, there is evidence for within-isolate sequence polymorphism in *R. irregularis* DAOM 197198 (synonym *Glomus irregulare*) and *Glomus etunicatum* (synonym *Claroideoglomus etunicatum*) transcripts (Boon et al. 2010; Tisserant et al. 2012). Second, the possibility of segregation of genetic variation between parent and offspring has been demonstrated for *R. irregularis* (Angelard et al. 2010) and *G. etunicatum* (Boon et al. 2013). Patterns of genetic segregation between parent and clonal offspring indicate that different fractions of genetic variation are passed on to different spores. Moreover, this variation appears to make a difference to the phenotype of the offspring isolate (Angelard and Sanders 2011). Third, within-isolate heterokaryosis has been demonstrated for several loci (review in Boon et al. 2010). Fourth, several AMF taxa seem at no part of their life cycle reduced to a single nucleus (Jany and Pawlowska 2010; Marleau et al. 2011; Ehinger et al. 2012). This latter observation offers a proximate, mechanistic explanation for high levels of genetic polymorphism in AMF isolates. This peculiar genomic organization might be the result of the absence of a bottleneck of genetic variation at any point in the AMF life cycle, which sets AMF apart from filamentous fungi, which are heterokaryotic only in a part of their reproductive cycle.

High levels of genetic variation within the AMF cytoplasm lead to conceptual as well as practical challenges to studying the real extent of IGH in AMF. An expanded array of methods is required to study genome structure and organization of AMF genetic diversity. We propose that relatively cost-effective and easily applicable methods inspired by metagenomics can be used within the cytoplasm of AMF isolates to provide estimates of genetic diversity in an organism with potentially genetically differentiated genomes. We adopted two complementary approaches to study the organization of genome diversity in AMF, focusing on polymorphism both at a genome-wide scale and in single copy loci.

**Fig. 1.—:**
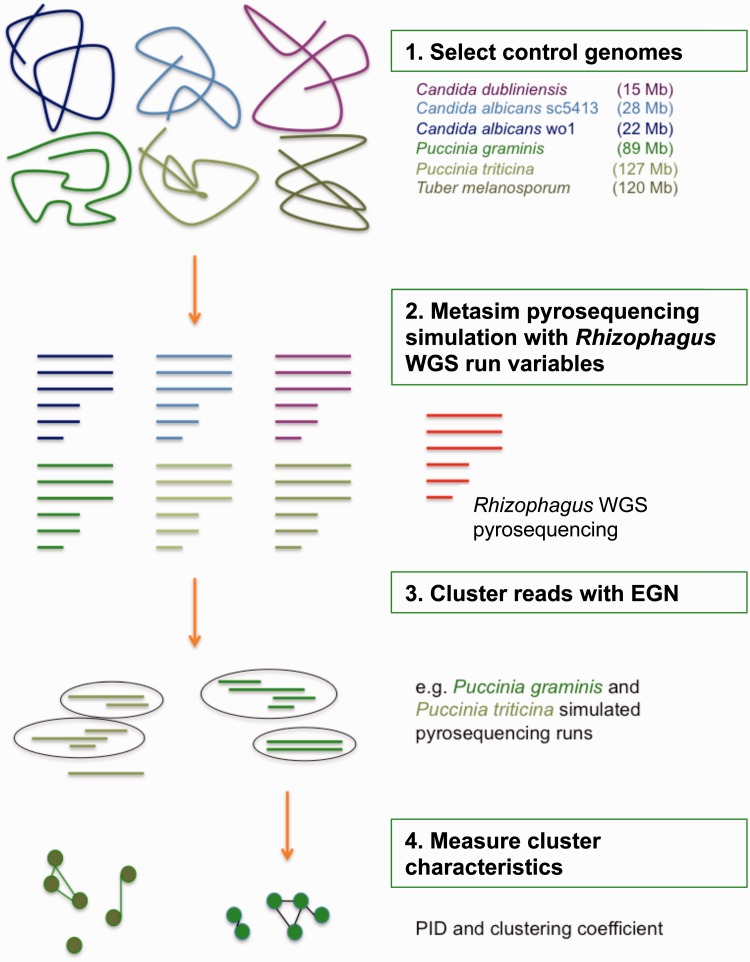
A graphic representation of the evolutionary network workflow.

**Fig. 2.—:**
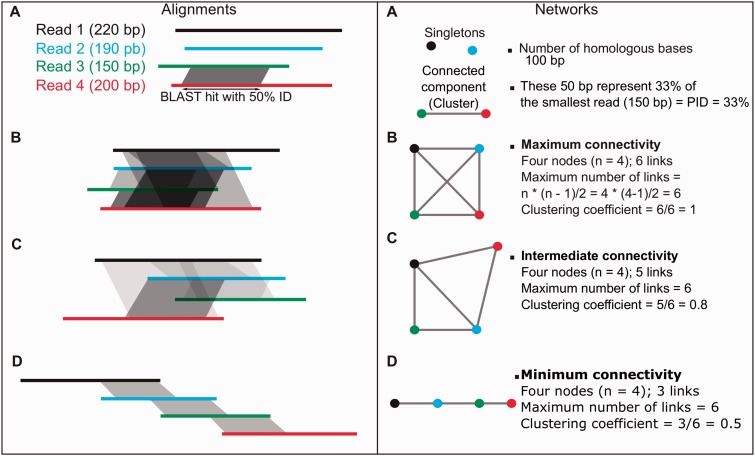
Description of the two variables employed to describe similarity clusters. Sequence alignments and their corresponding networks are shown. (*A*) The PID is the percentage of identical positions on the shortest sequence of an aligned reads pair. The PID per cluster is the average PID for all aligned reads pairs. (*B*)–(*D*) represent three scenarios of maximum, intermediate, and minimum connectivity, respectively. The clustering coefficient is the number of aligned reads pairs in the cluster, divided by the maximum number of possible pairs.

First, we estimated the genome-wide distribution of sequence differentiation. For this, we used a method that clusters data from whole-genome shotgun pyrosequencing runs of two *R. irregularis* isolates and one *Rhizophagus* sp*.* isolate, together also referred to as *Rhizophagus*. Clustering of reads was performed using sequence similarity networks (Yona et al. 2000; Medini et al. 2006; Halary et al. 2011; Alvarez-Ponce et al. 2013; Misner et al. 2013) (fig. 1). Then, we measured average percentage identity between sequences within clusters of overlapping homologous reads (henceforth referred to as “PID”), following Halary et al. (2009, 2013) (fig. 2). We also estimated clustering coefficients, which are measures of the connectivity of the clusters (Misner et al. 2013). This network analysis allowed us to compare *Rhizophagus* reads clusters to simulated whole-genome shotgun pyrosequencing runs of fully sequenced fungal genomes with a range of genome sizes. Distributions of PID and of clustering coefficients obtained for *Rhizophagus* data were compared with the corresponding distributions obtained from these controls, so significant deviations in *Rhizophagus* with respect to simulated data could be statistically assessed.

Second, we studied genome differentiation in detail by targeting polymorphic loci inferred to be present in single copy in two *R. irregularis* isolates and one *Rhizophagus* sp*.* isolate, which are together again referred to as *Rhizophagus*. Single copy markers have previously been reported in AMF (Stukenbrock and Rosendahl 2005b), although only polymorphism of intron sequences was assessed and copy number for these markers has never been published. To develop our markers, we screened open reading frames (ORFs) in 16 fully sequenced fungal genomes. As genetic variation between sequences that are present in single copy in the genome necessarily represents genetic variation between different nuclei within the same hyphal system, this method allowed us to infer intergenomic sequence variation for specific loci.

The two approaches combined attempt to address the question of the extent and physical partitioning of genome differentiation in *Rhizophagus*. This study represents the first genome-scale approach to tackle this question in a multigenomic organism. We find evidence for genome differentiation within the *Rhizophagus* cytoplasm, both genome-wide and on the scale of a single locus. The proposed genome differentiation has important implications for *Rhizophagus* identification using genetic information, and raises questions as to how these possibly differentiated genomes function as an integrated “individual.”

## Materials and Methods

### WGS Pyrosequencing of *Rhizophagus* Isolates

Approximately 1 million sterile spores of *R. irregularis* (synonym, *G. irregulare*) isolate DAOM 197198, formulated as commercial inoculant Mycorhise ASP, were provided by Premier Tech Biotechnologies (Rivière-Du-Loup, QC, Canada) in a liquid suspension of 4,000 spores/ml. This suspension was filtered on a sterile plastic 35-µm sieve. Spores were checked for root contamination under a binocular microscope and root fragments were removed with forceps. The fungal material of isolates *R. irregularis* DAOM 234179 and *Rhizophagus* sp*.* DAOM 229456 (previously identified as *Glomus diaphanum*; Y. Dalpé personal communication) was obtained from in vitro cultures with *Agrobacterium rhizogenens* transformed carrot roots. An AMF isolate is a culture that was originally grown from a single spore. Spores and hyphae were freshly harvested by dissolving the Gellan-Gum matrix in which cultures were grown in a solution containing 0.0083 N sodium citrate and 0.0017 N citric acid, then gently crushed in a 1.5-ml microtube using a sterilized pestle. DNA was extracted using DNeasy Plant Mini kit (Qiagen), according to manufacturer’s instructions. The purified DNA was then sent to the Genome Quebec Innovation Centre (McGill University, Montréal) for pyrosequencing using the GS FLX Titanium whole-genome shotgun kit (Roche 454 Life Science), employing a full run for each DNA sample.

### Choice of Control Genomes and Pyrosequencing Simulations

To provide an internal control to interpret our sequence similarity network analysis (see below), we chose a wide range of fungal genomes with genome sizes from 15 to 150 Mb in order to cover same order of magnitude as the predicted *R. irregularis* genome sizes (Martin et al. 2008; Sędzielewska et al. 2011). To approach the 15-Mb genome size estimate, we chose genomes of *Candida albicans* (strains wo1 and sc5314) and *Candida dubliniensis* (abbreviated as wo1, sc and dub, respectively). These *Candida* genomes harbor GC contents (from 33.25% to 33.87%) close to *R. irregularis* (28%) (Tisserant et al. 2013). For the 150-Mb estimate, our simulations were based on the *Puccinia triticina, Puccinia graminis,* and *Tuber melanosporum* genomes (abbreviated as tri, gra, and tub, respectively), with GC content from 43.35% to 46.34%. Genomes were downloaded from National Center for Biotechnology Information (NCBI), or directly from the sequencing centre or genome consortium. Information regarding these genomes is summarized in supplementary table S1, Supplementary Material online. Pyrosequencing simulated data sets from these genomes, similar to our Rhizophagus WGS in terms of number of reads, length distribution, and technical bias, were performed with Metasim v0.9.1 (Richter et al. 2008). Simulation details are provided in supplementary table S2, Supplementary Material online.

### Sequence Similarity Network Analysis

As there is as yet no genome sequence available for most AMF isolates, except isolate DAOM 197198 (Tisserant et al. 2013), we used a method that allows us to describe the topology of variation in *Rhizophagus* without the need for detailed knowledge of genome content. By analogy, this approach can be likened to a restriction enzyme analysis such as restriction fragment length polymorphism (RFLP), where patterns of DNA variation are studied without knowledge of the actual genetic code. In RFLP, the same restriction enzymes are used for all DNA fragments under investigation, it becomes possible to study the relative behavior of the fragments that are cut by these enzymes. In a similar fashion, each control genome was “cut” using the sequencing parameters from actual *Rhizophagus* pyrosequencing runs to simulate exactly the same pyrosequencing run from an already published fungal genome. Subsequently, as in Misner et al. (2013), we used sequence similarity networks to cluster the real reads on the one hand, and simulated reads on the other hands, to compare the topological characteristics of these clusters (fig. 1).

All sequences sharing at least 25% identity and 75 identical nucleotides, with a BLAT *e* value cutoff of 1e-20, were grouped together following Halary et al. (2013). The resulting clusters are described by two variables. The first variable is the percentage of identical positions on the shortest sequence of an aligned reads pair (PID) (Misner et al. 2013) (fig. 2), which yields a highly conservative average percentage identity between sequence pairs in a cluster. The second is the clustering coefficient, which corresponds to the number of connected reads pairs in the cluster, divided by the maximum number of possible connections (also used in Misner et al. [2013]). This last variable quantifies similarities between sequences within a cluster of reads. The closer the clustering coefficient is to zero, the less connected, hence the more variable are the sequences in a cluster. The distributions of PID and clustering coefficient values from *Rhizophagus* and corresponding simulated reads networks were compared using a two-tailed Kolmogorov–Smirnov (KS) test.

### Annotating Singletons from the Sequence Similarity Network

To determine whether the observed differences in singleton numbers were due to differences in the functional contents of the data sets, we annotated all singletons from our sequence similarity network. FragGeneScan (Rho et al. 2010) was first used to predict and translate ORFs. The resulting protein sequences were then aligned against the Uniref90 database (Suzek et al. 2007) using BLAT (Kent 2002). UniProt90 numbers from the functional annotation were translated into KEGG Orthology (KO) numbers using the ID mapping tools on the UniProt website (www.uniprot.org , last accessed April 2013). KO numbers were mapped to KEGG pathways using the KEGG Mapper web server (www.genome.jp/kegg, last accessed April 2013). KO numbers from all (simulated) pyrosequencing runs were compared with the run with the largest number of annotated genes as distributions of annotations, which was the tub genome simulated under the parameters of the *Rhizophagus* sp*.* DAOM 229456 pyrosequencing run (which also yielded the highest number of reads). Significant differences in the proportion of functionally annotatable singletons between runs were tested with KS tests in R (www.r-project.org, last accessed April 2013).

### De Novo Identification of Repetitive DNA in *Rhizophagus* Runs and Assembled Genomes

To evaluate the repeats content of genomic data of *Rhizophagus* and control genomes, RepeatScout (Price et al. 2005) was used to generate a de novo repeats library, with default parameters and the minimum element length to report set at 50 bp. *Rhizophagus* input data consisted of the pyrosequencing reads previously described. No read sets were available for the control genomes, so we used contigs, ultracontigs or scaffolds depending on availability, with preference for the highest assembly level. Choosing the highest assembly level will yield the least repeats, and is thus a conservative estimate relative to the *Rhizophagus* data, which was only available in reads. We estimated the total number of interspersed repeats (including processed pseudogenes, retrotranscripts, Short Interspersed Elements (SINES), DNA transposons, retrovirus retrotransposons, nonretrovirus retrotransposons, Long Interspersed Elements (LINES)), simple repeats (SR), and low complexity (LC) regions in the data sets with RepeatMasker Open-3.0 (Price et al. 2005) (http://www.repeatmasker.org). The original RepeatScout library was used as a query. All parameters were set to default, except “cross_ match” as the search engine and the “slow” option, in order to obtain an increase of 0–5% in sensitivity with respect to the default parameters.

### Estimating *Rhizophagus* spp. Genome Size

Assuming that *Rhizophagus* is not genetically heterogeneous and given that the *Rhizophagus* genome cannot be shorter than the sum of the DNA segments of its nuclear DNA that do not overlap, it becomes possible to calculate the minimum length of the *Rhizophagus* genome. To do so, we added the total length of each contig, assembled using Roche Newbler 454 assembly software, to singleton lengths (which are reads that could not be assigned to a cluster). When we assume that nuclei can be genetically different within an isolate, we can estimate the length of *Rhizophagus* “pangenome,” understood as the entire collection of nuclear DNA in an isolate.

### Development of Single Copy Markers

Proteins from 16 fully sequenced fungal genomes available at the time of analysis were investigated using a pipeline of custom-made perl scripts, to find all ORFs that were present only once in all genomes. We used the genomes of Ascomycota *Ashbya gossypii*, *Aspergillus fumigatus*, *Aspergillus nidulans*, *Aspergillus niger*, *Aspergillus oryzae*, *Candida glabrata*, *Debaryomyces hansenii*, *Kluyveromyces lactis*, *Magnaporthe grisea*, *Neurospora crassa*, *Saccharomyces cerevisiae*, *Schizosaccharomyces pombe,* and *Yarrowia lipolytica*; of Basidiomycota *Cryptococcus neoformans* and *Laccaria bicolor*; and of the Microsporidia *Encephalitozoon cuniculi*. ORFs from *A. gossypii* were BLASTed against the nr database (BLASTP, threshold < 1e-5, max 5,000 hits) to retrieve homologs. Each gene was aligned with all its homologs using MUSCLE (Edgar 2004). Ambiguously aligned regions were excluded using GBlocks (Talavera and Castresana 2007) and double-checked with HoT (Landan and Graur 2007), retaining positions that were identically aligned in the reverse and forward alignments. Unambiguously aligned positions were used to reconstruct maximum-likelihood trees (applying the WAG + Gamma 4 categories model of nucleotide substitution, empirical character frequencies, estimated invariant proportion), using PHYML. These trees were scanned to define gene families in which fungi 1) were monophyletic and 2) were found in a canonical position with respect to other taxa (where “canonical” follows the phylogeny published in James et al. 2006). Reads from the *Rhizophagus* spp. runs were then aligned against these likely vertically inherited, highly conserved single copy genes. Alignment quality for selected markers was visually evaluated, applying unambiguity of alignment, sequence length, and conservation as criteria to design polymerase chain reaction (PCR) primers for 36 candidate single copy markers in *Rhizophagus*. Amplification of PCR primers was tested in the laboratory. Primer details for the retained markers are provided in supplementary table S3, Supplementary Material online.

### Relative Quantification of Marker Copy Number

To verify whether putative single copy markers were present in single copy in the *Rhizophagus* spp. genomes, we performed reverse transcription (RT)-PCR with the (monomorphic) single copy marker Rad15 as standard (Hijri and Sanders 2004; Corradi et al. 2007). Quantification of marker copy number was performed with SYBR green fluorescence for all the markers for all *Rhizophagus* isolates, and validated for a subset of the markers (40S-riboprot and Ef-tu) with TaqMan assays (Life Technologies, Canada) on *R. irregularis* DAOM 197198. The marker RLi-ABC was not validated with TaqMan assays because no sufficiently conserved region could be detected to design the probe on. Conserved fragments of marker regions were targeted using forward and reverse primer pairs and probes described in supplementary table S3, Supplementary Material online. TaqMan probes and primers were designed using Primer Express 3.0 software (Life Technologies) and synthesized by Life Technologies. In each quantitative PCR (qPCR) experiment, we deployed the same amount of DNA for the amplification of marker fragments as for the amplification of a gene that is strongly suspected to occur in single copy in the genome of *R. irregularis* DAOM 197198, Rad15 (Hijri and Sanders 2004; Corradi et al. 2007).

Total DNA was extracted from spores and hyphae using DNeasy Plant Mini Kit (Qiagen, Canada). DNA was quantified using Quant-iT PicoGreen (Life Technologies). Two-fold serial dilutions of *R. irregularis* DAOM 197198 DNA (ranging from 21 to 0.65 ng) were performed in parallel for all samples including the reference gene Rad15. qPCR was performed in three replicates, with six dilutions per replicate using iTaq Universal Probes Supermix (BioRad, Canada) for TaqMan experiments and Maxima SYBR green qPCR Mix (Fermentas, Canada) for SYBR Green experiments. qPCR reactions were performed in a 20 µl volume in ViiA7 Thermalcycler (Life Technologies). The cycle threshold (Ct values) was then plotted against the log of the DNA concentration and relative copy number was established for each sample of target DNA using the Rad15 DNA regression line as a standard.

### Amplicon Pyrosequencing of Single Copy Markers

To explore sequence polymorphism between alleles of our single copy markers within and between *Rhizophagus* spp. isolates, we performed pyrosequencing on five selected markers. Potential single copy marker sequences were amplified using DreamTaq DNA polymerase (Fermentas) using primers listed in supplementary table S3, Supplementary Material online, with suitable adapter, key, and MID sequences added. DNA from the strains *R. irregularis* DAOM 197198, *R. irregularis* DAOM 234328, and *Rhizophagus* sp. DAOM 229456 was extracted as previously described (Boon et al. 2010). The reaction was performed in 50 µl, containing 1 ng DNA, 1.25 U Taq polymerase (Fermentas), 0.2 mM dNTPs, 0.4 µM of each primer, and the PCR buffer. PCR was carried out for 40 cycles, that is, 94 °C for 30 s, Ta for 30 s (see supplementary table S3, Supplementary Material online, for annealing temperature per primer pair) and 72 °C for 1 min, which were preceded by an initial 3-min denaturation at 95 °C and followed by a 10-min hold at 72 °C, on a Mastercycler EPgradient S (Eppendorf). The PCR product was checked on an electrophoresis gel to ensure successful amplification of the gene, and then purified using a MinElute PCR Purification Kit (Qiagen) according to manufacturer’s instructions. These purified samples were pooled by molecular weight and sent to the Genome Quebec Innovation Centre (McGill University) for pyrosequencing using the GS FLX Titanium emPCR kit (Roche 454 Life Science) with lib-L chemistry in one-eighth run.

### Analysis of Pyrosequencing Results for Single Copy Markers

All analyses were performed using Mothur v. 1.22 (Schloss et al. 2009), unless specified otherwise. Low-quality reads were eliminated according to previously published guidelines (Huse et al. 2007; Schloss et al. 2011); eliminated reads included those that 1) did not perfectly match the adaptor and primer sequences, 2) had ambiguous bases, 3) had a quality score below an average of 35 in a window of 50 bp, and 4) contained homopolymer lengths greater than 8 bp. Reads that passed quality control and differed by just 1 bp were preclustered following Huse et al. (2010). Chimeric molecules that could have formed during the PCR (Wang GCY and Wang Y 1997) or pyrosequencing (Haas et al. 2011) steps were defined as reads that did not match a database of previously obtained (Sanger sequenced) sequences with less than 90% bootstrap support. Chimeric sequences were detected and removed using the program Chimeraslayer (Haas et al. 2011). To assess whether sampling was representative of the actual diversity, we performed rarefaction analyses for every marker. We calculated the total number of alleles and the Chao1 diversity index, which measures the minimum richness in a sample (Chao et al. 2005). Only alleles that occurred more than once in the data set were considered.

Sequences were translated into amino acids by comparing them with homologous loci from the 16 fungal genomes that were originally used to find single copy markers in *Rhizophagus* spp. genomes. Recombination rate was calculated in DnaSP 5.10.01 (Librado and Rozas 2009) with the Rm estimator (Hudson and Kaplan 1985).

To assess whether our clustering of the data into alleles was the most appropriate approach to minimize the influence of sequencing error on our conclusions, we also tested three alternative clustering or denoising strategies, that is, preclustering by 2 bp differences instead of one, denoising of the sequences through SeqNoise as implemented in Mothur v1.28, and denoising of the flow files through AmpliconNoise (Quince et al. 2011). As error rate varies per run, we estimated per run error rate based on sequencing errors from Roche’s internal homopolymer controls, which were kindly provided by the Genome Quebec Innovation Centre (McGill University).

### Validation of Polymorphism

To investigate the possibility that single copy marker polymorphism was due to the sequencing methodology, we cloned and Sanger sequenced the TaqMan qPCR products of the 40S-riboprot, Ef-tu, and ARP markers. An amount of 4 µl qPCR product was ligated into pGEM-T easy vector (Promega, Canada) and transformed into *Escherichia coli* competent DH5alfa cells. Bacterial colonies were randomly picked and PCR-screened with universal T7 and SP6 vector primers. Bacterial colonies that showed a PCR product with the expected size were considered as positive clones and were sent for sequencing to the Genome Quebec Innovation Centre (McGill, Canada).

### Data Deposition

All raw pyrosequencing data (amplicon data for the single copy markers and Whole Genome Sequencing (WGS) for the evolutionary network analyses) were deposited in the NCBI Trace Archive under Bioproject number PRJNA174749. The single copy marker allele alignments are provided in the supplementary material, Supplementary Material online.

## Results

### Genome-Wide Diversity Patterns through Simulations and Clustering

**Fig. 3.—:**
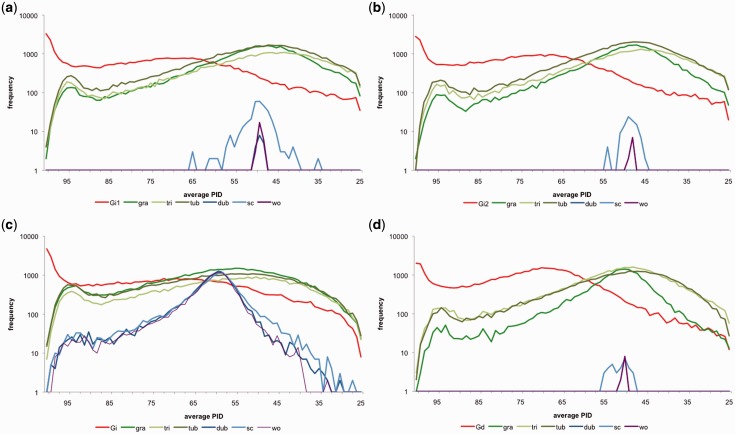
Reads clusters frequencies compared with average PID per cluster. For all *Rhizophagus* and control genomes (*C. albicans* wo1; wo1, *C. albicans* sc5314; sc, *C. dubliniensis*; dub) with (*a*) *R. irregularis* DAOM 234179 run: Gi1; (*b*) *R. irregularis* DAOM 234179 run: Gi2; (*c*) *R. irregularis* DAOM 197198: Gi; (*d*) *Rhizophagus* sp*.* DAOM 229456: Gd.

**Fig. 4.—:**
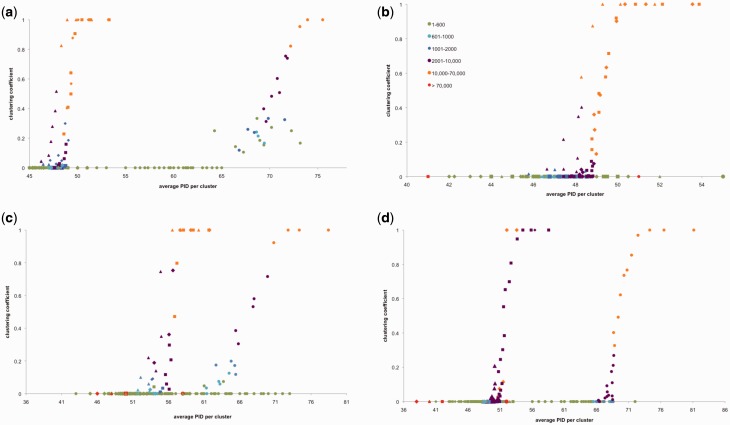
Scatterplots depict average PID in a similarity cluster and clustering coefficient. For all *Rhizophagus* spp. and control genomes, (*a*) *R. irregularis* DAOM 234179 run 1, (*b*) *R. irregularis* DAOM 234179 run 2, (*c*) *R. irregularis* DAOM 197198, and (*d*) *Rhizophagus* sp. DAOM 229456. Colors correspond to the number of reads that are represented by the respective data-points (see legend; for simplicity, only one legend is depicted for all graphs). The shape of the data-point refers to the reads set: Circle, *Rhizophagus* (specific isolates are identified in the graph title); square, *P. graminis*; diamond, *T. melanosporum*; triangle, *P. triticina*.

We estimated 1) the PID distribution and 2) clustering coefficients (where a high clustering coefficient indicates high overlap between sequences), following Halary et al. (2009, 2013) and Misner et al. (2013), see Materials and Methods and figures 1 and 2.We plotted the frequency distribution of average PID per cluster from each (real and simulated) pyrosequencing run (figs. 3 and 4). PID distributions from *C. albicans* sc5314, *C. albicans* wo1, and *C. dubliniensis* (from now on referred to as small control genomes) were significantly different from both *Rhizophagus* and tri, gra and tub (from now on referred to as large control genomes) (supplementary table S4, Supplementary Material online). PID distributions from *Rhizophagus* pyrosequencing runs were not significantly different from those based on simulations from large control genomes. However, *Rhizophagus* PID distributions showed much elevated numbers of reads between 95% and 100% PID in comparison to the large control genomes. We also plotted clustering coefficients against average % PID per cluster (fig. 4). Except in the case of *R. irregularis* DAOM 234179 run 2, each analysis showed a higher % PID for the same clustering coefficient in *Rhizophagus* reads clusters, meaning that there is more sequence overlap in these data sets than in controls.

An interesting exception was the case of the gra simulated data set, which contained the largest amount of data (1,078,190 reads, table 1) (fig. 3*d*). The PID distribution was significantly different from the other large control genomes and *Rhizophagus* (KS test, *D* = 0.3168 [comparison with *Rhizophagus* sp. and tub] and 0.3267 [comparison with tri], *P* values 7.91E-05 and 4.15E-05, respectively, see also supplementary table S4, Supplementary Material online). The difference between the distributions for this *Rhizophagus* sp. run and for gra, the smallest of the large control genomes genome (88 Mb, supplementary table S1, Supplementary Material online) reveals an important property of our approach. It shows that sequencing coverage plays an important role in the resolution of our network analyses. When the sampling depth of the real and simulated pyrosequencing runs approaches the actual size of the sampled genome, the sequence similarity network approach starts differentiating small and large genomes. In the case of gra, the genome size is 88 Mb and the pyrosequencing depth is 1,078,190 reads with an average length of 336 bp (supplementary tables S1 and S2, Supplementary Material online). Thus, a mere 4-fold coverage seems sufficient to start inferring differences between *Rhizophagus* and some of the large control genomes.

**Table 1:** Results from Clustering Analyses for *Rhizophagus* spp. and Control Genomes

Strain	No. of Clusters	Average No. of Reads/Cluster	Average PID	SD	Average Clustering Coefficient
*Rhizophagus irregularis* DAOM 234179 run1 (485,491 reads)					
Gi1	37,729	11	76	11	0.87
gra	38,620	11	51	11	0.72
tri	33,862	10	50	7	0.82
tub	49,868	9	51	9	0.8
dub	17	28,056	49	19	0.03
sc	340	1,402	50	18	0.28
wo1	30	15,901	49	19	0.05
*R. irregularis* DAOM 234179 run 2 (639,222 reads)					
Gi2	40,221	14	76	13	0.84
gra	33,046	17	51	13	0.66
tri	36,429	13	50	9	0.79
tub	52,391	11	51	11	0.74
dub	1	630,117	47	18	0
sc	100	6,300	49	18	0.16
wo1	12	52,509	48	18	0.07
*R. irregularis* DAOM 197198 (398,817 reads)					
Gi	44,989	6	76	7	0.9
gra	53,315	5	60	6	0.84
tri	33,851	6	59	4	0.87
tub	44,780	6	60	4	0.89
dub	11,795	32	61	17	0.37
sc	11,934	32	61	16	0.42
wo1	10,932	35	61	17	0.35
*Rhizophagus* sp. 229456 (1,078,190 reads)					
Gsp	46,087	22	75	14	0.77
gra	20,125	48	53	16	0.53
tri	37,849	21	53	11	0.72
tub	33,908	17	52	11	0.51
dub	1	1,060,812	49	18	0
sc	33	32,150	51	19	0.18
wo1	12	88,399	50	19	0.06

Note.—wo1, *Candida albicans* wo1; sc, *C. albicans* sc5314; dub, *C. dubliniensis*.

### Estimating Genome Size with Sequence Similarity Networks

Clustering reads from control genomes resulted in very few clusters with many reads on the smaller *Candida* control genomes. This result is expected and consistent with analyses in Misner et al. (2013). We propose that these few clusters correspond to supercontigs of the *Candida* genomes, covering very large segments of these genomes. These “superclusters” are formed more readily in *Candida* genomes, as their small size leads to a high coverage faster than in the larger genomes. Therefore, our clustering approach effectively gathered large segments of the smaller control genomes by identifying overlapping reads. In contrast, our pyrosequencing efforts did not lead to a similar clustering for the large control genomes and for *Rhizophagus*. As *Candida* and *Rhizophagus* genomes share similar GC contents, unlike large control genomes, the effect of the pyrosequencing data set sizes (and thus, coverages) is likely much greater on clustering differences than a GC bias.

Because no *Rhizophagus* genome was thus fully “assembled,” we used the total length of the assembled contigs plus that of singletons as a conservative estimate of the *Rhizophagus* genome (or pangenome) size. The sum of all contigs and singletons for *R. irregularis* DAOM 234179 was 178 Mb (based on an assembly comprising both run 1 and run 2), 163.7 Mb for *Rhizophagus* sp*.* DAOM 229456, and 64.7 Mb for *R. irregularis* DAOM 197198. These sums could be considered minimum genome sizes only in the sense that they are a sum of all overlapping and nonoverlapping genetic variation that a single pyrosequencing run retrieves. In the light of the heterokaryosis hypothesis, these minimum genome sizes do not give us information on whether the variation is located within or between nuclei. In principles, we may have inferred a conservative size for the pangenome of an isolate, and genome size of individual nuclei within such isolate may still vary.

### Annotating Singletons

We annotated all singletons from *Rhizophagus* runs and from the large control genome simulated runs, for which distributions of PID and clustering coefficient are most similar to our *Rhizophagus* pyrosequencing runs. Initial numbers of singletons varied between 4,474 for the tub-based simulation under the parameters of *R. irregularis* DAOM 234179 run 2 and 496,891 for the tub-based simulation under the parameters of *Rhizophagus* sp. DAOM 229456. Singletons could be assigned to ORFs and annotated (supplementary fig. S1, Supplementary Material online) with comparable success between *Rhizophagus* and simulated runs (Wilcoxon Signed Rank Test, supplementary table S5, Supplementary Material online). Thus, *Rhizophagus* singletons do not consist of less ORFs than singletons from the large control genomes. We compared the KEGG annotations of the singletons, and tested the significance of differences between annotation distributions with KS tests. There were no differences between annotated singletons from *Rhizophagus* and control runs: Only the tub- and gra-based simulations under the parameters of *Rhizophagus* sp. DAOM 229456 were different from the other (simulated) runs (supplementary table S6, Supplementary Material online). Thus, annotation content, based on KEGG hierarchies, between *Rhizophagus* singletons and the large control genomes did not change with genome used as a basis for the (simulated) run.

### De Novo Identification of Repetitive DNA in *Rhizophagus* Runs and Assembled Genomes

We estimated GC content and the percentage of masked bases for pyrosequencing runs of our *Rhizophagus* and for the control genomes (supplementary table S7, Supplementary Material online). *Rhizophagus* runs showed a typical low GC percentage (Hosny et al. 1997; Tisserant et al. 2013). They also showed a higher percentage of masked bases, although isolate DAOM 197198 fell in the lower percentage of masked bases and was in this respect similar to tri. SR were higher for *Rhizophagus* than for the larger control genomes, but lower with respect to the small genome control data sets. Finally, *Rhizophagus* runs showed a slightly higher percentage of LC regions with respect to all control genomes. *Rhizophagus* isolate DAOM 197198 actually has less masked bases than two of the control genomes, gra and tub.

### Allele Diversity Estimates within *Rhizophagus* spp. Isolates

Our second approach to study genome diversity in AMF is a detailed investigation of polymorphism within and between single copy markers. We developed and pyrosequenced five novel markers, which all represent partial exons from protein-coding sequences. Between 90 and 1,123 sequences fulfilling stringent quality criteria were recovered per marker (table 2). All markers were polymorphic, yielding between 2 and 103 alleles. Rarefaction curves are reported in supplementary figure S2, Supplementary Material online. Preclustering at different levels had an effect on allele count, but denoising strategy did not (supplementary table S8, Supplementary Material online). The closest database matches for these markers after three psi-BLAST iterations (pBLAST search of translated marker sequences against nr, cutoff E-25) are detailed in supplementary table S9, Supplementary Material online. Markers were named after the closest psi-BLAST hit.

**Table 2:** Amplification and Genetic Diversity of Single Copy Markers

	Diversity over All Strains^a^						
Marker Name	No. of Sequences	Alleles	Alleles (*n* > 1)^b^	Chao1^c^	lci	hci	Final Alignment (bp)
RLi-ABC	299	4	3	7	4	28	68
ARP	556	17	15	122	56	298	99
ACOB	768	2	2	2	2	2	21
40S-riboprot	92	11	8	39	18	116	175
Ef-tu	1,123	203	103	294	258	352	197

^a^*R. irregularis* DAOM 197198; *R. irregularis* DAOM 234328; *Rhizophagus* sp*.* DAOM 229456.

^b^Number of alleles that occur more than only once in the data set.

^c^Chao1 index; lci, lower 95% confidence interval; hci, higher 95% confidence interval (Chao et al. 2005).

### Testing for Copy Number and Polymorphism Validation

**Fig. 5.—:**
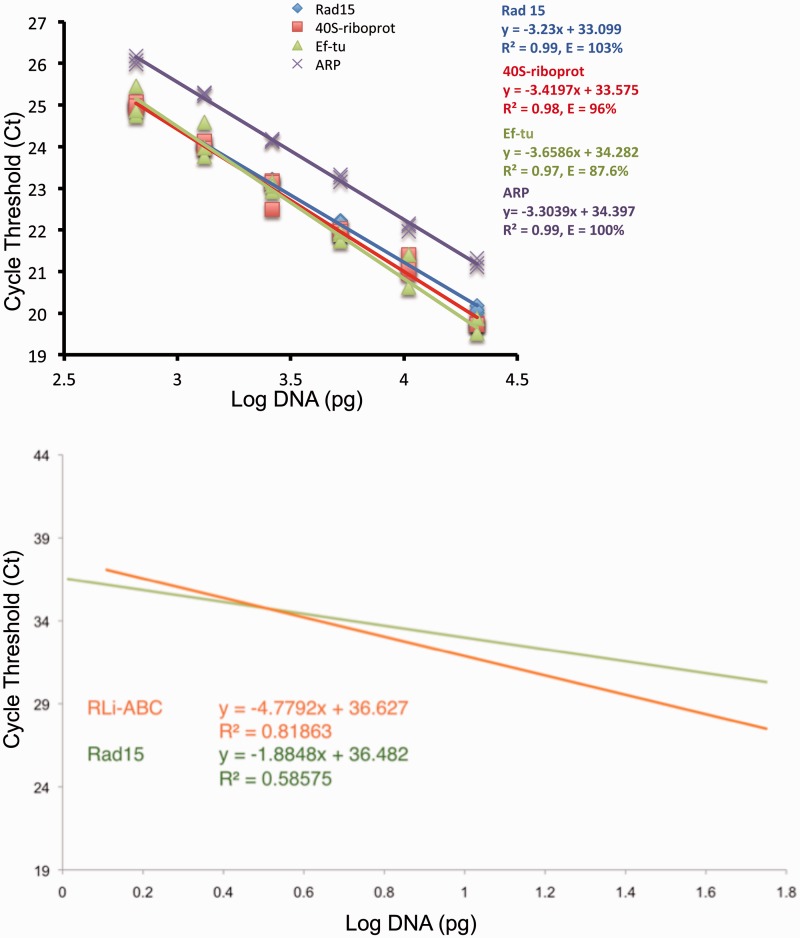
Results of real-time qPCR for single copy markers. Linear regressions of the cycle threshold (Ct values) and the Log concentration of *R. irregularis* DAOM 197198 genomic DNA (pg) that was used as template for the reaction using TaqMan assays. Ct values of the markers Ef-tu and 40S-riboprot were compared with Ct values of the marker Rad15.

Copy number for the five markers was tested by qPCR. Markers Ef-tu, 40S-riboprot, and RLi-ABC showed similar linear regressions of Ct values than the reference gene Rad15 (fig. 5 and supplementary fig. S3, Supplementary Material online). This indicates that these three markers have similar copy number than Rad15, which is likely a single copy gene. A *R. irregularis* genome search confirmed the occurrence of one copy of Rad15 sequence. Surprisingly, the markers ARP and ACOB showed approximately 2-fold higher Ct values compared with Rad15. Thus, in both TaqMan (ARP) and SYBR Green (ACOB) assays, the markers ARP and ACOB seem to be present in less than one copy per genome (fig. 5 and supplementary fig. S3, Supplementary Material online).

We validated the polymorphism observed in the amplicon pyrosequencing runs for the markers 40S-riboprot, Ef-tu, and ARP, by cloning and Sanger sequencing of the TaqMan qPCR products. In spite of the low sampling intensity yielded by cloning/sequencing, most abundant alleles for these markers were recovered (supplementary table S10, Supplementary Material online).

## Discussion

### Excess of Strongly Similar Coding DNA Regions in *Rhizophagus*

PID distributions from the small control genomes were significantly different from both *Rhizophagus* and large control genome distributions (fig. 3 and supplementary table S4, Supplementary Material online). Thus, our sequence similarity network approach suggests that *Rhizophagus* genomes behave as a large genome. We predicted *Rhizophagus* minimum genome sizes. They range from 64.7 Mb for *R. irregularis* DAOM 197198 to 163.7 Mb for *Rhizophagus* sp. DAOM 229456 and 178 Mb for *R. irregularis* DAOM 234179, which all fall in the size range of the larger genomes. The most recent genome size estimates for *R. irregularis* also fall in this order of magnitude (Sędzielewska et al. 2011; Tisserant et al. 2013). It is important to note that comparing *Rhizophagus* to fungi with large genomes does not provide any information on the location of the variation: Clusters of reads and singletons on which these size estimates are based could be located within or between different nuclei in the cytoplasm.

The PID distributions of *Rhizophagus* show more reads clusters with higher average sequence identities (PID) than simulated genomes (fig. 3). Furthermore, reads clusters from *Rhizophagus* genomes have higher average PID than clusters of reads from control fungi (table 1). This excess of “strongly similar” regions (PID > 95%) in *Rhizophagus* isolates suggests that the genetic organization in *Rhizophagus* differs from that in the control fungi. How can we interpret the excess of strongly similar regions in *Rhizophagus* spp. isolates? The reads from clusters that are characterized by a high average PID can come from the same or from different nuclei within the same cytoplasm. They can represent conserved coding genome regions in *Rhizophagus*, or noncoding repetitive elements (NCRE), such as tandem repeats. We propose that a large portion of the clusters that are characterized by a high average PID (>95%) come from conserved coding genome regions located in different nuclei.

Indeed, for any cluster of reads, a high clustering coefficient indicates that a similar sequence is repeated multiple times in the cluster, whereas a low clustering coefficient indicates a cluster that is more comparable to a contig (e.g., a succession of overlapping reads, see also fig. 4). For the same average PID per cluster, *Rhizophagus* clusters have a higher clustering coefficient than clusters from the control genomes (fig. 4). Thus, nuclei in *Rhizophagus* isolates contain more highly similar regions, and a greater redundancy than the control fungi.

To get an idea of cluster content, we looked at repeat content in clusters. We compared NCRE in clusters from *Rhizophagus* and control genomes. NCRE numbers in *Rhizophagus* were not substantially different between large control and *Rhizophagus* genomes (supplementary table S7, Supplementary Material online). Our current estimate of 45% aligns more closely with findings from the *Rhizophagus* genome (Tisserant et al. 2013). NCRE numbers are also high in the large control genomes (supplementary table S1, Supplementary Material online), so the peak *Rhizophagus* isolates show at greater than 95% PID cannot be explained by NCRE. Moreover, there were no differences in the proportions of annotated singletons between *Rhizophagus* genomes and large control genomes, indicating that all these genomes have comparable amounts of coding material (supplementary fig. S1 and table S8, Supplementary Material online)

Thus, we find redundant clusters of highly similar (although not identical) coding sequences in the *Rhizophagus* pyrosequencing runs. However, with our current data, it is not possible to assess whether these redundant coding sequences occur within or between nuclei in the same cytoplasm. Therefore, we cannot differentiate between highly conserved multigene families within the same nucleus, which would fit the homokaryosis hypothesis, or repeated low copy genes that are partitioned between nuclei, which would fit the heterokaryosis hypothesis. To distinguish between these scenarios, we have to look in detail at selected single copy markers.

### *Rhizophagus* Genome Variation in Close-Up with Single Copy Markers

**Fig. 6.—:**
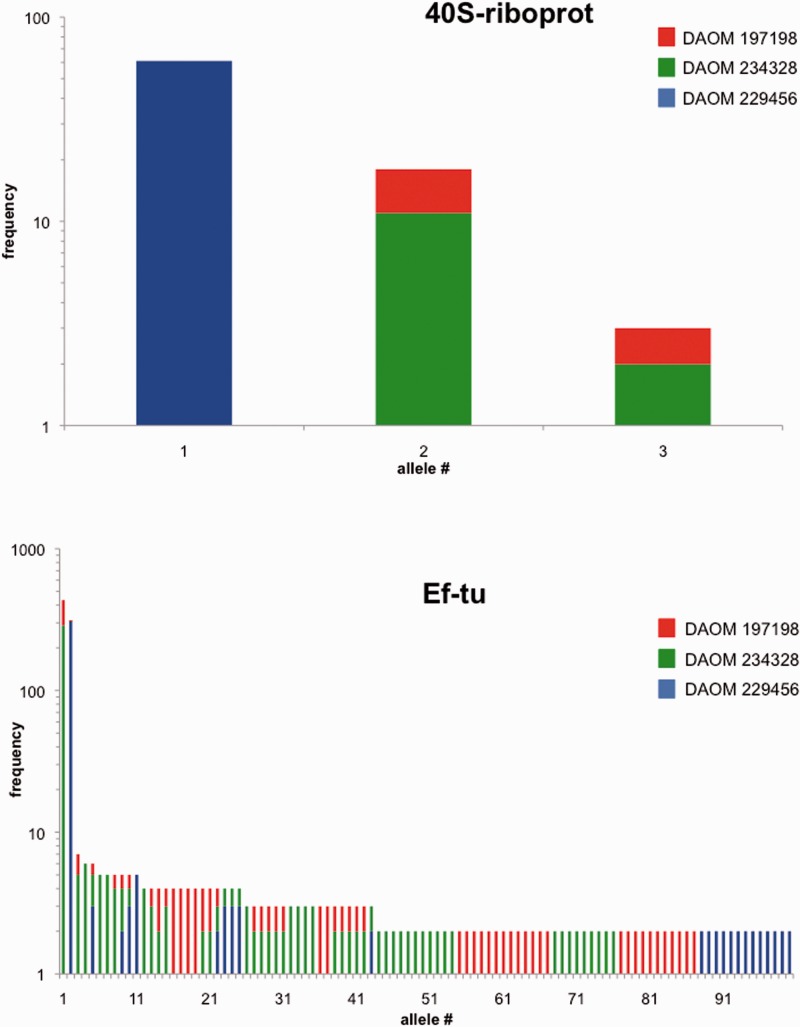
Allele distributions for selected single copy markers (*a*) 40S-riboprot and (*b*) Ef-tu (only alleles for which *f* > 1 are depicted).

This study reports extensive polymorphism on protein-coding single copy markers in AMF. We retrieved between 2 and 103 alleles for each putative single copy marker (table 2). Differentiation is not homogenous between loci: Some markers yielded relatively few variants (marker ACOB), whereas others revealed over a hundred different alleles (e.g., marker Ef-tu, see fig. 6*a* for the allele distribution). Our observations concur with previous findings of genetic differentiation between loci in AMF nuclei within the same cytoplasm (Kuhn et al. 2001; Hijri and Sanders 2005; Boon et al. 2010; Ehinger et al. 2012; Tisserant et al. 2012).

Importantly, the allele counts we report most likely underestimate the total allele diversity in the isolates used in this analysis, for three reasons. First, none of the approaches we used to estimate minimum allele diversity for the *Rhizophagus* isolates reached a plateau of diversity, as defined by 1) rarefaction analyses (supplementary fig. S2, Supplementary Material online) and 2) the lower confidence interval of the Chao1 diversity index (table 1). For four of five markers (with the exception of the marker ACOB), the rarefaction analyses and Chao1 diversity index both indicate a spectrum of rare alleles that remains to be sampled. Second, we applied stringent quality checks, and reads differing by only 1 bp were clustered together. This clustering artificially lowers variation in a data set by erring on the conservative side. Three, we only counted alleles that occurred more than once in the reads from the pyrosequencing runs.

Although our conservative approach increases the chance of lumping different alleles together, this approach is necessary to avoid the inflated diversity counts that are often associated with pyrosequencing technology (Huse et al. 2010; Schloss 2010; Haas et al. 2011; Schloss et al. 2011; Schloss and Westcott 2011). More stringent preclustering is not expected to yield more stringent results: The error rate of our specific amplicon pyrosequencing run was 0.6%, which is lower than the amount of variation we removed by preclustering. Incidentally, this rate is also lower than the mean rates reported for this sequencing technology (Gilles et al. 2011). Finally, more stringent preclustering would compromise our ability to separate signal from noise. To elaborate on this point, if we would cluster reads differing by 2 bp together, the maximum amount of differences within a cluster would be 4 bp. On the length of one of the longest alignments, that for 40S-riboprot (175 bp), 4 bp represents 2.3% of the total sequence. As this level is typically the level of variation we are interested in, it thus becomes difficult to start inferring patterns of variation from the data. On shorter alignments, this effect would be even more pronounced. To actually distinguish signal from noise, one would need to implement denoising algorithms based on sequence or flow (.sff) data such as SeqNoise or AmpliconNoise, respectively. Applying these denoising algorithms, we mostly find the same number or more alleles in our data (supplementary table S8, Supplementary Material online). Thus, we are confident that we have underestimated and not overestimated the number of alleles for each single copy marker.

Another caveat might be the use of Rad15 as a single copy reference marker. Rad15 showed exactly the same pattern in RT-PCR experiments as Rad32 (Corradi et al. 2007), which was shown to be genetically homogeneous and likely present in single copy in isolate DAOM 197198 by a dot blot hybridization assay (Hijri and Sanders 2004). Therefore, our reference gene was the best available choice for estimation of relative copy number. It should be noted that copy number estimations in AMF can only be considered as an average over all mycelia, as it is possible that copy number variations occur between *R. irregularis* isolates as demonstrated by Corradi et al. (2007) for rRNA genes. However, we attempted to negotiate this difficulty by special precautions in the prescreening phase, through 1) our bioinformatics approach, in which we have excluded all loci that show signs of occurring in multiple copies in the *Rhizophagus* genome data or in any other fungal genome; 2) excluding all candidate markers that showed major deletions or rearrangements in the sequence alignment, which could be chimerical due to in vivo or in vitro recombination; and 3) only focusing on the most conserved parts of the loci under investigation.

If we consider the possibility of heterokaryosis, how could the polymorphism observed between single copy markers in the same AMF isolate (i.e., in the same cytoplasm) be maintained? Fusion between hyphal systems, anastomosis, could play an important role in conserving polymorphism between hyphal networks in soil ecosystems (Croll et al. 2009; Marleau et al. 2011; Boon et al. 2013). Little is known about generation and loss of genetic variation within AMF isolates. The possible effects of genetic drift have been described in AMF isolates (Cárdenas-Flores et al. 2010; Angelard et al. 2010; Angelard and Sanders 2011; Ehinger et al. 2012; Boon et al. 2013; de la Providencia et al. 2013). Alternatively, differences between allele frequencies might be due to differential selection pressures on the loci themselves or on the adjacent genome regions of the single copy markers. Unfortunately, we cannot test this latter hypothesis on our data, as preclustering reads from pyrosequencing technology means that all allele sequences are consensus sequences. Thus, variation between sequences cannot be used to confidently infer deviations from neutrality. Deviations from neutrality between loci are possible if linkage equilibrium is interrupted by recombination, as we observed for three of five loci (table 3), and as has been previously been reported in AMF (Vandenkoornhuyse et al. 2001; Gandolfi et al. 2003; Croll and Sanders 2009). However, the investigation of recombination and linkage in AMF is precarious, as there are few databases of sufficiently long reads available for any AMF.

**Table 3:** Stopcodons, Frameshift mutations and Recombination in Single Copy Markers

Marker	No. of Seqs	Nucleotide Positions^a^	Stopcodons^b^	Frameshift	Rm^c^
RLi-ABC	299	67	0	0	0
ARP	550	96	1	5	2
ACOB	768	21	0	0	0
40S-riboprot	92	175	0	0	1
Ef-tu	1,071	191	34	18	7

^a^Number of nucleotide positions used in analysis.

^b^Stopcodons and frameshift events were counted before being removed from the analysis.

^c^Minimum number of recombination events (as implemented in Librado and Rozas [2009]).

*Significant at α = 0.05.

This latter point stresses the potential importance of a single copy marker approach, as databases of variation between these or other single copy markers could be implemented in a manner very similar to multilocus sequence typing (MLST) to answer questions of linkage and recombination (Maiden et al. 1998). A major advantage of this strategy would be the possibility to study sequence polymorphism in AMF without assumptions on the inter- or intranuclear localization of this variation.

### Probing the *Rhizophagus* Genome

We suggest that each *Rhizophagus* isolate harbors a population of differentiated genomes, based on observations from the two approaches presented in this study. First, we argue that differences in PID distributions between *Rhizophagus* and the large control genomes point to the presence of many similar but slightly differentiated sequences in the AMF cytoplasm (figs. 3 and 4).

Second, our observations of extensive genomic heterogeneity in *Rhizophagus* single copy markers indicate that this variation could be partitioned between and not within nuclei (and thus genomes) in the *Rhizophagus* cytoplasm. These observations agree with previous findings of genetic differentiation between specific loci in AMF nuclei (Kuhn et al. 2001; Hijri and Sanders 2005; Boon et al. 2010; Ehinger et al. 2012; Tisserant et al. 2012). Even though alleles from different loci found within AMF isolates have already been shown to be physically located between nuclei using FISH (Kuhn et al. 2001; Kuhn 2003), our reports of genetic heterogeneity between loci are the first to provide direct evidence of differentiation between genomes from single copy markers.

### Some Speculation on the *Rhizophagus* Genome

The redundant clusters of slightly differentiated reads in our sequence similarity network analysis and high levels of polymorphism for our putative single copy genes do not align with the homokaryosis hypothesis, even though some may still find the evidence provided here insufficient to support the heterokaryosis hypothesis. However the spatial organization of genetic variation in the AMF isolates under study, we can report with certainty on the unusually high polymorphism that we recovered. Even with a one-eighth pyrosequencing run we have not attained saturation of allele diversity in this study—a telltale of much variation we are still missing. Therefore, we suggest that each *Rhizophagus* isolate harbors a population of (at least partly) genetically differentiated genomes. If so, populations of nuclei within the AMF cytoplasm may act together to produce the *Rhizophagus* phenotype. Four observations support this interpretation. First, for several AMF it has been shown that they are at no point in their life cycle reduced to a single genome (Jany and Pawlowska 2010; Marleau et al. 2011; Boon et al. 2013). Second, *Rhizophagus* spores do not germinate below a certain number of nuclei per spore, which is roughly 65 nuclei for *R. irregularis* (Marleau et al. 2011). Third, for *R. irregularis* and *G. etunicatum* it was shown that genetic polymorphism is expressed in the transcriptome (Boon et al. 2010; Tisserant et al. 2012), which indicates that differentiation at the genome level could play a role in the functioning of *Rhizophagus* isolates. Finally, the high amounts of genetic variation in AMF isolates have been proposed to play a role in the ability of AMF to adapt to a wide range of host plants (Angelard et al. 2010).

Accordingly, we propose that this population of partly heterogeneous genomes in AMF is analogous to a pangenome, as there may not be one typical genome within an isolate, representative of all the other, but rather a population of partly differentiated genomes. The minimum size of the *Rhizophagus* pangenome would then be around 65 Mb for *R. irregularis* DAOM 197198, 178 Mb for *R. irregularis* DAOM 234179, and 163.7 Mb for *Rhizophagus sp.* DAOM 229456. These estimates are closer to the upper limit of the *R. irregularis* genome size that has been published to date, that is, 150 Mb (Martin et al. 2008; Sędzielewska et al. 2011; Tisserant et al. 2013), than to a previously published lower estimate of 15 Mb (Hijri and Sanders 2004).

The recognition of unprecedentedly high levels of genetic diversity within the *Rhizophagus* cytoplasm reported here and the possible organization of this genetic diversity into differentiated nuclei could lead to a careful consideration of the concept of the individual (Santelices 1999; Pineda-Krch and Lehtila 2004; Pepper and Herron 2008; Folse and Roughgarden 2010) in *Rhizophagus*, with important consequences for genetically based AMF studies in agriculture and ecology. A particularly exciting avenue is the role of anastomosis in the maintenance of this genetic variation, which could be tested by an MLST approach on multiple *Rhizophagus* isolates. Comparing interisolate single copy marker diversity profiles, such as those reported in this study, will allow AMF researchers to study linkage and recombination between AMF isolates. Microbial ecology has already developed many metagenomics tools that can be used to study the evolution, function, and stability of a community whose components cannot be traced individually. We hope that this study will open similar avenues to the study of AMF.

## Supplementary Material

Supplementary material, figures S1–S3, tables S1–S10, and references are available at *Genome Biology and Evolution* online (http://www.gbe.oxfordjournals.org/).

Supplementary Data
