# Supplementary material for: Studying Genome Heterogeneity within the Arbuscular Mycorrhizal Fungal Cytoplasm
Source: Genome Biol Evol. 2015 Jan 7;7(2):505–21. doi: 10.1093/gbe/evv002 (PMC4350173; doi:10.1093/gbe/evv002)
Supplement: Supplementary Data [file supp_7_2_505__index.html]

Studying Genome Heterogeneity within the Arbuscular Mycorrhizal Fungal Cytoplasm — Supplementary Data 

# Studying Genome Heterogeneity within the Arbuscular Mycorrhizal Fungal Cytoplasm

## Supplementary Data

files

**Files in this Data Supplement:**

- Supplementary Data - pdf file
- Supplementary Data - pdf file
