# Supplementary material for: Studying Genome Heterogeneity within the Arbuscular Mycorrhizal Fungal Cytoplasm
Source: Genome Biol Evol. 2015 Jan 7;7(2):505–21. doi: 10.1093/gbe/evv002 (PMC4350173; doi:10.1093/gbe/evv002)
Supplement: Supplementary Data [file supp_evv002_single.copy.marker.alignments.pdf]

#40S_riboprot

>GZ2VM8F07H0SB1
CGTCTAGTCAAAGAAAACAAGATCAAAACCATTGAAGAAATATATTTATATTCATTT
CCCATCAAAGAATTTCAAATCGTAGATCGTTTAATCAGCACTACATTAAAAGATGAA
GTCA-
TGAAGATTATGCCCGTTCAAAAGCAGACCCGTGCTGGTCAGAGGACTCGATTCAAGG
>GZ2VM8F07IO0AE
CGTCTAGTCAAAGAAAACAAGATCAAAGCCATCGAAGAAATATATTTATATTCATTT
CCCATCAAAGAATTTCAAATCGTAGATCGCTTAATCAGCACTACATTAAAAGATGAA
GTCA-
TGAAGATTATGCCCGTTCAAAAGCAGACCCGTGCTGGTCAGAGGGCTCGATTCAAGG
>GZ2VM8F07IGVTD
CGTCTAGTCAAAG-
AAACAAGATCAAAACCATCGAAGAAATATATTTATATTCATTTCCCATCAAAGAATT
TCAAATCGTAGATCGCTTAATCAGCACTACATTAAAAGATGAAGTCA-
TGAAGATTATGCCCGTTCAAAAGCAGACCCGTGCTGGTCAGAGGACTCGATTCAAGG
>GZ2VM8F07ISGEZ
CGTCTAGTCAAAGAAATCAAGATCAAAACCATTGAAGAAATATATTTATATTCATTT
CCCATCAAAGAATTTCAAATCGTAGATCGTTTAATCAGCACTACATTAAAAGATGAA
GTCA-
TGAAGATTATGCCCGTTCAAAAGCAGACCCGTGCTGGTCAGAGGGCTCGATTCAAGG
>GZ2VM8F07IPNMV
CGTCTAGCCAAAGAAAACAAGATCAAAACCATTGAAGAAATATATTTATATTCATTT
CCCATCAAGGAATTTCAAATCGTAGATCGTTTAATCAGCACTACATTAAAAGATGAA
GTCA-
TGAAGATTATGCCCGTTCAAAAGCAGACCCGTGCTGGTCAGAGGACTCGATTCAAGG
>GZ2VM8F07H3HM2
CGTCTAGTCAAAGAAAACAAGATCAAAACCATCGAAGAAATATATTTATATTCATTT
CCCATCAAAGAATTTCAAATCGTAGATCGCTTAATCAGCACTACATTAAAAGATGAA
GTCA-
TGAAGATTATGCCCGTTCAAAAGCAGACCCGTGCTGGTCAGAGGACTCGACTCAAGG
>GZ2VM8F07IO214
CGTCTAGTCAAAGAAAACAAGATTAAAACCATCGAAGAAATATATTTATATTCATTT
CCCATCAAAGAATTTCAAATCGTAGATCGCTTAATCAGCACTACATTAAAAGATGAA
GTCA-
TGAAGATTATGCCCGTTCAAAAGCAGACCCGTGCTGGTCAGAGGACTCGATTCAAGG
>GZ2VM8F07IETYX
CGTCTAGTCAAAGAAAACAAGATCAAAACCATCGAAGAAATATATTTATATTCATTT
CCCATCAAAGAATTTCAAATCGCAGATCGCTTAATCAGCACTACATTAAAAGATGAA
GTCA-
TGAAGATTATGCCCGTTCAAAAGCAGACCCGTGCTGGTCAGAGGACTCGATTCAAGG
>GZ2VM8F07IILLT
CGTCTAGTCAAAGAAAACAAGATCAAAACCATCGAAGAAATATATTTATGTTCATTT
CCCATCAAAGAATTTCAAATCGTAGATCGCTTAATCAGCACTACATTAAAAGATGAA
GTCA-
TGAAGATTATGCCCGTTCAAAAGCAGACCCGTGCTGGTCAGAGGACTCGATTCAAGG

>GZ2VM8F07IR914
CGTCTAGTCAAAGAAAACAAGATCAAAACCATCGAAGAAATATATTTATATTCATTT
CCCATCAGAGAATTTCAAATCGTAGATCGCTTAATCAGCACTACATTAAAAGATGAA
GTCA-
TGAAGATTATGCCCGTTCAAAAGCAGACCCGTGCTGGTCAGAGGACTCGATTCAAGG
>GZ2VM8F07IE3LK
CGTCTAGTCAAAGAAAACAAGATCAAAACCATCGAAGAAATATATTTATATTCATTT
CCCATCAAAGAATTTCAAATCGTAGATCGCTTAATCGGCACTACATTAAAAGATGAA
GTCA-
TGAAGATTATGCCCGTTCAAAAGCAGACCCGTGCTGGTCAGAGGACTCGATTCAAGG

#ACOB

>GZ2VM8F07ICAGB
TTTGCCGCATTACTATCATCA
>GZ2VM8F07HYJAN
GTTTCCGCATTACTATCATCA

#ARP

>GZ2VM8F07H3FXZ
T-AAATCAGCCGATTGTGAATCTT-G-AACC-TAC-AG-
CTTTGAATATGACTGTAATCGATTTCGATGGTGTAG-CTTACCA-TCTTT-
CAACTCCGGAATCTAAA
>GZ2VM8F07IPOED
T-AAATCAGCCGATTGTGAATCTT-G-AATC-TAC-AG-
CTTTGAATATGACTGTAATCGATTTCGATGGTGTAG-CTTACCA-TCTTT-
CAACTCCGGAATCTAAG
>GZ2VM8F07IJJSG
T-AAATCAGCCGAATGTGAATCTT-G-AACC-TACGAG-
CTTTGAATATGACTGTAATCGATTTCGATGGTGTAG-CTTACCA-TCTTT-
CAACTCCGGAATCTAAA
>GZ2VM8F07H92JT
T-AAATCAGCCGATTGTGAATCTT-G-AACC-TAC-AG-
CTTTGGATATGACTGTAATCGATTTCGATGGTGTAG-CTTACCA-TCTCT-
CAACTCCGGAATCTAAA
>GZ2VM8F07H1HY0
T-AAATTAGCCGATTGTGAATCTT-G-AACC-TAC-AG-
CTTTGAATATGACTGTAATCGATTTCGATGGTGTAG-CTTACCG-TCTTT-
CAACTCCGGAATCTAAA
>GZ2VM8F07IHG99
T-AAATCAGCCGATTGTGAATCTT-G-AACC-TAC-AG-
CTTTGAATATGACTTCAATCGATTTCGATGGTGTAG-CTTACCA-TCTTT-
CAACTCCGGAATCTAAA
>GZ2VM8F07IKCMR

T-AAATCAGCCGATTGTGAATCTT-G-AACC-TAC-AG-
CTTTGAATATGACTGTAATCGATTTCGATGGTGTGG-CTTACCA-TCTTT-
CAACTCCGGAGTCTAAA
>GZ2VM8F07IMLIV
T-GAACCAGCCGATTGTGAATCTT-G-AACC-TAC-AG-
CTTTGGATATGACTGTAGTCGATTACGATGTTGTAG-CTTACCA-TCTTT-
CAACTCCAGAATCTAAA
>GZ2VM8F07IP2FZ
T-AAATCAGCGGATTGTGAATCTT-G-AACC-TAC-AG-
CTTTGAATATGACAGTAATCGATTTCGATGGTGTAG-CTTACCA-TCTTT-
CAACTCCGGAATCTAAA
>GZ2VM8F07H5RR9
T-AAATCAGCCGATTGTGAATCTTCG-AACC-TAC-AG-
CTTTGAATATGACTGTAATCGATTTCGATGGTGTAG-CTTACCA-CCTTT-
CAACTCCGGAATCTAAA
>GZ2VM8F07IIADK
T-GAACCAGCCGATTGTGAATCTT-G-AACC-TAC-AG-
CTTTGAATATGACTGTAATCGATTTCGATGGTGTAG-CTTACCA-TCTTT-
CAACTCCGGAATCTAAA
>GZ2VM8F07H79NW
TAAAATCAGCCGATTGTGAATCTT-G-AACC-TAC-AG-
CTTTGAATATGACTGTAATCGATTTCGATGGTGTAG-CTTATCA-TCTTT-
CAACTCCGGAATCTAAA
>GZ2VM8F07ILRUU
T-AAATCAGCCGATTGTGAATCTT-G-AACCGTAC-AG-
CTTTGAATATGACTGTAATCGATTTCGATGGTGTAG-CTTACCA-TCCTT-
CAACTCCGGAATCTAAA
>GZ2VM8F07H1Q5C
TAAAATCGGCCGATTGTGAATCTT-G-AACC-TAC-AG-
CTTTGAGTATGACTGTAATCGATTTCGATGGTGTAG-CTTACCA-TCTTT-
CAACTCCGGAATCTAAA
>GZ2VM8F07H9EXX
T-AAATCAGCCGATTGTGAATCTT-G-AACC-TAC-AG-
CTTTGGATATGACTGTAATCGATTTCGATGGTGTAG-CTTACCA-TCTTT-
CAACTCCGGAGTCTAAA
>GZ2VM8F07IE4HK
T-AAATCAGCCAATTGTGAATCTT-G-AACC-TAC-AG-
CTTTGAATATGACCGTAATCGATTTCGATGGTGTAG-CTTACCA-TCTTT-
CAACTCCGGAATCTAAA
>GZ2VM8F07INZWY
T-AAATCAGCCGATTGTGAATCTT-G-AGCC-TAC-AG-
CTTTGAATATGACTGTAATCGATTTTGATGGTGTAG-CTTACCA-TCTTT-
CAACTCCGGAATCTAAA

#Ef_tu

>GZ2VM8F07H6U7U

CC-ACTTT-GACAGCTGCAAT-CACCAAATGTTT-AG-CAAGGAAAGGTCAAGCAAAA-
TTCAAAGATTACGGT--GAGATTGATAAA-GCTCCAGAAGAGAAAGCGAGGGG-TAT-
CACCA-T-CG-CTACGGCACACGTAGAATACGAAACTGATGAT-AGACA-
TTATGCACATGTCGATTGTCCTGGCCACGCCGA-TTATA-TT
>GZ2VM8F07IISNC
CC-ACTTT-GACAGCTGCAAT-CACCAAATGTTT-AG-CAAGGAAAGGTCAAGCAAAA-
TTCAAAGGTTACGGT--GAAATTGATAAA-GCTCCAGAAGAGAAAGCGAGGGG-TAT-
CACCA-T-CG-CTACGGCACACGTAGAATACGAAACTGATGAT-AGACA-
TTATGCACATGTCGATTGTCCTGGCCACGCCGA-TTATA-TT
>GZ2VM8F07HYGQH
CC-ACTTT-GACAGTTGCAAT-CACCAAATGTTT-AG-CAAGGAAAGGTCAAGCAAAA-
TTCAAAGATTACGGT--GAAATTGATAAA-GCTCCCGAAGAGAAAGCGAGGGG-TAT-
CACCA-T-CG-CTACGGCACACGTAGAATACGAAACTGATGAT-AGACA-
TTATGCACATGTCGATTGTCCTGGCCACGCCGA-TTATA-TT
>GZ2VM8F07H2GMM
CC-ACTTT-GACAGCTGCAAT-CACCGAATGTTT-AG-CAAGGAAAGGTCAAGCAAAA-
TTCAAAGATTACGGT--GAAATTGATAAA-GCTCCCGAAGAGAAAGCGAGGGG-TAT-
CACCA-T-CG-CTACGGCACACGTAGAATACGAAACTGATGAT-AGACA-
TTATGCACATGTCGATTGTCCTGGCCACGCCGA-TTATA-TT
>GZ2VM8F07H5A4N
CC-ACTTT-GACAGCTGCAAT-CACCAAATGTTT-AG-CAAGGAAAGGTCAAGCAAAA-
TTCAGAGATTACGGT--GAAATTGATAAA-GCTCCCGAAGAGAAAGCGAGGGG-TAT-
CACCA-T-CG-CTACGGCACACGTAGAATACGAAACTGATGAT-AGACA-
TTATGCACATGTCGATTGTCCTGGCCACGCCGA-TTATA-TT
>GZ2VM8F07IF1JW
CC-ACTTT-GACAGCTGCAAT-CACCAAATGTTT-AG-CAAGGAAAGGTCAAGCAAAA-
TTCAAAGATTATGGT--GAAATTGATAAA-GCTCCCGAAGAGAAAGCGAGGGG-TAT-
CACCA-T-CG-CTACGGCACACGTAGAATACGAAACTGATGAT-AGACA-
TTATGCACATGTCGATTGTCCTGGCCACGCCGA-TTATA-TT
>GZ2VM8F07ILDC6
CC-ACTTT-GACAGCTGCAAT-CACCAAATGTTT-AG-CGAGGAAAGGTCAAGCAAAA-
TTCAAAGATTACGGT--GAAATTGATAAA-GCTCCCGAAGAGAAAGCGAGGGG-TAT-
CACCA-T-CG-CTACGGCACACGTAGAATACGAAACTGATGAT-AGACA-
TTATGCACATGTCGATTGTCCTGGCCACGCCGA-TTATA-TT
>GZ2VM8F07IG2EV
CC-ACTTT-GACAGCTGCAAT-CACCAAATGTTT-AG-CAAGGAAAGGTCAAGCAAAA-
TTCAAAGATTACGGT--GAAATTGATAAA-GCTCCAGAAGAGAAAGCGAGGGG-TAC-
CACCA-T-CG-CTACGGCACACGTAGAATACGAAACTGATGAT-AGACA-
TTATGCACATGTCGATTGTCCTGGCCACGCCGA-TTATA-TT
>GZ2VM8F07IC7FI
CC-ACTTT-GACAGCTGCAAT-CACCAAATGTTT-AG-CAAGGAATGGTCAAGCAAAA-
TTCAAAGATTACGGT--GAAATTGATAAA-GCTCCAGAAGAGAAAGCGAGGGG-TAT-
CACCA-T-CG-CTACGGCACACGTAGAATACGAAACTGATGAT-AGACA-
TTATGCACATGTCGATTGTCCTGGCCACGCCGA-TTATA-TT
>GZ2VM8F07IAOMI
CC-ACTTT-GACAGCTGCAAT-CACCAAATGTTT-AG-CAAGGAAAGGTCAAGCGAAA-
TTCAAAGATTACGGT--GAAATTGATAAA-GCTCCAGAAGAGAAAGCGAGGGG-TAT-

CACCA-T-CG-CTACGGCACACGTAGAATACGAAACTGATGAT-AGACA-
TTATGCACATGTCGATTGTCCTGGCCACGCCGA-TTATA-TT
>GZ2VM8F07H0LBX
CC-ACTTT-GACAGCTGCAAT-CGCCAAATGTTT-AG-CAAGGAAAGGTCAAGCAAAA-
TTCAAAGATTACGGT--GAAATTGATAAA-GCTCCAGAAGAGAAAGCGAGGGG-TAT-
CACCA-T-CG-CTACGGCACACGTAGAATACGAAACTGATGAT-AGACA-
TTATGCACATGTCGATTGTCCTGGCCACGCCGA-TTATA-TT
>GZ2VM8F07IGZM9
CC-ACTTT-GACAGCTGCAAT-CACCAAATGTTT-AG-CAAGGAAAGGTCAAGCAAAA-
TTCAAAGATTACGGT--GTAATTGATAAA-GCTCCAGAAGAGAAAGCGAGGGG-TAT-
CACCA-T-CG-CTACGGCACACGTAGAATACGAAACTGATGAT-AGACA-
TTATGCACATGTCGATTGTCCTGGCCACGCCGA-TTATA-TT
>GZ2VM8F07H43O3
CC-ACTTT-GACAGCTGCAAT-CACCAAATGTTT-AG-CAAGGAAAGGTCAAGCAAAA-
TTCAAAGATTACGGT--GAAATTGATAAA-GCTCCCGTAGAGAAAGCGAGGGG-TAT-
CACCA-T-CG-CTACGGCACACGTAGAATACGAAACTGATGAT-AGACA-
TTATGCACATGTCGATTGTCCTGGCCACGCCGA-TTATA-TT
>GZ2VM8F07IK1XG
CC-ACTTT-GACAGCTGCAAT-CACCAAATGTTT-AG-CAAGGAAAGGTCAAGCAAAA-
TTCAAAGATTACGGT--GAAATTGATAAA-GCTCCCGAAGAGAAAGCGAGGGG-TAT-
CACCA-T-CG-CTACGGCACACGTAGAATACGAAACTGATGAT-AGACA-
TTGTGCACATGTCGATTGTCCTGGCCACGCCGA-TTATA-TT
>GZ2VM8F07IJQAX
CC-ACTTT-GACAGCTACAAT-CACCAAATGTTT-AG-CAAGGAAAGGTCAAGCAAAA-
TTCAAAGATTACGGT--GAAATTGATAAA-GCTCCAGAAGAGAAAGCGAGGGG-TAT-
CACCA-T-CG-CTACGGCACACGTAGAATACGAAACTGATGAT-AGACA-
TTATGCACATGTCGATTGTCCTGGCCACGCCGA-TTATA-TT
>GZ2VM8F07IIPST
CC-ACTTT-GACAGCTGCAAT-CACCAAATGCTT-AG-CAAGGAAAGGTCAAGCAAAA-
TTCAAAGATTACGGT--GAAATTGATAAA-GCTCCAGAAGAGAAAGCGAGGGG-TAT-
CACCA-T-CG-CTACGGCACACGTAGAATACGAAACTGATGAT-AGACA-
TTATGCACATGTCGATTGTCCTGGCCACGCCGA-TTATA-TT
>GZ2VM8F07HZ4AY
CC-ACTTT-GACAGCTGCAAT-CACCAAATGTTT-AG-CAAGGAAAGGTCAAGCAAAA-
TTCAAAGATTACGGT--GAAATCGATAAA-GCTCCCGAAGAGAAAGCGAGGGG-TAT-
CACCA-T-CG-CTACGGCACACGTAGAATACGAAACTGATGAT-AGACA-
TTATGCACATGTCGATTGTCCTGGCCACGCCGA-TTATA-TT
>GZ2VM8F07ID2EQ
CC-ACTTT-GACAGCTGCAAT-CACCAAATGTTT-AG-CAAGGAAAGGTCAAGCAAAA-
TTCAAAGATTACGGT--GAAGTTGATAAA-GCTCCAGAAGAGAAAGCGAGGGG-TAT-
CACCA-T-CG-CTACGGCACACGTAGAATACGAAACTGATGAT-AGACA-
TTATGCACATGTCGATTGTCCTGGCCACGCCGA-TTATA-CT
>GZ2VM8F07IIGL1
CC-ACTTT-GACAGCTGCAAT-CACCAAATGTTT-AG-CAAGGAAAGGTCAAGCAAAA-
TTCAAAGATTACGGT--GAAATTGATAAA-GCTCCCGAAGAGAAAGCGAGGGG-TAT-
CACCA-T-CG-CTACGGCACACGTAGAATACGAAACTGATGAT-AGACA-
TTATGCACGTGTCGATTGTCCTGGCCACGCCGA-TTATA-TT

>GZ2VM8F07H2T66
CC-ACTTT-GACAGCTGCAAT-CACCAAATGTTT-AG-CAAGGAAAGGTCAAGCAAAA-
TTCAAAGATTACGGT--GAAATTGATAAA-GCTCCCGAAGAGAAAGCGAGGGG-TAT-
CACCA-T-CG-CTACGGCACACGTAGAATACGAAACTGATGAT-AGACA-
TTATGCACATGTCGGTTGTCCTGGCCACGCCGA-TTATA-TT
>GZ2VM8F07IIZ0O
CC-ACTTT-GACAGCTGCAAT-CACCAAATGTTT-AG-CAAGGAAAGGTCAAGCAAAA-
TTCAAAGATTACGGT--GAAATTGGTAAA-GCTCCCGAAGAGAAAGCGAGGGG-TAT-
CACCA-T-CG-CTACGGCACACGTAGAATACGAAACTGATGAT-AGACA-
TTATGCACATGTCGATTGTCCTGGCCACGCCGA-TTATA-TT
>GZ2VM8F07IM0W3
CC-ACTTT-GACAGCTGCAAT-CACCAAATGTTT-AG-CAAGGAAAGGTCAAGCAAAA-
TTCAAAGATTACGGT--GAAATTGATAAA-GCTCCCGAAGAGAAAGCGAGGGG-TAT-
CACCA-T-CG-CTACGGCACACGTAGAATACGAAACTGGTGAT-AGACA-
TTATGCACATGTTGATTGTCCTGGCCACGCCGA-TTATA-TT
>GZ2VM8F07IBMUP
CC-ACTTT-GACAGCTGCAAT-CACCAAATGTTT-AG-CAAGGAAAGGTCAAGCAAAA-
TTCAAAGATTACGGT--GAAATTGATAAA-GCTCCAGAAGAGAAAGCGAGGGG-TAT-
CACCA-T-CG-CTACGGCACACGTAGAATACGAAACTGATGAT-AGACA-
TTATGCACATGTCGATTGCCCTGGCCACGCCGA-TTATA-TT
>GZ2VM8F07ICFU0
CC-ACTTT-GACAGCTGCAAT-CACCAAATGTTT-AG-CAAGGAAAGGTCAAGCAAAA-
TTCAAAGATTACGGT--GAAATTGATAAA-GCTCCAGAAGAGAAAGCGAGGGG-TAT-
CACCA-T-CG-CTACGGCACACGTAGAATACGAAACTGATGAT-AGACA-
TTATGCACATGTCGATTGTCCTGGCCACGCCGA-TTAAT-AT
>GZ2VM8F07IP82Q
CC-ACTTT-GACAGCTGCAAT-CACCAAATGTTT-AG-CAAGGAAAGGTCAAGCAAAA-
TTCAAAGATTACGGT--GAAATTGATAAA-GCTCCAGAAGAGAAAGCGAGGGG-TAT-
CACCA-T-CG-CTACGGCACACGTAGAATACAAAACTGATGAT-AGACA-
TTATGCACATGTCGATTGTCCTGGCCACGCCGA-TTATA-TT
>GZ2VM8F07IF53N
CC-ACTTT-GACAGCTGCAAT-CACCAAATGTTT-AG-CAAGGAAAGGTCAAGCAAAA-
TTCAAAGATTACGGT--GAAATTGATAAA-GCTCCAGAAGAGAAAGCGAGGGG-TAT-
CACCAAT-CG-CTACGGCACACGTAGAATACGAGACTGATGAT-AGACA-
TTATGCACATGTCGATTGTCCTGGCCACGCCGA-TTATA-TT
>GZ2VM8F07H7EE2
CC-ACTTT-GACAGCTGCAAT-CACCAAATGTTT-AG-CAAGGAAAGGTCAAGCAAAA-
TTCAAAGATTACGGT--GAAGTTGATAAA-GCTCCCGAAGAGAAAGCGAGGGG-TAT-
CACCA-T-CG-CTACGGCACACGTAGAATACGAAACTGATGAT-AGACA-
TTATGCACATGTCGATTGTCCTGGCCACGCCGA-TTATA-TT
>GZ2VM8F07IG8SS
CC-ACTTT-GACAGCTGCAAT-CACCAAATGTTT-AG-CAAGGAAAGGTCAAGCAAAA-
TTCAAAGATTACGGT--GAAATTGATAAA-GCTCCCGAAGAGAAAGCGAGGGG-TAT-
CACCA-T-CG-CTACGGCACACGTAGAATACGAAGCTGATGAT-AGACA-
TTATGCACATGTCGATTGTCCTGGCCACGCCGA-TTATA-TT
>GZ2VM8F07H16NV

CC-ACTTT-GACAGCTGCAAT-CACCAAATGTTT-AG-CAAGGAAAGGTCAAGCAAAA-
TTCAAAGATTACGGT--GAAATTGATAAA-GCTCCCGAAGAGAAAGCGAGGGG-TAT-
CACCA-T-CG-CTACGGCACACGTAGAATACGAGACTGATGAT-AGACA-
TTATGCACATGTCGATTGTCCTGGCCACGCCGA-TTATA-TT
>GZ2VM8F07ICAJN
CC-ACTTT-GACAGCTGCAAT-CACCAAATGTTT-AG-CAAGGAGAGGTCAAGCAAAA-
TTCAAAGATTGCGGT--GAAATTGATAAA-GCTCCCGAAGAGAAAGCTAGGGG-TAT-
CACCA-T-CG-CTACGGCACACGTAGAATACGAAACTGATGAT-AGACA-
TTATGCACATGTCGATTGTCCTGGCCACGCCGA-TTATA-TT
>GZ2VM8F07IEJN4
CC-ACTTT-GACAGCTGCAAT-CACCAAATGTTT-GG-CAAGGAAAGGTCAAGCAAAA-
TTCAAAGATTACGGT--GAAATTGATAAA-GCTCCCGAAGAGAAAGCG-GGGG-TAT-
CACCA-T-CG-CTACGGCGCACGTAGAATACGAAACTGATGAT-AGACA-
TTATGCACATGTCGATTGTCCTGGCCACGCCGA-TTATA-TT
>GZ2VM8F07IQ5DC
CC-ACTTT-GACAGCTGCAAT-CACCAAATGTTT-AG-CAAGGAAAGGTCAAGCAAAA-
TTCAAAGATTACGGT--GAAATTGATAAA-GCTCCCGAAGAGAAAGCGAGGGG-TAT-
CACCA-T-CG-CTACGGCACTCGTAGAATACGAAACTGATGAT-AGACA-
TTATGCACATGTCGATTGTCCTGGCCACGCCGA-TTATA-TT
>GZ2VM8F07ILN2K
CC-ACTTT-GACAGCTGCAAT-CACCAAATGTTT-AG-CAAGGAAAGGTCAAGCAAAA-
TTCAAAGATTACGGT--GAAATTGATAAA-GCTCCCGAAGAGAAGGCGAGGGG-TAT-
CACCA-T-CG-CTACGGCACACGTAGAATACGAAACTGATGAT-AGACA-
TTATGCACATGTCGATTGTCCTGGCCACGCCGA-TTATA-TT
>GZ2VM8F07IFZ26
CC-ACTTT-GACAGCTGCAAT-CACCAAATGTTT-AG-CAAGGAAAGGTCAAGCAAAA-
TTCAAAGATTACGGT--GAAATTGATAAA-GCTCCCGAAGAGAAAGCGAGGGG-TAT-
CACCA-T-CG-CTACGGCACACGTAGAGTACGAAACTGATGAT-AGACA-
TTATGCACATGTCGATTGTCCTGGCCACGCCGA-TTATA-TT
>GZ2VM8F07IEZ4K
CC-ACTTT-GACAGCTGCAAT-CACCAAATGTTT-AG-CAAGGAAAGGTCAAGCAATA-
TTCAAAGATTACGGT--GAAATTGATAAA-GCTCCCGAAGAGAAAGCGAGGGG-TAT-
CACCA-T-CG-CTACGGCACACGTAGAATACGAAACTGATGAT-AGACA-
TTATGCACATGTCGATTGTCCTGGCCACGCCGA-TTATA-TT
>GZ2VM8F07IBORG
CC-ACTTT-GACAGCTGCAAT-CACCAAATGTTT-AG-CAAGGAAAGGTCAAGCAAAA-
TTCAAAGATTGCGGT--GAAATTGATAAA-GCTCCCGAAGAGAAAGCGAGGGG-TAT-
CACCA-T-CG-CTACGGCACACGTAGAATACGAAACTGATGAT-AGACA-
TTATGCACATGTCGATTGTCCTGGCCACGCCGA-TTATA-TT
>GZ2VM8F07IJ6TY
CC-ACTTT-GACAGCTGCAAT-CACCAAATGTTT-AG-CAAGGAAAGGTCAAGCAAAA-
TTCAAAGATTACGGT--GAAATTGATGAA-GCTCCCGAAGAGAAAGCGAGGGG-TAT-
CACCA-T-CG-CTACGGCACACGTAGAATACGAAACTGATGAT-AGACA-
TTATGCACATGTCGATTGTCCTGGCCACGCCGA-TTATA-TT
>GZ2VM8F07ICWRF
CC-ACTTT-GACAGCTGCAAT-CACCAAATGTTT-AG-CAAGGAAAGGTCAAGCAAAA-
TTCAAAGATTACGGT--GAAATTGATAAA-GCTCCAGAAGAGAAAGCGAGGGG-TAT-

CACCA-T-CG-CTACGGCACACGTAGAATACGAAACTGATGAT-AGACA-
TTACGCACATGTCGATTGTCCTGGCCACGCCGA-TTATA-TT
>GZ2VM8F07H4LX2
CC-ACTTT-GACAGCTGCAAT-CACCAAATGTTT-AG-CAAGGAAAGGTCAAGCAAAA-
TTCAAAGATTACGGT--GAAATTGATAAA-GCTCCCGAAGAGAAAGCGAGGGG-TGT-
CACCA-T-CG-CTACGGCACACGTAGAATACGAAACTGATGAT-AGACA-
TTATGCACATGTCGATTGTCCTGGCCACGCCGA-TTATA-TT
>GZ2VM8F07H1I92
CC-ACTTT-GACAGCTGCAAT-CACCAAATGTTT-AG-CAGGGAAAGGTCAAGCAAAA-
TTCAAAGATTACGGT--GAAATTGATAAA-GCTCCAGAAGAGAAAGCGAGGGG-TAT-
CACCA-T-CG-CTACGGCACACGTAGAATACGAAACTGATGAT-AGACA-
TTATGCACATGTCGATTGTCCTGGCCACGCCGA-TTATA-TT
>GZ2VM8F07IGMAI
CC-ACTTT-GACAGCTGCAAT-CACCAAATGTTT-AG-CAAGGAAAGGTCAAGCAAAA-
TTCAAAGATTACGGT--GAAATTGATAAA-GCTCCAGAGGAGAAAGCGAGGGG-TAT-
CACCA-T-CG-CTACGGCACACGTAGAATACGAAACTGATGAT-AGACA-
TTATGCACATGTCGATTGTCCTGGCCACGCCGA-TTATA-TT
>GZ2VM8F07II3BY
CC-ACTTT-GACAGCTGCAAT-CACCAAATGTTT-AG-CAAGGAAAGGTCAAGCAAAA-
TTCAAAGATTACAGT--GAAATTGATAAA-GCTCCCGAAGAGAAAGCGAGGGG-TAT-
CACCA-T-CG-CTACGGCACACGTAGAATACGAAACTGATGAT-AGACA-
TTATGCACATGTCGATTGTCCTGGCCACGCCGA-TTATA-TT
>GZ2VM8F07H6LFW
CC-ACTTT-GACAGCTGCAAT-CACCAAATGTTT-AG-CAAGGAAAGGTCAAGCAAAA-
TCCAAAGATTACGGT--GAAATTGATAAA-GCTCCCGAAGAGAAAGCGAGGGG-TAT-
CACCA-T-CG-CTACGGCACACGTAGAATACGAAACTGATGAT-AGACA-
TTATGCACATGTCGATTGTCCTGGCCACGCCGA-TTATA-TT
>GZ2VM8F07H4135
CC-ACTTT-GACAGCTGCAAT-CACCAAATGTTT-AG-CAAGGAAAGGTCGAGCAAAA-
TTCAAAGATTACGGT--GAAATTGATAAA-GCTCCAGAAGAGAAAGCGAGGGG-TAT-
CACCA-T-CG-CTACGGCACACGTAGAATACGAAACTGATGAT-AGACA-
TTATGCACATGTCGATTGTCCTGGCCACGCCGA-TTATA-TT
>GZ2VM8F07H4TLX
CC-ACTTT-GACAGCTGCAAT-CACCAAATGTTT-AG-CAAGGAAAGGTCAAGCAAAA-
TTCAAAGATTACGGT--GAAATTGATAAA-GCTCCAGAAGAGAAAGCGAGGGG-TAT-
CACCA-T-CG-CTACGGCACACGTAGAATACGAAACTGATGAT-AGACA-
TTATGCACATGTCGACTGTCCTGGCCACGCCGA-TTATA-TT
>GZ2VM8F07HX9NA
CC-ACTTT-GACAGCTGCAAT-CACCAAATGTTT-AG-CAAGGAAAGGTCATGCAAAA-
TTCAAAGATTACGGT--GAAATTGATAAA-GCTCCCGAAGAGAAAGCGAGGGG-TAT-
CACCA-T-CG-CTACGGCACACGTAGAATACGAAACTGATGAT-AGACA-
TTATGCACATGTCGATTGTCCTGGCCATGCCGA-TTATA-TT
>GZ2VM8F07HZ6RE
CC-ACTTT-GACAGCTGCAAT-CACCAAATGTTT-AG-CAAGGAAAGGTCAAGCAAAA-
TTCAAAGATTACGGT--GAAATTGATAAA-GCTCCCGAAGAGAAAGCGAGGGG-TAT-
CACCA-TACG-CTACGGCACACGTAGAATACGAAACTGATGAT-AGACA-
TTATGCACATGTCGATTGTCCTGGCCACGCCGA-TTATA-TT

>GZ2VM8F07H4667
CC-ACTTT-GACAGCTGCAAT-CACCAAATGTTT-AG-CAAGGAAAGGTCAAGCAAAA-
TTCAAAGATTACGGT--GAAATTGATAAA-GCTCCCGAAGAGAAAGCGAGGGG-TAT-
CACCA-T-CG-CTACGGCACACGTAGAATACGAAACTGATGAT-AGACA-
TTATGCACATGTCGATTGTCCTGGCCACGCCGA-CTATA-TT
>GZ2VM8F07IJHAX
CC-ACTTT-GACAGCTGCAAT-CACCAAATGTTT-AG-CAAGGAAAGGTCAAGCAAAA-
TTCAAAGATTACGGT--GAAATTGATAAA-GCTCCCGAAGAGAAAGCGAGGGG-TAT-
CGCCA-T-CG-CTACGGCACACGTAGAATACGAAACTGATGAT-AGACA-
TTATGCACATGTCGATTGTCCTGGCCACGCCGA-TTATA-TT
>GZ2VM8F07ILK2W
CC-ACTTT-GACAGCTGCAAT-CACCAAATGTTT-AG-CAAGGAAAGGTCAAGCAAAA-
TTCAAAGATTACGGT--GAAATTGATAAA-GCTCCAGAAGGGAAAGCGAGGGG-TAT-
CACCA-T-CG-CTACGGCACACGTAGAATACGAAACTGATGAT-AGACA-
TTATGCACATGTCGATTGTCCTGGCCACGCCGA-TTATA-TT
>GZ2VM8F07H8ZR7
CC-ACTTT-GACAGCTGCAAT-CACCAAATGTTT-AG-CAAGGAAAGGTCAAGCAAAA-
TTCAAAGATTACGGT--GAAATTGATAAA-GCTCCCGAAGAGAAAGCGAAGGG-TAT-
CACCA-T-CG-CTACGGCACACGTAGAATACGAAACTGATGAT-AGACA-
TTATGCACATGTCGATTGTCCTGGCCACGCCGA-TTATA-TT
>GZ2VM8F07H2PZF
CC-ACTTT-GACAGCTGCGAT-CACCAAATGTTT-AG-CAAGGAAAGGTCAAGCAAAA-
TTCAAAGATTACGGT--GAAATTGATAAA-GCTCCCGAAGAGAAAGCGAGGGG-TAT-
CACCA-T-CG-CTACGGCACACGTAGAATACGAAACTGATGAT-AGACA-
TTATGCACATGTCGATTGTCCTGGCCACGCCGA-TTATA-TT
>GZ2VM8F07IC7Q9
CC-ACTTT-GACAGCTGCAAT-CACCAAATGTTT-AG-CAAGGAAAGGTCAAGCAAAA-
TTCAAAGATTACGGT--GAAATTGATAAA-GCTCCCGAAGAGAAAGCGAGGGG-TAT-
CACCA-T-CG-CTACGGCACACGTAGAATACGAAACTGATGAT-AGACA-
TTATGCACATGTCGATTGTCCGGGCCACGCCGA-TTATA-TT
>GZ2VM8F07H0VHY
CC-ACTTT-GACAGCTGCAAT-CACCAAATGTTT-AG-CAAGGAAAGGTCAAGCAAAA-
TTCAAAGATTACGGT--GAAATTGAT-AA-GCTCCCGAAGAGAAAGCGAGGGG-TAT-
CACCA-T-CG-CTACGGCACACGTAGAATACGAAACTGATGAT-AGACA-
TTATGCACATGTCGATTGTCCTGGCCACGCCGA-TTACA-TT
>GZ2VM8F07IP4WS
CC-ACTTT-GACAGCTGCAAT-CACCAAATGTTT-AA-CAAGGAAAGGTCAAGCAAAA-
TTCAAAGATTACGGT--GAAATTGATAAA-GCTCCCGAAGAGAAAGCGAGGGG-TAT-
CACCA-T-CG-CTACGGCACACGTAGAATACGAAACTGATGAT-AGACA-
TTATGCACATGTCGATTGTCCTGGCCACGCCGA-TTATA-TT
>GZ2VM8F07H2XQJ
CC-ACTTT-GACAGCTGCAAT-CACCAAATGTTT-AG-CAAGAAAAGGTCAAGCAAAA-
TTCAAAGATTACGGT--GAAATTGATAAA-GCTCCCGAAGAGAAAGCGAGGGG-TAT-
CACCA-T-CG-CTACGGCACACGTAGAATACGAAACTGATGAT-AGACA-
TTATGCACATGTCGATTGTCCTGGCCACGCCGA-TTATA-TT
>GZ2VM8F07ICGH2

CC-ACTTT-GACAGCTGCAAT-CACCAAATGTTT-AG-CAAGGAAAGGTCAAGCAAAA-
TTCAAAGATTACGGT--GAAATTGATAAG-GCTCCCGAAGAGAAAGCG-GGGG-TAT-
CACCA-T-CG-CTACGGCACACGTAGAATACGAAACTGATGAT-AGACA-
TTATGCACATGTCGATTGTCCTGGCCACGCCGA-TTATA-TT
>GZ2VM8F07H9IE7
CC-ACTTT-GACAGCTGCAAT-CACCAAATGTTT-AG-CAAGGAAAGGTCAAGCAAAA-
TTCAAAGATTACGGT--GAAATTGATAAG-GCTCCAGAAGAGAAAGCGAGGGG-TAT-
CACCA-T-CG-CTACGGCACACGTAGAATACGAAACTGATGAT-AGACA-
TTATGCACATGTCGATTGTCCTGGCCACGCCGA-TTATA-TT
>GZ2VM8F07ILV3Q
CC-ACTTT-GACAGCTGCAAT-CACCAAATGTTT-AG-CAAGGAAAGGTCGAGCAAAA-
TTCAAAGATTACGAT--GAAATTGATAAA-GCTCCCGAAGAGAAAGCGAGGGG-TAT-
CACCA-T-CG-CTACGGCACACGTAGAATACGAAACTGATGAT-AGACA-
TTATGCACATGTCGATTGTCCTGGCCACGCCGA-TTATA-TT
>GZ2VM8F07ILDFA
CC-ACTTT-GACAGCTGCAAT-CACCAAATGTTT-GG-CAAGGAAAGGTCAAGCAAAA-
TTCAAAGATTACGGT--GAAATTGATAAA-GCTCCAGAAGAGAAAGCGAGGGG-TAT-
CACCA-T-CG-CTACGGCACACGTAGAATACGAAACTGATGAT-AGACA-
TTATGCACATGTCGATTGTCCTGGCCACGCCGA-TTATA-TT
>GZ2VM8F07IAC7Y
CC-ACTTT-GACAGCTGCAAT-CACCAAATGTTT-AG-CAAGGAAAGGTCAAGCAAAA-
TTCGAAGATTACGGT--GAAATTGATAAA-GCTCCCGAAGAGAAAGCGAGGGG-TAT-
CACCA-T-CG-CTACGGCACACGTAGAATACGAAACTGATGAT-AGACA-
TTATGCACATGTCGATTGCCCT-GCCACGCCGA-TTATA-TT
>GZ2VM8F07IMRPC
CC-ACTTT-GACAGCTGCAAT-CACCAAGTGTTT-AG-CAAGGAAAGGTCAAGCAAAA-
TTCAAAGATTACGGT--GAAATTGATAAA-GCTCCAGAAGAGAAAGCGAGGGG-TAT-
CACCA-T-CG-CTACGGCGCACGTAGAATACGAAACTGATGAT-AGACA-
TTATGCACATGTCGATTGTCCTGGCCACGCCGA-TTATA-TT
>GZ2VM8F07IJX62
CC-ACTTT-GACAGCTGCAAT-CACCAAATGTTT-AG-CAAGGAAAGGTCAAGCAAAA-
TTCAAAGATTACGGT--GAAATTGATAAA-GCTCCAGAAGAGAAAGCGAGGGG-CAT-
CACCA-T-CG-CTACGGCACACGTAGAATACGAAACTGATGAT-GGACA-
TTATGCACATGTCGATTGTCCTGGCCACGCCGA-TTATA-TT
>GZ2VM8F07IE5UG
CC-ACTTT-GACAGCTGCAAT-CACCAAATGTTT-AG-CAAGGAAAGGTCAAGCAAAA-
TTCAAAGATTACGGT--GAAATTGATAAA-GCTCCAGAAGAGAAAGCGAGGGG-TAT-
CACCA-T-CG-CTATGGCACACGTAGAATACGAAACTGATGAT-AGACA-
TTATGCACATGTCGATTGTCCTGGCCACGCCGA-TTATA-TT
>GZ2VM8F07IOAKQ
CC-ACTTT-GACAGCTGCAAT-CACCAAATGTTT-AG-CAAGGAAAGGTCAAGCAAAA-
TTCAAAGATTACGGT--GAAATTGATAAA-GCTCCAGAAGAGAAAGCGAGGGG-TAT-
CACCA-T-CG-CTACGGCACACGTAGAATACGAAACTGATGAT-AGACA-
TTATGCACATGTCGATTGTCCTGGCCACGCCGA-TTATA-AT
>GZ2VM8F07H86GK
CC-ACTTT-GACAGCTGCAAT-CACCAAATGTTT-AG-CAAGGAAAGGTCAAGCAAGA-
TTCAAAGATTACGGT--GAAATTGATAAA-GCTCCAGAAGAGAAAGCGAGGGG-TGT-

CACCA-T-CG-CTACGGCACACGTAGAATACGAAACTGATGAT-AGACA-
TTATGCACATGTCGATTGTCCTGGCCACGCCGA-TTATA-TT
>GZ2VM8F07H0AHD
CC-ACTTT-GACAGCTGCAAT-CACCAAATGTTT-AG-CAAGGAAGGTCAAGCAAAA-
TTCAAAGATTACGGT--GAAATTGATAAA-GCTCCAGAAGAGAAAGCGAGGGG-TAT-
CACCA-T-CG-CTACGGCACACGTAGAATACGAAACTGATGAT-AGACA-
TTATGCACATGTCGATTGTCCTGGCCACGCCGA-TTATA-TT
>GZ2VM8F07H084K
CC-ACTTT-GACAGCTGCAAT-CACCAAATGTTT-AG-CAAGGAAAGGTCAAGCAAAA-
TTCAAAGATTACGGT--GAAATTGATAAA-GCTCCCGAAGAGAAAGCGAGGGG-TAT-
CACCA-T-CG-CTACGGCACACGTAGAATGCGAAACTGATGAT-AGACA-
TTATGCACATGTCGATTGTCCTGGCCACGCCGA-TTATA-TT
>GZ2VM8F07II7Q7
CC-ACTTT-GACAGCTGCAAT-CACCAAATGTTT-AG-CAAGGAAAGGTCAAGCAAAA-
TTCAAAGATTACGGT--GAAATTGATAAA-GCTCCCGAAGAGAAAGCGAGGGG-TAT-
CACCA-T-CG-CTACGGCACACGTAGAATACGAAACTGATGAT-AGACG-
TTATGCACATGTCGATTGTCCTGGCCACGCCGA-TTATA-TT
>GZ2VM8F07H3K13
CC-ACTTT-GACAGCTGCAAT-CACCAAATGTTT-AG-CAAGGAAAGGTCAAGCAAAA-
TTCAAAGATTACGGT--GAAATTGATAAA-GCTCCCGAAGAGAAAGCGAGGGG-TAT-
CATCA-T-CG-CTACGGCACACGTAGAATACGAAACTGATGAT-AGACA-
TTATGCACATGTCGATTGTCCTGGCCACGCCGA-TTATA-TT
>GZ2VM8F07IICD6
CC-ACTTT-GACAGCTGCAAT-CACCAAATGTTT-AG-CAAGGAAGGGTCAAGCAAAA-
TTCAAAGATTACGGT--GAAATTGATAAA-GCTCCCGAAGAGAAAGCGAGGGG-TAT-
CACCA-T-CG-CTACGGCACACGTAGAATACGAAACTGATGAT-AGACA-
TTATGCACATGTCGATTGTCCTGGCCACGCCGA-TTATA-TT
>GZ2VM8F07HX3OT
CC-ACTTT-GACAGCTGCAAT-CATCAAATGTTT-AG-CAAGGAAAGGTCAAGCAAAA-
TTCAAAGATTACGGT--GAAATTGATAAA-GCTCCCGAAGAGAAAGCGAGGGG-TAT-
CACCA-T-CG-CTACGGCACACGTAGAATACGAAACTGATGAT-AGACA-
TTATGCACATGTCGATTGTCCTGGCCACGCCGA-TTATA-TT
>GZ2VM8F07H4LC8
CC-ACTTT-GACAGCTGCAAT-CACCAAATGTTT-AG-CAAGGAAAGGTCAAGCAAAA-
TTCAAAGATTACGGT--GAAATTGATAAA-GCTCCCGAAGAGAAAGCGAGGGG-TAT-
CACCA-T-CG-CTACGGCACACGTAGAATACGAAACTGATGAT-AGACA-
TTATGCGCATGTCGATTGTCCTGGCCACGCCGA-TTATA-TT
>GZ2VM8F07HYWQJ
CC-ACTTT-GACAGCTGCAAT-CACCAAATGTTT-AG-CAAGGAGAGGTCAAGCAAAA-
TTCAAAGATTACGGT--GAAATTGATAAA-GCTCCAGAAGAGAAGGCGAGGGG-TAT-
CACCA-T-CG-CTACGGCACACGTAGAATACGAAACTGATGAT-AGACA-
TTATGCACATGTCGATTGTCCTGGCCACGCCGA-TTATA-TT
>GZ2VM8F07IJJ55
CC-ACTTT-GACTGCTGCAAT-CACCAAATGTTT-AG-CAAGGAAAGGTCAAGCAAAA-
TTCAAAGATTACGGT--GAAATTGATAAA-GCTCCAGAAGAGAAAGCGAGGGG-TAT-
CACCA-T-CG-CTACGGCACACGTAGAATACGAAACTGATGAT-AGACA-
TTATGCACATGTCGATTGTCCTGGCCACGCCGA-TTATA-TT

>GZ2VM8F07HZY8Q
CC-ACTTT-GACAGCTGCAAT-CACCAAATGTTT-AG-CAAGGAAAGGTCAAGCAAAA-
TTCAAAGATTACGGT--GAAATTGATAAA-GCTCCAGAAGAGAAAGCGAGGGG-TAT-
CACCA-T-CG-CTACGGCACACGTAGAATACGAAACTGATGAT-AGACA-
TTATGCTCATGTCGATTGTCCTGGCCACGCCGA-TTATA-TT
>GZ2VM8F07IQ0WC
CC-ACTTT-GACAGCTGCAAT-CACCAAATGTTT-AG-CAAGGAAAGGTCAAGCAAAA-
TTCAAAGATTACGGT--GAAATTGATAAA-GCTCCCGAAGAGAAAGCGAGGGG-TAT-
CACCA-T-CG-CTACGGCACACGTAGAATACGAAACTGATGAT-AGACA-
TTATGCACATGTCGATTGTCCTGGCCACGCCGA-TTATAATT
>GZ2VM8F07IPYI6
CC-ACTTT-GACAGCTGCAAT-CACCAAATGTTT-AG-CAAGGAAAGGTCAAGCAAAA-
TTCAAAGATTACGGT--GAAATTGATAAA-GCTCCCGAAGAGAAAGCGAGGGG-TAT-
CACCA-T-CG-CTACGGCACACGTGGAATACGAAACTGATGAT-AGACA-
TTATGCACATGTCGATTGTCCTGGCCACGCCGA-TTATA-TT
>GZ2VM8F07IF62S
CC-ACTTT-GACAGCTGCAAT-CACCAAATGTTT-AG-CAAGGAAAGGTCAAGCAAAA-
TTCAAAGATTACGGT--GAAATTGATAAA-GCTCCAGAAGAGAAAGCGAGGGG-TAT-
CACCA-T-CG-CTACGGCACACGTAAAATACGAAACTGATGAT-AGACA-
TTATGCACATGTCGATTGTCCTGGCCACGCCGA-TTATA-TT
>GZ2VM8F07H35FH
CC-ACTTT-GACAGCTGCAAT-CACCAAATGTTT-AG-CAAGGAAAGGTCAAGCAAAA-
TTCAAAGATTACGGT--GAAATTGATAAA-GCTCCAGAAGAGAAAGCGAGGGG-TAT-
CACCA-T-CG-CTACGGCACACGTAGAATACGAAACTGATGAT-AGACA-
CTATGCACATGTCGATTGTCCTGGCCACGCCGA-TTATA-TT
>GZ2VM8F07IG2S6
CC-ACTTT-GACAGCTGCAAT-CACCAAATGTTT-AG-CAAGGAAAGGTCAAGCAAAA-
TTCAAAGATTACGGT--GAAATTGATAAA-GCTCCAGAAGAGAAAGCGAGGGG-TAT-
CACCA-T-CG-CTACGGCACATGTAGAATACGAAACTGATGAT-AGACA-
TTATGCACATGTCGATTGTCCTGGCCACGCCGA-TTATA-TT
>GZ2VM8F07IQAKO
CC-ACTTT-GACAGCTGCAAT-CACCAAATGTTT-AG-CAAGG-AAGGTCAAGCAAAA-
TTCAAAGATTACGGT--GAAATTGATAAA-GCTCCCGAAGAGAAAGCGAGGGG-TAT-
CACCA-T-CG-CTACGGCACACGTAGAATACGAAACTGATGAT-AGACA-
TTATGCACATGTCGATTGTCCTGGCCACGCCGA-TTATA-TT
>GZ2VM8F07H3B5X
CC-ACTTT-GACAGCTGCAAT-CACCAAATGTTT-AG-CAAGGAAAGGTCAAGCAAAA-
TTCAAAGATTACGGT--GAAATTGATAAA-GCTCCAGAAGAGAAAGCGAGGGG-TAT-
CACCA-T-CG-CTACGGCACACGTAGAATACGAAACTGATGAT-AGACA-
TTATGCACATGTCGATTGTCCTGGCCACGTCGA-TTATA-TT
>GZ2VM8F07INO0L
CC-ACTTT-GACAGCTGCAAT-CACCAAATGTTT-AG-CAAGGAAAGGTCAAGCAAAA-
CTCAAAGATTACGGT--GAAATTGATAAA-GCTCCCGAAGAGAAAGCGAGGGG-TAT-
CACCA-T-CG-CTACGGCACACGAAGAATACGAAACTGATGAT-AGACA-
TTATGCACATGTCGATTGTCCTGGCCACGCCGA-TTATA-CT
>GZ2VM8F07IQ2IR

CC-ACTTT-GACAGCTGCAAT-CACCAAATGTTT-AG-CAAGGAAAGGTCAAGCAAAA-TTCAAAGATTACGGT--GAAATTGATAAA-GCTCCAGAAGAGAAAGCGAGGGG-TAT-CACCA-C-CG-CTACGGCACACGTAGAATACGAAACTGATGAC-AGACA-TTATGCACATGTCGATTGTCCTGGCCACGCCGA-TTATA-TT
>GZ2VM8F07IG7XX
CC-ACTTT-GACAGCTGCAAT-CACCAAATGTTT-AG-CAAGGAAAGGTCAAGCAAAA-TTCAAAGATTACGGT--GAAATTGATAAA-GCTCCAGAAGAGAAAGCGAGGGG-TAT-CACCG-T-CG-CTACGGCACACGTAGAATACGAAACTGATGAT-AGACA-TTATGCACATGTCGATTGTCCTGGCCACGCCGA-TTATA-TT
>GZ2VM8F07IHJFR
CC-ACTTT-GACAGCTGCAGT-CACCAAATGTTT-AG-CAAGGAAAGGTAAAGCAGAA-TTCAAAGATTACGGT--GAAATTGATAAA-GCTCCCGAAGAGAAAGCGTGGGG-TAT-CACCA-T-CGACTACGGCACACGTAGAATACGAAACTGATGAT-AGACA-TTATGCACATGTCGATTGTCCTGGCCACGCCGA-TTATA-TT
>GZ2VM8F07H2QLF
CC-ACTTT-GACAGCTGCAAT-CACCAAATGTTT-AG-CAAGGAAAGGTCAAGCAAAA-TTCAAAGATTACGGT--GAAATTGATAAA-GCTCCCGAAGAGAAAGCGAGGGG-TATCAACCA-T-CG-CTACGGCACACGTAGAATACGAAACTGATGAT-AGACA-TTATGCACATGTCGATTGTCCTGGCCACGCCGA-TTATA-TT
>GZ2VM8F07IKITU
CC-ACTTT-GACAGCTGCAAT-CACCAAATGTTT-AG-CAAGGAAAGGTCAAGCAAAA-TTCAAAGATTACGGT--GAAATTGATAAA-GCTCCCGAAGAGAAAGCGAGGGG-TAT-CACCA-T-CG-CTACGGCACACGTAGAACACGAAACTGATGAT-AGACA-TTATGCACATGTCGATTGTCCTGGCCACGCCGA-TTATA-TT
>GZ2VM8F07IDBV4
CC-ACTTT-GACAGCTGCAAT-CACCAGATGTTT-AG-CAAGGAAAGGTCAAGCAAAA-TTCAAAGATTACGGT--GAAATTGATAAA-GCTCCCGAAGAGAAAGCGAGGGG-TAT-CACCA-T-CG-CTACGGCACACGTAGAATACGAAACTGATGAT-AGACA-TTATGCACATGTCGATTGTCCTGGCCACGCCGA-TTATA-TT
>GZ2VM8F07H732E
CC-ACTTT-GACAGCTGCAAT-CACCAAATGTTT-AG-CAAGGAAAGGTCAAGCAAAA-TTCAAAGATTACGGT--GAAATTGATAAA-GCTCCCGAAGAGAAAGCGAGGGG-TAT-CACCA-T-CG-CTACAGCACACGTAGAATACGAAACTGATGAT-AGACA-TTATGCACATGCCGATTGTCCTGGCCACGCCGA-TTATA-TT
>GZ2VM8F07ID19F
CC-ACTTT-GACAGCTGCAAT-CACCAAATGTTT-AG-CAAGGAAAGGTCAAGCAAAA-TTCAAAGATTACGGT--GAAATTGATAAA-GCTCCCGAAGAGAAAGCGAGGGG-TAT-CACCA-T-CG-CTGCGGCACACGTAGAATACGAAACTGATGAT-AGACA-TTATGCACATGTCGATTGTCCTGGCCACGCCGA-TTATA-TT
>GZ2VM8F07IHWP8
CC-ACTTC-GACAGCTGCAAT-CACCAAATGTTT-AG-CAAGGAAAGGTCAAGCAAAA-TTCAAAGATTACGGT--GAAATTGATAAA-GCTCCCGAAGAGAAAGCGAGGGG-TAT-CACCA-T-CG-CTACGGCACACGTAGAATACGAAACTGATGAT-AGACA-TTATGCACATGTCGATTGTCCTGGCCACGCCGA-TTATA-TT
>GZ2VM8F07H1GXV
CC-TCTTT-GACAGCTGCAAT-CACCAAATGTTT-AG-CAAGGAAAGGTCAAGCAAAA-TTCAAAGATTACGGT--GTAATTGATAAA-GCTCCCGAAGAGAAGGCGAGGGG-TAT-

CACCA-T-CG-CTACGGCACACGTAGAATACGAAACTGATGAT-AGACA-
TTATGCACATGTCGATTGTCCTGGCCACGCCGA-TTATA-TT
>GZ2VM8F07H7W8V
CC-ACTTT-GACAGCTGCAAT-CACCAAATGTTT-TG-CAAGGAAAGGTCAAGCAAAA-
TTCAAAGATTACGGT--GAAATTGATAAA-GCTCCCGAAGAGAAAGCGAGGGG-TAT-
CACCA-T-CG-CTACGGCACACGTAGAATACGAAACTGATGAT-AGACA-
TTATGCACATGTCGATTGTCCTGGCCACGCCGA-TTATA-TT
>GZ2VM8F07IOPNA
CC-ACTTT-GACAGCTGCAATACACCAAATGTTT-AG-
CAAGGAAAGGTCAAGCAAAAGTTCAAAGATTACGGT--GAAATTGATAAA-
GCTCCCGAAGAGAAAGCGAGGGG-TAT-CACCA-T-CG-
CTACGGCACACGTAGAATACGAAACTGATGAT-AGACA-
TTATGCACATGTCGATTGTCCTGGCCACGCCGA-TTATA-TT
>GZ2VM8F07ICVMH
CC-ACTTT-GACAGCTGCAAT-CACCAAATGTTT-AGTCAAGGAAAGGTCAAGCAAAA-
TTCAAAGATTACGGT--GAAATTGATAAA-GCTCCAGAAGAGAAAGCGAGGGG-TAT-
CACCA-T-CG-CTACGGCACACGTAGAATACGAAACTGATGAT-AGACA-
TTATGCACATGTCGATTGTCCTGGCCACGCCGA-TTATA-TT
>GZ2VM8F07IJZ5L
CC-ACTTT-GACAGCTGCAAT-CACCAAATGGTTTAG-CAAGGAAAGGTCAAGCAAAA-
TTCAAAGATTACGGT--GAAATTGATAAA-GCTCCCGAAGAGAAAGCGAGGGG-TAT-
CACCA-T-CG-CTACGGCACACGTAGAATACGAAACTGATGAT-AGACA-
TTATGCACATGTCGATTGTCCTGGCCACGCCGA-TTATA-TT
>GZ2VM8F07IO5CY
CC-ACTTT-GACAGCTGCAAT-CACCAAATGTTT-AG-CAAGGAAAGGTCAAGCAAAA-
TTCAAAGATTACGGT--GAAATTGATAAA-GCTCCAGAAGAGAAAGCGAGGGGGTAT-
CACCA-T-CG-CTACGGCACACGTAGAATACGAAACTGATGAT-AGACA-
TTATGCACATGTCGATTGTCCTGGCCACGCCGA-TTATA-TT
>GZ2VM8F07IIRX8
CC-ACTTT-GACAGCTGCAAT-CACCAAATGTTT-AG-CAAGGAAAGGTCAGGCAAAA-
TTCAAAGATTACGGTTTGAAATTGATAAA-GCTCCCGAAGAGAAAGCGAGGGG-TAT-
CACCA-T-CG-CTACGGCACACGTAGAATACGAAACTGATGAT-AGACA-
TTATGCACATGTCGATTGTCCTGGCCACGCCGA-TTATA-TT
>GZ2VM8F07H6D78
CC-ACTTT-GACAGCTGCAAT-CACCAAATGTTTTAG-CAAGGAAAGGTCAAGCAAAA-
TTCAAAGATTACGGT--GAAATTGATAAA-GCTCCCGAAGAGAAAGCGAGGGG-TAT-
CACCA-T-CG-CTACGGCACACGTAGAATACGAAGCTGATGAT-AGGCA-
TTATGCACATGTCGATTGTCCTGGCCACGCCGA-TTATA-TT
>GZ2VM8F07HX41B
CC-ACTTTTGACAGCTGCAAT-CACCAAATGTTT-AG-CAAGGAAAGGTCAAGCAAAA-
TTCAAAGATTACGGT--GAAATTGATAAA-GCTCCCGAAGAGAAAGCGAGGGG-TAT-
CACCA-T-CG-CTACGGCACACGTAGAATACGAAACTGATGAT-AGACA-
TTATGCACATGTCGATTGTCCTGGCCACGCCGA-TTATA-TT
>GZ2VM8F07IG9HL
CCCACTTT-GACAGCTGCAAT-CACCAAATGTTT-AG-CAAGGAAAGGTCAAGCAAAA-
TTCAAAGATTACGGT--GAAATTGATAAA-GCTCCCGAAGAGAAAGCGAGGGG-TAT-

CACCA-T-CG-CTACGGCACACGTAGAATACGAAACTGATGAT-AGACA-
TTATGCACATGTCGATTGTCCTGGCCACGCCGA-TTATA-TT
>GZ2VM8F07HXPX3
CC-GCTTT-GACAGCTGCAAT-CACCAAATGTTT-AG-CAAGGAAAGGTCAAGCAAAA-
TTCAAAGATTACGGT--GAAATTGATAAA-GCTCCCGAAGAGAAAGCGAGGGG-TAT-
CACCA-T-CG-CTACGGCACACGTAGAATACGAAACTGATGAT-AGACA-
TTATGCACATGTCGATTGTCCTGGCCACGCCGA-TTATA-TT
>GZ2VM8F07IFLXH
CC-ACTTT-GACAGCTGCAAT-CACCAAATGTTT-AG-CAAGGAAAGGTCAAGCAAAA-
TTCAAAGATTACGGT--GAAATTGATAAA-GCTCCCGAAGAGAAAGCGAGGGG-TAT-
CACCA-T-CA-CTACGGCACACGTAGAATACGAAACTGATGAT-AGACA-
TTATGCACATGTCGATTGTCCTGGCCACGCCGA-TTATA-TT
>GZ2VM8F07IC2AF
CC-ACATT-GACAGCTGCAAT-CACCAAATGTTT-AG-CAAGGAAAGGTCAAGCAAAA-
TTCAAAGATTACGGT--GAAATTGATAAA-GCTCCCGAAGAGAAAGCGAGGGG-TAT-
CACCA-T-CG-CTACGGCACACGTAGAGTACGAAACTGATGAT-AGACA-
TTATGCACATGCCGATTGTCCTGGCCACGCCGA-TTATA-TT
>GZ2VM8F07IB1CI
CC-ACTTT-GACGGCTGCAAT-CACCAAATGTTT-AG-CAAGGAAAGGTCAAGCAAAA-
TTCAAAGATTACGGT--GAAATTGATAAA-GCTCCCGAAGAGAAAGCGAGGGG-TAT-
CACCA-T-CG-CTACGGCACACGTAGAATACGAAACTGATGAT-AGACA-
TTATGCACATGTCGATTGTCCTGGCCACGCCGA-TTATA-TT
>GZ2VM8F07H2K17
CC-ACTTT-GACAGCTGCAAT-CACCAAATGTTT-AG-CAAGGAAAGGTCAAGCAAAA-
TTCAAAGATTACGGT--GAAATTGATAAA-GCTCCCGAAGAGAAAGCGAGGGG-TAT-
CACCA-T-CG-CTACGGCACACGTAGAATACGAAACCGATGAT-AGACA-
TTATGCACATGTCGATTGTCCTGGCCACGCCGA-TTATG-TT
>GZ2VM8F07INO8K
CC-ACTTT-GACAGCTGCAAT-CACCAAATGTTT-AG-CAAGGAAAGGTCAAGCAAAA-
TTCAAAGATTACGGT--GAAATTGATAGA-GCTCCAGAAGAGAAAGCGAGGGG-TAT-
CACCA-T-CG-CTACGGCACACGTAGAATACGAAACTGATGAT-AGACA-
TTATGCACATGTCGATTGTCCTGGCCACGCCGA-TTATA-TT
>GZ2VM8F07H9QJS
CC-ACTTT-GGCAGCTGCAAT-CACCAAATGTTT-AG-CAAGGAAAGGTCAAGCAAAA-
TTCAAAGATTACGGT--GAAATTGATAAA-GCTCCAGAAGAGAAAGCGAGGGG-TAT-
CACCA-T-CG-CTACGGCACACGTAGAATACGAAACTGATGAT-AGACA-
TTATGCACATGTCGATTGTCCTGGCCACGCCGA-TTATA-TT
>GZ2VM8F07H8C8B
CC-ACTTT-GACAGCTGCAAT-CACCAAATGTTT-AG-CAAGGAAAGGTCAAGCAAAA-
TTCAAAGATTACGGC--GAAATTGATAAA-GCTCCCGAAGAGAAAGCGAGGGG-TAT-
CACCA-T-CG-CTACGGCACACGTAGAATACGAAACTGATGAT-AGACA-
TTATGCACATGTCGATTGTCCTGGCCACGCCGA-TTATA-TT
>GZ2VM8F07H25TJ
CC-ACTTT-GACAGCTGCAAT-CACCAAATGTTT-AG-CAAGGAAAGGTCAAGCAAAG-
TTCAAAGATTACGGT--GAAATTGATAAA-GCTCCAGAAGAGAAAGCGAGGGG-TAT-
CACCA-T-CG-CTACGGCACACGTAGAATACGAAACTGATGAT-AGACA-
TTATGCACATGTCGATTGTCCTGGCCACGCCGA-TTATA-TT

>GZ2VM8F07H27G6
CC-ACTTT-GACAGCTGCAAT-CACTAAATGTTT-AG-CAAGGAAAGGTCAAGCAAAA-
TTCAAAGATTACGGT--GAAATTGATAAA-GCTCCCGAAGAGAAAGCGAGGGG-TAT-
CACCA-T-CG-CTACGGCACACGTAGAATACGAAACTGATGAT-AGACA-
TTATGCACATGTCGATTGTCCTGGCCACGCCGA-TTATA-TT
>GZ2VM8F07IGFUQ
CC-ACTTT-GACAGCTGCAAT-CACCAAATGTTT-AG-CAAGGAAAGGTCAAGCAAAA-
TTCAAAGATTACGGT--GAAATTGATAAA-GCTCCCGAAGAGAAAGCGAGGGG-TAT-
CACTA-T-CG-CTACGGCACACGTAGAATACGAAACTGATGAT-AGACA-
TTATGCACATGTCGATTGTCCTGGCCACGCCGA-TTATA-TT
>GZ2VM8F07IKM7W
CC-ACTTT-GACAGCTGCAAT-CACCAAATGTTT-AG-CAAGGAAAGGTCAAGCAAAA-
TTCAAAGATTACGGT--GAAATTGATAAA-GCTCCAGAAGAGAAAGCGAGGGG-TAT-
ACACCAT-CG-CTACGGCACACGTAGAATACGAAACTGATGAT-AGACA-
TTATGCACATGTCGATTGTCCTGGCCACGCCGA-TTATA-TT
>GZ2VM8F07ICAFB
CC-ACTTT-GACAGCTGCAAT-CACCAAATGTTT-AG-CAAGGAAAGGTCAAGCAAAA-
TTCAAAGATTACGGT--GAAATTGATAAA-GCTCCAGAAGAGAAAGCGAGGGG-TAT-
CACCA-T-CG-CTACGGCACACGTAGAATACGAAACTGATGAT-AGACA-
TTATGCACATGTCGATTGTCCTGGCCACGCCGA-TTGTA-TT
>GZ2VM8F07IMZYA
CC-ACTTT-GACAGCTGCAAT-CACCAAATGTTT-AG-CAAGGAAAGGTCAAGCAAAA-
TTCAAAGATTACGGT--GAAATTGATAAA-GCTCCAGAAGAGAAAGCGAGGGG-TAT-
CACCA-T-CG-CTACGGCACACGTAGAATACGAAACTGATGAT-AGACA-
TTATGCACATGTCGATTGTCCTGTCCACGCCGA-TTATA-TT
>GZ2VM8F07IHNWI
CC-ACTTT-GACAGCTGCAAT-CACCAAATGTTT-AG-CAAGGAAAGGTCAAGCAAAA-
TTCAAAGATTACGGT--GAAATTGATAAA-GCTCCAGAAGAGAAAGCGAGGGG-TAT-
CACCA-T-CG-CTACGGCACACGTAGAATACGAAACTGATGAT-AGACA-
TTATGCACATGTCGATTGTCCTGGCCGCGCCGA-TTATA-TT
>GZ2VM8F07IF67O
CC-ACTTT-GACAGCTGCAAT-CACCAAATGTTT-AG-CAAGGAAAGGTCAAGCAAAA-
TTCAAAGATTACGGT--GAAATTGATAAA-GCTCCAGAAGAGAAAGGGAGGGG-TAT-
CACCA-T-CG-CTACGGCACACGTAGAATACGAAACTGATGAT-AGACA-
TTATGCACATGTCGATTGTCCTGGCCACGCCGA-TTATA-TT
>GZ2VM8F07IBXOE
CC-ACTTT-GACAGCTGCAAT-CACCAAATGTTT-AG-CAAGGAAAGGTCAAGCAAAA-
TTCAAAGATTACGGT--GAAATTGATAAA-GCTCCAGAAGAGAAAGCGAGGGG-TAT-
CACCA-T-CG-CTACGGCACACGTAGAATACGAAACTGATGTT-AGACA-
TTATGCACATGTCGATTGTCCTGGCCACGCCGA-TTATA-TT
>GZ2VM8F07IAHAF
CC-ACTTT-GACAGCTGCAAT-CACCAAATGTTT-AG-CAAGGAAAGGTCAAGCAAAA-
TTCAAAGATTACGGT--GAAATTGATAAA-GCTCCCGAAGAGAAAGCGAGGGG-CAT-
CACCA-T-CG-CTACGGCACACGTAGAATACGAAACTGATGAT-AGACA-
TTATGCACATGTCGACTGTCCTGGCCACGCCGA-TTATA-TT
>GZ2VM8F07H81VA

CC-ACTTT-GACAGCTGCAAT-CACCAAATGTTT-AG-CAAGGAAAGGTCAAGCAAAA-
TTCAAAGATTACGGT--GAAATTGATAAA-GCTCCCGAAGAGAAAGCGAGGGG-TAT-
CACCA-T-CG-CTACGGCACACGTAGAATACGAAACTGATGAT-AGACA-
TTATGCACATGTCGATTGTCCTGGCCACGCCAA-TTATA-TT
>GZ2VM8F07H6KW4
CC-ACTTT-GACAGCTGCAAT-CACCAAATGTTT-AG-CAAGGAAAGGTCAAGCAAAA-
TTCAAAGATTACGGT--GAAATTGATAAA-GCTCCCGAAGAGAAAGCGAGGGG-TAT-
CACCA-T-CG-CTACGCCACACGTAGAATACGAAACTGATGAT-AGACA-
TTATGCACATGTCGATTGTCCTGGCCACGCCGA-TTATA-TT
>GZ2VM8F07IQ1S8
CC-ACTTT-GACAGCTGCAAT-CACCAAATGTTT-AG-CAAGGAAAGGTCAAGCAAAA-
TTCAAAGATTACGGT--GAAATTGATAAA-GCTCCCGAAGAGAAAGCGAGGGG-TAT-
CACCA-T-CG-CTACGGCACACGTAGAATACGAAACTGATGAT-AGACA-
TTATGCACATGTCAATTGTCCTGGCCACGCCGA-TTATA-TT
>GZ2VM8F07H73UL
CC-ACTTT-GACAGCTGCAAT-CACCAAATGTTT-AG-CAAGGAAAGGTCAAGCAAAA-
TTCAAAGATTACGGT--GGAATTGATAAA-GCTCCCGAAGAGAAAGCGAGGGG-TAT-
CACCA-T-CG-CTACGGCACACGTAGAATACGAAACTGATGAT-AGACA-
TTATGCACATGTCGATTGTCCTGGCCACGCCGA-TTATA-TT
>GZ2VM8F07IHOR4
CC-ACTTT-GACAGCTGCAAT-CACCAAATGTTT-AG-CTAGGAAAGGTCAAGCAAAA-
TTCAAAGATTACGGT--GAAATTGATAAA-GCTCCAGAAGAGAAAGCGAGGGG-TAT-
CACCA-T-CG-CTACGGCACACGTAGAATACGAAACTGATGAT-AGACA-
TTATGCACATGTCGATTGTCCTGGCCACGCCGA-TTATA-TT
>GZ2VM8F07H2ND5
CC-ACTTT-GACAGCTGCAAT-CACCAAATGTTT-AG-CAAGGAAAGGTCAAGCAAAA-
TTCAAGGATTACGGT--GAAATTGATAAA-GCTCCAGAAGAGAAAGCGAGGGG-TAT-
CACCA-T-CG-CTACGGCACACGTAGAATACGAAACTGATGAT-AGACA-
TTATGCACATGTCGATTGTCCTGGCCACGCCGA-TTATA-TT
>GZ2VM8F07IDODW
CC-ACTTT-GACAGCTGCAAT-CACCAAATGTTT-AG-CAAGGAAAGGTCAAGCAAAA-
TTCAAAGATTACGGT--GAAATTGATAAA-GCTCCAGAAGAGAGAGCGAGGGG-TAT-
CACCA-T-CG-CTACGGCACACGTAGAATACGAAACTGATGAT-AGACA-
TTATGCACATGTCGATTGTCCTGGCCACGCCGA-TTATA-TT
>GZ2VM8F07HYWEA
CT-ACTTT-GACAGCTGCAAT-CACCAAATGTTT-AG-CAAGGAAAGGTCAAGCAAAA-
TTCAAAGATTACGGT--GAAATTGATAAA-GCTCCCGAAGAGAAAGCGAGGGG-TAT-
CACCA-T-CG-CTACGGCACACGTAGAATACGAAACTGATGAT-AGACA-
TTATGCACATGTCGATTGTCCTGGCCACGCCGA-TTATA-TT
>GZ2VM8F07IDIWH
CC-ACTTT-GACAGCTGCAAT-CACCAAATGTTT-AG-CAAGGAAAGGTCAAGCAAAA-
TTCAAAGATTACGGT--GAAATTGATAAA-GCTCCAGAAGAGAAAGCGAGGGG-TAT-
CACCA-T-CG-CTACGGCACACGTAGAATATGAAACTGATGAT-AGACA-
TTATGCACATGTCGATTGTCCTGGCCACGCCGA-TTATA-TT
>GZ2VM8F07IOKE3
CC-ACTTT-GACAGCTGCAAT-CACCAAATGTTT-AG-CAAGGAAAGGTCAAGCGGAA-
TTCAAAGATTACGGT--GAAATTGATAAA-GCTCCCGAAGAGAAAGCGAGGGG-TAT-

CACCA-T-CG-CTACGGCACACGTAGAATACGAAACTGATGAT-AGACA-
TTATGCACATGTCGATTGTCCTGGCCACGCCGA-TTATA-TT
>GZ2VM8F07H4WJ6
CC-ACTTT-GACAGCTGCAAT-CACCAAATGTTT-AG-CAAGGAAAGGTCAAGCAAAA-
TTCAAAGATTACGGT--GAAATTGATAAA-GCTCCCGAAGAGAAAGCGAGGGG-TAT-
CACCA-T-CG-CTACGGCACACGTAGAATACGAAACTGATGAT-AGACA-
TTATGCACATGTCGATTGTTCTGGCCACGCCGA-TTATA-TT
>GZ2VM8F07HX8GO
CC-ACTTT-GACAGCTGCAAT-CACCAAATGTTT-AG-CAAGGAAAGGTCAAGCAAAA-
TTCAAAGATTACGGT--GAAATTGACAAA-GCTCCCGAAGAGAAAGCGAGGGG-TAT-
CACCA-T-CG-CTACGGCACACGTAGAATACGAAACTGATGAT-AGACA-
TTATGCACATGTCGATTGTCCTGGCCACGCCGA-TTATA-TT
>GZ2VM8F07IL6M0
CC-ACTTT-GACAGCTGCAAA-CACCAAATGTTT-AG-CAAGGAAAGGTCAAGCAAAA-
TTCAAAGATTACGGT--GAAATTGATAAA-GCTCCAGAAGAGAAAGCGAGGGG-TAT-
CACCA-T-CG-CTACGGCACACGTAGAATACGAAACTGATGAT-AGACA-
TTATGCACATGTCGATTGTCCTGGCCACGCCGA-TTATA-TT
>GZ2VM8F07ILPEG
CC-ACTTT-GACAGCTGCAAT-CACCAAATGTTT-AG-CAAGGAAAGGTCAAGCAAAA-
TTCAAAGATTACGGT--GAAATTGATAAA-GCTCCAGGAGAGAAAGCGAGGGG-TAT-
CACCA-T-CG-CTACGGCACACGTAGAATACGAAACTGATGAT-AGACA-
TTATGCACATGTCGATTGTCCTGGCCACGCCGA-TTATA-TT
>GZ2VM8F07IM0A3
CC-ACTTT-GACAGTTGTAAT-CACCAAATGTTT-AG-CAAGGAAAGGTCAAGCAAAA-
TTCAAGGATTACGGT--GAAATTGATAAA-GCTCCCGAAGAGAAAGCGAGGGG-TAT-
CACCA-T-CG-CTACGGCACACGTAGAATACGAAACTGATGAT-AGACA-
TTATGGACATGTCGATTGTCCTGGCCACGCCGA-TTATA-TT
>GZ2VM8F07IABR9
CC-ACTTT-GACAGCTGCAAT-CACCAAATGTTT-AG-CAAGGAAAGGTCAAGCAAAA-
TTCAAAGATTACGGT--GAAATTGATAAA-GCTCCAGAAGAGAAAGCGAGGGG-TAT-
CACCA-T-CG-CCACGGCACACGTAGAATACGAAACTGATGAT-AGACA-
TTATGCACATGTCGATTGTCCTGGCCACGCCGA-TTATA-TT
>GZ2VM8F07H1VIO
CC-ACTTT-GACAGCTGCAAT-CACCAAATGTTT-AG-CAAGGAAAGGTCAAGCAAAA-
TTCAAAGATTACGGT--GAAATTGATAAA-GCTCCCGAAGAGAAAGCGAGGGG-TAT-
CACCA-T-CG-CTACGGCACACGTAGAATACGAAACTGATGAT-AGACA-
TTAGGCACATGTCGATTGTCCTGGCCACGCCGA-TTATA-TT
>GZ2VM8F07II2G5
CC-ACTTT-GACAGCTGCAAT-CACCAAATGTTT-AG-CAAGGAAAGGTCAAGCAAAA-
TTCAAAGATTACGGT--GAAATTGATAAA-GCTCCCGAAGAGAAAGCGAGGGG-TAT-
CACCAAT-CG-CTACGGCACACGTAGAATACGAAACTGATGAT-AGACA-
TTATGCACATGTCGATTGTCCTGGCCACGCCGA-TTATA-TT
>GZ2VM8F07IP9TM
CC-ACTTT-GACAGCTGCAAT-CACCAAATGTTT-AG-CAAGGAAAGGTCAAGCAAAA-
TTCAAAGATTACGGT--GAAATTGATAAA-GCTCCCGAAGAGAAAGCGAGGGG-TAT-
CACCA-T-CG-CTACGGCACACGTAGAATACGAAACTGATGAT-AGACA-
TTATGCACATGTCGATTGTCCTAGCCACGCCGA-TTATA-TT

>GZ2VM8F07IQCSU
CC-ACTTT-GACAGCTGCAAT-CACCAAATGTTT-AG-CAAGGAAAGGTCAAACAAAA-TTCAAAGATTACGGT--GAAATTGATAAA-GCTCCCGAAGAGAAAGCGAGGGG-TAT-CACCA-T-CG-CTACGGCACACGTAGAATACGAAACTGATGAT-AGACA-TTATGCACATGTCGATTGTCCTGGCCACGCCGA-TTATA-TT
>GZ2VM8F07H2BXC
CC-ACTTT-GACAGCTGCAAT-CACCAAATGTTT-AG-CAAGGAAAGGTCAAGCAAAA-TTCAAAGATTACGGT--GAGATTGATAAA-GCTCCAGAAGAGAAAGCGAGGGG-TAT-CACCA-T-CG-CTACGGCACACGTAGAATACGAAACTGATGAT-AGACA-CTATGCACATGTCGATTGTCCTGGCCACGCCGAATTATA-TT
>GZ2VM8F07H1RNV
CC-ACTTT-GACAGCTGCAAT-CACCAAATGTTT-AG-CAAGGAGAGGTCAAGCAAAA-TTCAAAGATTACGGT--GAAATTGATAAA-GCTCCCGAAGAGAAAGCGAGGGG-TAT-CACCA-T-CG-CTACGGCACACGTAGAATACGAAACTGATGAT-AGACA-TTATGCACATGTCGATTGTCCTGGCCACGCCGA-TTATA-TT
>GZ2VM8F07H8458
CC-ACTTT-GACAGCTGCAAT-CACCAAATGTTT-AG-CAAGGAAAGGTCAAGCAAAA-TTCAAAGATTACGGT--GAAATTGATAAA-GCTCCAGAAGAGAAAGCGAGGGG-TAT-CACCA-T-CG-CTACGGCACACGTAGAATACGAAACTGATGAT-AAACA-TTATGCACATGTCGATTGTCCTGGCCACGCCGA-TTATA-TT
>GZ2VM8F07IKNNG
CC-ACTTT-GACAGCTGCAAT-CACCAAATGTTT-AG-CAAGGAAAGGTCAAGCAAAA-TTCAAAGATTACGGT--GAAATTGATAAA-GCTCCAGAAGAGAAAGCGAGGGG-TATACACCA-T-CG-CTACGGCACACGTAGAATACGAAACTGATGAT-AGACA-TTATGCACATGTCGATTGTCCTGGCCACGCCGA-TTATA-TT
>GZ2VM8F07HW5AV
CC-ACTTT-GACAGCTGCAAT-CACCAAATGTTT-AG-CAAGGAAAGGTCAAGCAAAA-TTCAAAGATTACGGT--GAAATTGATAAA-GCTCCAGAAGAGAAAGCGAGGGC-TAT-CGCCA-T-CG-CTACGGCACACGTAGAATACGAAACTGATGAT-AGACA-TTATGCACATGTCGATTGTCCT-GCCACGCCGA-TTATA-TT
>GZ2VM8F07INIDL
CC-ACTTT-GACAGCTGCAAT-CACCAAATGTTT-AG-CAAGGAAAGGTCAAGCAAAA-TTCAAAGATTACGGT--GAAATTGATAAA-GTTCCCGAAGAGAAAGCGAGGGG-TAT-CACCA-T-CG-CTACGGCACACGTAGAATACGAAACTGATGAT-AGACA-TTATGCACATGTCGATTGTCCTGGCCACGCCGA-TTATA-TT
>GZ2VM8F07IPFJU
CC-ACTTT-GACAGCTGCAAT-CACCAAATGTTT-AG-CAAGGAAAGGTCAAGCAGAA-TTCAAAGATTACGGT--GAAATTGATAAA-GCTCCCGAAGAGAAAGCGAGGGG-TAT-CACCG-T-CG-CTACGGCACACGTAGAATACGAAACTGATGAT-AGACA-TTATGCACATGTCGATTGTCCTGGCCACGCCGA-TTATA-TT
>GZ2VM8F07IP4C6
CC-ACTTT-GACAGCTGCAAT-CACCAAATGTTT-AG-CAAGGAAAGGTCAAGCAAAA-TTCAAAGATTACGGT--GAAATTGATAAA-GCTCCCGAAGAGAAAGCGAGGGG-TAT-CACCA-T-CG-CTACGGCACACGTAGGATACGAAACTGATGAT-AGACA-TTATGCACATGTCGATTGTCTTGGCCACGCCGA-TTATA-TT
>GZ2VM8F07IRAXE

CC-ACTTT-GACAGCTGCAAT-CACCAAATGTTT-AG-CAAGGAAAGGTCAAGCAACA-
TTCAAAGATTACGGT--GAAATTGATAAA-GCTCCCGAAGAGAAAGCGAGGGG-TAT-
CACCA-T-CG-CTACGGCACACGTAGAATACGAAACTGATGAT-AGACA-
TTATGTACATGTCGATTGTCCTGGCCACGCCGA-TTATA-TT
>GZ2VM8F07H5R8I
CC-ACTTT-GACAGCTGCAAT-CACCAAATGTTT-AG-CAAGGAAAGGTCAAGCAAAA-
TTCAAAGATTACGGT--GAAATTGATAAA-GCTCCCGAAGAGAAAGCGAGGGG-TAT-
CACCA-T-CG-CCACGGCACACGTAGAATACGAGACTGATGAT-AGACA-
TTATGCACATGTCGATTGTCCTGGCCACGCCGA-TTATA-TC
>GZ2VM8F07HYB59
CC-ACTTT-GACAGCTGCAAT-CACCAAATGTTT-AG-CAAGGAAAGGTCAAGCAAAA-
TTCAAAGATTACGGT--GAAATTGATAAA-GCTCCAGAAGAGGAAGCGAGGGG-TAT-
CACCA-T-CG-CTACGGCACACGTAGAATACGAAACTGATGAT-AGACA-
TTATGCACATGTCGATTGTCCTGGCCACGCCGA-TTATA-TT
>GZ2VM8F07ILRGC
CC-ACTTT-GACAGCTGCAAT-CACCAAATGTTT-AG-CAAGGAAAGGTCAAGCAAAA-
TTCAAGGATTACGGT--GAAATTGATAAA-GCTCCCGAAGAGAAAGCGAGGGG-TAT-
CACCA-T-CG-CTACGGCACACGTAGAATTCGAAACTGATGAT-AGACA-
TTATGCACATGTCGATTGTCCTGGCCACGCCGA-TTATA-TT
>GZ2VM8F07H9RIZ
CC-ACTTT-GACAGCTGCAAT-CACCAAATGTTT-AG-CAAGGAAAGGTCAAGCGAAA-
TTCAAAGATTACGGT--GAAATTGATAAA-GCTCCCGAAGTGAAAGCGAGGGG-TAT-
CACCA-T-CG-CTACGGCACACGTAGAATACGAAACTGATGAT-AGACA-
TTATGCACATGTTGATTGTCCTGGCCACGCCGA-TTATA-TT
>GZ2VM8F07IC5W5
CC-ACTTT-GACAGCTGCAAT-CACCAAATGTTT-AG-CAAGGAAAGGTCAAGCAAAA-
TTCAAAGATTACGGT--GAAATTGATAAA-GCTCCCGAAGAGAAAGCGGGGGG-TAT-
CACCA-T-CG-CTACGGCACACGTAGAATACGAAACTGATGAT-AGACA-
TTATGCACATGTCGATTGTCCTGGCCACGCCGA-TTATA-TT
>GZ2VM8F07IHWPX
CC-ACTTT-GACAGCCGCAAT-CACCAAATGTTT-AG-CAAGGAAAGGTCAAGCAAAA-
TTCAAAGATTACGGT--GAAATTGATAAA-GCTCCCGAAGAGAAAGCGAGGGG-TAT-
CACCA-T-CG-CTACGGCACACGTAGAATACGAAACTGATGAT-AGACA-
TTATGCACATGTCGATTGTCCTGGCCACGCCGA-TTATA-TT
>GZ2VM8F07IIFAB
CC-ACTTT-GACAGCTGCAAT-CACCAAATGTTT-AG-CAAGGAAAGGTCAAGCAAAA-
TTCAAAGATTACGGT--GAAATTGATAAA-GCTCCCGAAGAGAAAGCGAGGGG-TAT-
CACCA-T-CG-CTACGGCACACGTAGAATACGAAACTGATGAT-AGACA-
TTATGCACATGTCGATTGTCCTGGCCACGCCGTATTATA-TT
>GZ2VM8F07HXRD6
CC-ACTTT-GACAGCTGCAAT-CACCAAATGTTT-AG-CAAGGAAAGGTCAAGCAAAA-
TTCAAAGATTACGGT--GAAATTGATAAA-GCTCCCGAAGAGAAAGCGAGGGG-TAT-
CACCA-T-CG-CTACGGCACACGTAGAATACGAAACTGATGAT-
AGACACTTATGCACATGTCGATTGTCCTGGCCACGCCGA-TTATA-TT
>GZ2VM8F07IFTO7
CC-ACTTT-GACAGCTGCGAT-CACCAAATGTTT-CG-CAAGGAAAGGTCAAGCAAAA-
TTCAAAGATTACGGT--GAAATTGATAAA-GCTCCAGAAGAGAAAGCGAGGGG-TAT-

CACCA-T-CG-CTACGGCACACGTAGAATACGAAACTGATGAT-AGACA-
TTATGCACATGTCGATTGTCCTGGCCACGCCGA-TTATA-TT
>GZ2VM8F07IFY6N
CC-ACTTT-GACAGCTGCAAT-CACCAAATGTTT-AG-CAAGGAAAGGTCAGGCAAAA-
TTCAAAGATTACGGT--GAAATTGATAAA-GCTCCCGAAGAGAAAGCGAGGGG-TAT-
CACCA-T-CG-CTACGGCACACGTAGAATACGAAACTGATGAT-AGACA-
TTATGCACATGTCGATTGTCCTGGCCACGCCGA-TTATA-TT
>GZ2VM8F07IGTLA
CC-ACTTT-GACAGCTGCAGT-CACCAAATGTTT-AG-CAAGGAAAGGTCAAGCAAAA-
TTCAAAGATTACGGT--GAAATTGATAAA-GCTCCCGAAGAGAAAGCGAGGGG-TAT-
CACCA-T-CG-CTACGGCACACGTAGAATACGAAACTGATGAT-GGACA-
TTATGCACATGTCGATTGTCCTGGCCACGCCGA-TTATA-TT
>GZ2VM8F07IMOUA
CC-ACTTT-GGCAGCTGCAAT-CACCAAATGTTT-AG-CAAGGAAAGGTCAAGCAAAA-
TTCAAAGATTACGGT--GAAATTGATAAA-GCTCCCGAAGAGAAAGCGTGGGG-TAT-
CACCA-T-CG-CTACGGCACACGTAGAATACGAAACTGATGAT-AGACA-
TTATGCACATGTCGATTGTCCTGGCCACGCCGA-TTATA-TT
>GZ2VM8F07IJBRD
CC-ACTTT-GACAGCTGCAAT-CACCAAATGTTT-AG-CAAGGAAAGGTCAAGCAAAA-
TTCAAAGATTACGGT--GAAATTGATAAA-GCTCCCGAAGAGAAAGCGAGGGG-TAT-
CACCA-T-CG-CTACGGCACACGTAGAATACGAAACTGATAAT-AGACA-
TTATGCACATGTCGATTGTCCTGGCCACGCCGA-TTATA-TT
>GZ2VM8F07IQB5Y
CC-ACCTT-GACAGCTGCAAT-CACCAAATGTTT-AG-CAAGGAAAGGTCAAGCAAAA-
TTCAAAGATTACGGT--GAAATTGATAAA-GCTCCAGAAGAGAAAGCGAGGGG-TAT-
CACCA-T-CG-CTACGGCACACGTAGAATACGAAACTGATGAT-AGACA-
TTATGCACATGTCGATTGTCCTGGCCACGCCGA-TTATA-TT
>GZ2VM8F07IIPA4
CC-ACCTT-GATAGCTGCAAT-CACCAAATGTTT-AG-CAAGGAAAGGTCAAGCAAAA-
TTCAAAGATTGCGGT--GAAATTGATAAA-GCTCCCGAAGAGAAAGCGAGGGG-TAT-
CACCA-T-CG-CTACGGCACACGTAGAATACGAAACTGATGAT-AGACA-
TTATGCACATGTCGATTGTCCTGGCCACGCCGA-TTATA-TT
>GZ2VM8F07IJ335
TC-ACTTT-GACAGCTGCAAT-CACCAAATGTTT-AG-CAAGGAAAGGTCAAGCAAAA-
TTCAAAGATTACGGT--GAAATTGATAAA-GCTCCCGAAGAGAAAGCGAGGGG-TAT-
CACCA-T-CG-CTACGGCACACGTAGAATACGAAACTGATGAT-AGACA-
TTATGCACATGTCGATTGTCCTGGCCACGCCGA-TTATA-TT
>GZ2VM8F07H0F5L
CC-ACTCT-GACAGCTGCAAT-CACCAAATGTTT-AG-CAAGGAAAGGTCAAGCAAAA-
TTCAAAGATTACGGT--GAAATTGATAAA-GCTCCCGAAGAGAAAGCGAGGGG-TAT-
CACCA-T-CG-CTACGGCACACGTAGAATACGAAACTGATGAT-AGACA-
TTATGCACATGTCGATTGTCCTGGCCACGCCGA-TTATA-TT
>GZ2VM8F07H93DT
CC-ACTTT-GACAGCTGCAAT-CACCAAATGTTT-AG-CACGGAAAGGTCAAGCAAAA-
TTCAAAGATTACGGT--GGAATTGATAAA-GCTCCCGAAGAGAAAGCGAGGGG-TAT-
CACCA-T-CG-CTACGGCACACGTAGAATACGAAACTGATGAT-AGATA-
TTATGCACATGTCGATTGTCCTGGCCACGCCGA-TTATA-TT

>GZ2VM8F07IH1L7
CC-ACTTT-GACAGCTGCAAT-CACCAAATGTTT-AG-CAAGGAAAGGTCAAGCAAAA-
TTCTAAGATTACGGT--GAAATTGATAAA-GCTCCCGAAGAGAAAGCGAGGGG-TAT-
CACCA-T-CG-CTACGGCACACGTAGAATACGAAACTGATGAT-AGACA-
TTATGCACATGTCGATTGTCCTGGCCACGCCGA-TTATA-TT
>GZ2VM8F07HY1IZ
CC-ACTTT-GACAGCTGCAAT-CACCAAATGTTT-AG-CAAGGAAAGGTCAAGCAAAA-
CTCAAAGATTACGGT--GAAATTGATAAA-GCTCCAGAAGAGAAAGCGAGGGG-TAT-
CACCA-T-CG-CTACGGCACACGTAGAATACGAAACTGATGAT-AGACA-
TTATGCACATGTCGATTGTCCTGGCCACGCCGA-TTATA-TT
>GZ2VM8F07H0XH5
CC-ACTTT-GACAGCTGTAAT-CACCAAATGTTT-AG-CAAGGAAAGGTCAAGCAAAA-
TTCAAAGATTACGGT--GAAATTGATAAA-GCTCCCGAAGAGAAAGCGAGGGG-TAT-
CACCA-T-CG-CTACGGCACACGTAGAATACGAAACTGATGAT-AGACA-
TTATGCACATGTCGATTGTCCTGGCCACGCCGA-TTATA-TT
>GZ2VM8F07H5GZ2
CC-ACTTT-GACAGCTGCAAT-CACCAAATGTTT-AG-CAAGGAAAGGTCAAGCAAAA-
TTCAAAGATTACTGT--GAAATTGATAAA-GCTCCAGAAGAGAAAGCGAGGGG-TAT-
CACCA-T-CG-CTACGGCACACGTAGAATACGAAACTGATGAT-AGACA-
TTATGCACATGTCGATTGTCCTGGCCACGCCGA-TTATA-TT
>GZ2VM8F07H98XM
CC-ACTTT-GACAGCTGCAAT-CACCAAATGTTT-AG-CAAGGAAAGGTCAAGCAAAA-
TTCAAAGATTACGGT--GAAATTGATAAA-GCCCCAGAAGAGAAAGCGAGGGG-TAT-
CACCA-T-CG-CTACGGCACACGTAGAATACGAAACTGATGAT-AGACA-
TTATGCACATGTCGATTGTCCTGGCCACGCCGA-TTATA-TT
>GZ2VM8F07IB0RA
CC-ACTTT-GACAGCTGCAAT-CACCAAATGTTT-AG-CAAGGAAAGGCCAAGCGAAA-
TTCAAAGATTACGGT--GAAATTGATAAA-GCTCCCGAAGAGAAAGCGAGGGG-TAT-
CACCA-T-CG-CTACGGCACACGTAGAATACGAAACTGATGAT-AGACA-
TTATGCACATGTCGATTGTCCTGGCCACGCCGA-TTATA-TT
>GZ2VM8F07H4HZV
CC-ACTTT-GACAGCTGCAAT-CACCAAATGTTT-AG-CAAGGAAAGGTCAAGCAAAA-
TTCAAAGATTTCGGT--GAAATTGATAAG-GCTCCCGAAGAGAAAGCGAGGGG-TAT-
CACCA-T-CG-CTACGGCACACGTAGAATACGAAACTGATGAT-AGACA-
TTATGCACATGTCGATTGTCCTGGCCACGCCGA-TTATA-TT
>GZ2VM8F07IH6AC
CC-ACTTT-GACAGCTGCAAT-CACCAAATGTTA-AG-CAAGGAAAGGTCAAGCAAAA-
TTCAAAGATTACGGT--GAAATTGATAAA-GCTCCCGAAGAGAAAGCGAGGGG-TAT-
CACCA-T-CG-CTACGGCACACGTAGAATACGAAACTGATGAT-AGACA-
TTATGCACATGTCGATTGTCCTGGCCACGCCGA-TTATA-TT
>GZ2VM8F07H5G1Z
CC-ACTTT-GACAGCTGCAAT-CACCAAATGTTT-AG-CAAGGAAAGGTCAAGCAAAA-
TTCAAAGATTACGGT--GAAATTGATAAA-GCTCCCGAAGAGAAAGCGAGGGG-TAT-
CACCA-T-TG-CTACGGCACACGTAGAATACGAAACTGATGAT-AGACA-
TTATGCACATGTCGATTGTCCTGGCCACGCCGA-TTATA-TT
>GZ2VM8F07IM9C8

CC-ACTTT-GACAGCTGCAAT-CACCAAATGTTT-AG-CAAGGAAAGGTCAAGCAAAA-
TTCAAAGATTACGGT--GAAATTGATAAA-GCTCCCGAAGAGAAAGCGAGGTG-TAT-
CACCA-T-CG-CTACGGCACACGTAGAATACGAAACTGATGAT-AGACA-
TTATGCACATGTCGATTGTCCTGGCCACGCCGA-TTATA-TT
>GZ2VM8F07H9V82
CC-ACTTT-GACAGCTGCAAT-CACCAAATGTTT-AG-CAAGGAAAGGTCAAGCAAAA-
TTCAAAGATTACGGT--GAAATTGATAAA-GCTCCCGAAGAGAAAGCGAGGGG-TAT-
CACCA-T-CG-CTACGGCACACGTAGAATACGAAACTGATGAT-AGACA-
TTATGCACATGTCGATTGTCCTGGCCACGCCGA-TCATA-TT
>GZ2VM8F07H41H8
CC-ACTTT-GACAGCTGCAAT-CACCAAATGTTT-AG-CAAGGAAAGGTCAAGCAAAA-
TTCAAAGATTACGGT--GAAATTGATAAA-GCTCCCGAAGAGAAAGCGAGGGG-TAT-
CACCA-C-CG-CTACGGCACACGTAGAATACGAAACTGATGAT-AGACA-
TTATGCACATGTCGATTGTCCTGGCCACGCCGA-TTATA-TT
>GZ2VM8F07IHT1Z
CC-ACTTT-GACAGCTGCAAT-CACCAAATGTTT-AG-CAAGGAAAGGTCAAGCAAAA-
TTCAAAGATTACGGT--GAAACTGATAAA-GCTCCCGAAGAGAAAGCGAGGGG-TAT-
CACCA-T-CG-CTACGGCACACGTAGAATACGAAACTGATGAT-AGACA-
TTATGCACATGTCGATTGTCCTGGCCACGCCGA-TTATA-TT
>GZ2VM8F07H1ALN
CC-ACTTT-GACAGCTGCAAT-CACCAAATGTTT-AG-CAAGGAAAGGCCAAGCAAAA-
TTCAAAGATTACGGT--GAAATTGATAAG-GCTCCCGAAGAGAAAGCGAGGGG-TAT-
CACCA-T-CG-CTACGGCACACGTAGAATACGAAACTGATGAT-AGACA-
TTATGCACATGTCGATTGTCCTGGCCACGCCGA-TTATA-TT
>GZ2VM8F07H0RXW
CC-ACTTT-GACAGCTGCAAT-CACCAAATGTTT-AG-CAAGGAAAGGTCAAGCAAAA-
TTCAAAGA-TACGGT--GAAATTGATAAA-GCTCCCGAAGAGAAAGCGAGGGG-TAT-
CACCA-T-CG-CTACGGCACACGTAGAATACGAAACTGATGAT-AGACA-
TTATGCACATGTCGATTGTCCTGGCCACGCCGA-TTATA-TT
>GZ2VM8F07IBVX9
CC-ACTTT-GACAGCTGCAAT-CACCAAATGTTT-AG-CAAGGAAAGGTCAAGCAAAA-
TTAAAAGATTACGGT--GAAATTGATAAA-GCTCCAGAAGAGAAAGCGAGGGG-TAT-
CACCA-T-CG-CTACGGCACACGTAGAATACGAAACTGATGAT-AGACA-
TTATGCACATGTCGATTGTCCTGGCCACGCCGA-TTATA-TT
>GZ2VM8F07H6YZV
CC-ACTTT-GACGGCTGCAAT-CACCAAATGTTT-AG-CAAGGAAAGGTCAAGCAAAA-
TTCAAAGATTACGGT--GAAATTGATAAA-GCTCCAGAAGAGAAAGCGAGGGG-TAT-
CACCA-T-CG-CTACGGCACACGTAGAATACGAAACTGATGAT-AGACA-
TTATGCACATGTCGATTGTCCTGGCCACACCGA-TTATAATT
>GZ2VM8F07IAEIM
CC-ACTTT-GACAGCTGCAAT-CACCAAATGTTT-AG-CAAGGAAAGGTCAAGCAAAA-
TTCAAAGATTACGGT--GAAATTGATAAA-GCTCCAGAAGAGAAAGCGAGGGG-TAT-
CACCA-T-CG-CTACGGCACACGTAGAATACGAATCTGATGAT-AGACA-
TTATGCACATGTCGATTGTCCTGGCCACGCCGA-TTATA-TT
>GZ2VM8F07IC1QH
CC-ACTTT-GACAGCTGCAAT-CACCAAATGTTT-AG-CAAGGAAAGGTCAAGCAAAA-
TTCAAAGATTACGGT--GAAATTGATAAAAGCTCCCGAAGAGAAAGCGAGGGG-TAT-

CACCA-T-CG-CTACGGCACACGTAGAATACGAAACTGATGAT-AGACA-
TTATGCACATGTCGATTGTCCTGGCCACGCCGA-TTATA-TT
>GZ2VM8F07HZEUJ
CC-ACTTT-GACAGCTGCAAT-CACCAAATGTTT-AG-CAAGGAAAGGTCAAGCAAAT-
TTCAAAGATTACGGT--GAAATTGATAAA-GCTCCCGAAGAGAAAGCGAGGGG-TAT-
CACCA-T-CG-CTACGGCACACGTAGAATACGAAACTGATGAT-AGACA-
TTATGCACATGTCGATTGTCCTGGCCACGCCGA-TTATA-TT
>GZ2VM8F07IKKB8
CC-ACTTT-GACAGCTGCAAT-CACCAAATGTTT-AG-CAAGGAAAGGTCAAGCAAAA-
TTCGAAGATTACGGT--GAAATTGATAAA-GCTCCAGAAGAGAAAGCGAGGGG-TAT-
CACCA-T-CG-CTACGGCACACGTAGAATACGAAACTGATGAT-AGACA-
TTATGCACATGTCGATTGTCCTGGCCACGCCGA-TTATA-TT
>GZ2VM8F07HZ8LD
CC-ACTTT-GACAGCTGCAAT-CACCAAATGTTT-AG-CAAGGAAAGGTCAAGCAAAA-
TTCAAAGATTACGGT--GAAATTGGTAAA-GCTCCAGAAGAGAAAGCGAGGGG-TAT-
CACCA-T-CG-CTACGGCACACGTAGGATACGAAACTGATGAT-AGACA-
TTATGCACATGTCGATTGTCCTGGCCACGCCGA-TTATA-TT
>GZ2VM8F07IH968
CC-ACTTT-GACAGCTGCAAT-CACCAAATGTTT-AG-CAAGGAAAGGTCAAGCAAAA-
TTCAGAGATTACGGT--GAAATTGTTAAA-GCTCCCGAAGAGAAAGCGAGGGG-TAT-
CACCA-T-CG-CTACGGCACACGTAGAATACGAAACTGATGAT-AGACA-
TTATGCACACGTCGATTGTCCTGGCCACGCCGA-CTATA-TT
>GZ2VM8F07IQLRE
CC-ACTTT-GACAGCTGCAAT-CACCAAATGTTT-AG-CAAGGAAAGGTCAAGCAAAA-
TTCAAAGATTACGGT--GAAATTGATAAA-GCTCCCGAAGAGAAAGCGAGGGG-TAT-
CACCA-T-CG-CTACGGCACACGTAGAATACGAAACTGATGAT-AGACA-
TTATGCACATGTCGATTGTCCTGGCCACGCCGA-TTATA-CT
>GZ2VM8F07ILLBG
CC-ACTTT-GACAGCTGCAAT-CACCAAATGTTT-AG-CAAGGAAAGGTCAAGCAAAA-
TTCAAAGATTACGGT--GAAATTGATAAA-GCTCCCGAAGAGAAAGCGAGGGG-TAT-
CACCA-T-CG-CTACGGCACACGCAGAATACGAAACTGATGAT-AGACA-
TTATGCACATGTCGATTGTCCTGGCCACGCCGA-TTATA-TT
>GZ2VM8F07IFM4P
CC-ACTTT-GACAGCTGCAAT-CACCAAATGTTT-AG-CAAGGAAAGGTCAAGCAAAA-
TTCAAAGATTACGGT--GAAATTGATAAA-GCTCCCGAAGAGAAAGCGAGGGG-TAT-
CACCA-T-CG-CTACGGCACACGTAGAATACGAAACTGATGAT-AGACA-
TTATACACATGTCGATTGTCCTGGCCACGCCGA-TTATA-TT
>GZ2VM8F07HYS63
CC-ACTTT-GACAGCTGCAAT-CACCAAATGTTT-AG-CAAGGAAAGGTCAAGCAAAA-
TTCAAAGATTACGGT--GAAATTGATAAA-GCTCCAGAAGAGAAAGCGAGGGG-TAT-
CACCA-T-CG-CTACGGCACACGTAGAATACGAAACTGATGAT-AGGCA-
TTATGCACATGTCGATTGTCCTGGCCACGCCGA-TTATA-TT
>GZ2VM8F07IHSLU
CC-ACTTT-GACAGCTGCAAT-CACCAAATGTTT-AG-CAAGGAAAGGTCAAGCAAAA-
TTCAAAGATTACGGT--GAAATTGATAAA-GCTCCCGAAGAGAAAGCGAGGGG-TAT-
CACCA-T-CG-CTACGGCACACGTAGAATACGAAACTGATGAG-AGACA-
TTATGCACATGTCGATTGTCCTGGCCACGCCGA-TTATA-TT

>GZ2VM8F07HX5KB
CC-ACTTT-GACAGCTGCAAT-CACCAAATGTTT-AG-CAAGGAAAGGTCAAGTAAAA-
TTCAAAGATTACGGT--GAAATTGATAAA-GCTCCCGAAGAGAAAGCGAGGGG-TAT-
CACCA-T-CG-CTACGGCACACGTAGAATACGAAACTGATGAT-AGACA-
TTATGCACATGTCGATTGTCCTGGCCACGCCGA-TTATA-TT
>GZ2VM8F07H2MSR
CC-ACTTT-GACAGCTGCAAT-CACCAAATGTTT-AG-CAAGGGAAGGTCAAGCAAGA-
TTCAAAGATTACGGT--GAAATTGATAAA-GCTCCAGAAGAGAAAGCGAGGGG-TAT-
CACCA-T-CG-CTACGGCACACGTAGAATACGAAACTGATGAT-AGACA-
TTATGCACACGTCGATTGTCCTGGCCACGCCGA-TTATA-TT
>GZ2VM8F07ICYVM
CC-ACTTT-GACAGCTGCAAT-CACCAAATGTTT-AG-CAAGGAAAGGTCAAGCAAAA-
TTCAAAGATTACGGT--GAAATTGATAAA-GCTCCAGAAGAGAAAGCGAGGGG-TAT-
CACCA-T-CG-CTACGGCACACGTAGAATACGAAACTGATGAT-AGACA-
TTATGCACACGTCGATTGTCCTGGCCACGCCGA-TTATA-TT
>GZ2VM8F07HZN1G
CC-ACTTT-GACAGCTGCAAT-CACCAAATGTTT-AG-CAAGGAAAGGTCAAGCAAAA-
TTCAAAGATTACGGT--GAAATTGATAAA-GCTCCCGAAGAGAAAGCGAGGGG-TAT-
CACCA-T-CG-CTACGGCACACGTAGAATACGAAACTGATGAT-AGACA-
TTATGCACATGTCGATTGTCCTGGCCTCGCCGA-TTATA-TT
>GZ2VM8F07IJN3F
CC-ACTTT-GACAGCTGCAAT-CACCAAATGTTT-AG-CAAGGAAAGGTCAAGCAAAA-
TTCAAAGATTACGGT--GAAATTGATAAA-GCTCCCGAAGAGAAAGCGAGGGG-TAT-
CACCA-T-CG-CTACGGCACACGTAGAATACGAAACTGATGTTTAGACA-
TTATGCACATGTCGATTGTCCTGGCCACGCCGA-TTATA-TT
>GZ2VM8F07H1705
CC-ACTTT-GACAGCTGCAAT-CACCAAATGTTT-AG-CAAGGAAAAGTCAAGCAAAA-
TTCAAAGATTACGGT--GAAATTGATAAA-GCTCCCGAAGAGAAAGCGAGGGG-TAT-
CACCA-T-CG-CTACGGCACACGTAGAATACGAAACTGATGAT-AGACA-
TTATGCACATGTCGATTGTCCTGGCCACGCCGA-TTATA-TT
>GZ2VM8F07HZTRR
CC-ACTTT-GACAGCTGCAAT-CACCAAATG-TT-AG-CAAGGAAAGGTCAAGCAAAA-
TTCAAAGATTACGGT--GAAATTGATAAA-GCTCCCGAAGAGAAAGCGAGGGG-TAT-
CACCA-T-CG-CTACGGCACACGTAGAATACGAAACTGATGAT-AGACA-
TTATGCACATGTCGATTGTCCTGGCCACGCCGA-TTATA-TT

#RLi_ABC

>GZ2VM8F07IGGQ9
GATTT-TGAAAGAATTTTTAATAGTATTTACCATTAACTTTCTTAAAC-
ATATCATTCATGGCTTCAAC
>GZ2VM8F07IHS0O
GATTT--GAGAGAATTTTTAATAGTATTTACCATTAACTTTCTTAAAC-
ATATCATTCATGGCTTCAAC
>GZ2VM8F07IMKEA

GATTT-
TGAAAGGATTTTTAATAGTATTTACCATTAACTTTCTTAAACCATATCATTCATGGCT
TCAAC
>GZ2VM8F07H5OS2
TGATTCTGAAAGAATTTTTAATAGTATTTACCATTAACTTTCTTAAAC-
ATATCATTCATGGCTTCAAC
