# Supplementary material for: Studying Genome Heterogeneity within the Arbuscular Mycorrhizal Fungal Cytoplasm
Source: Genome Biol Evol. 2015 Jan 7;7(2):505–21. doi: 10.1093/gbe/evv002 (PMC4350173; doi:10.1093/gbe/evv002)
Supplement: Supplementary Data [file supp_evv002_Suppl.figures_Tables_Oct30.pdf]

Supplementary Figures

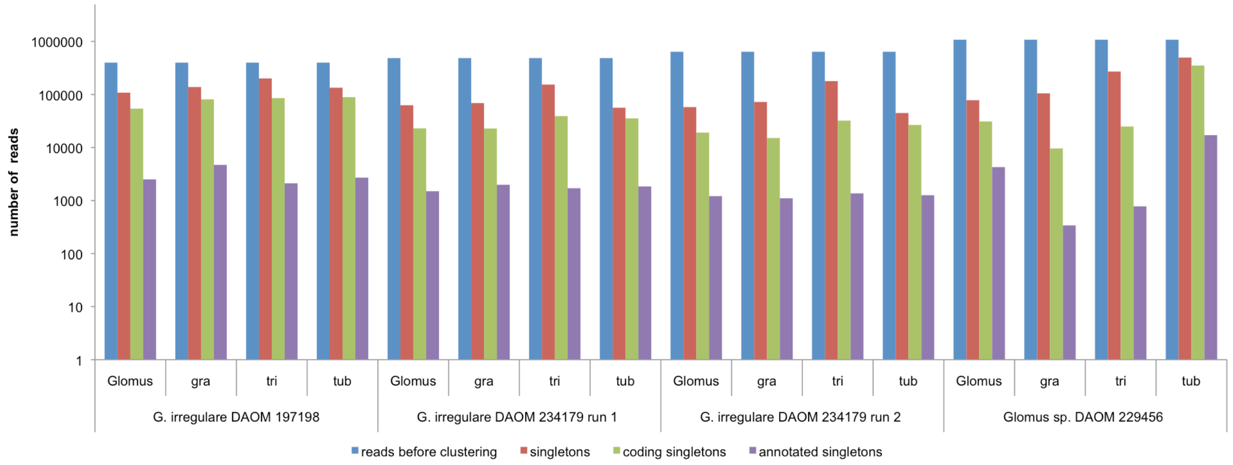

Figure S1. comparison of initial numbers of reads, numbers of singletons, coding singletons and annotated singletons between (simulated) pyrosequencing runs.

Rarefaction curves for single copy markers

a)

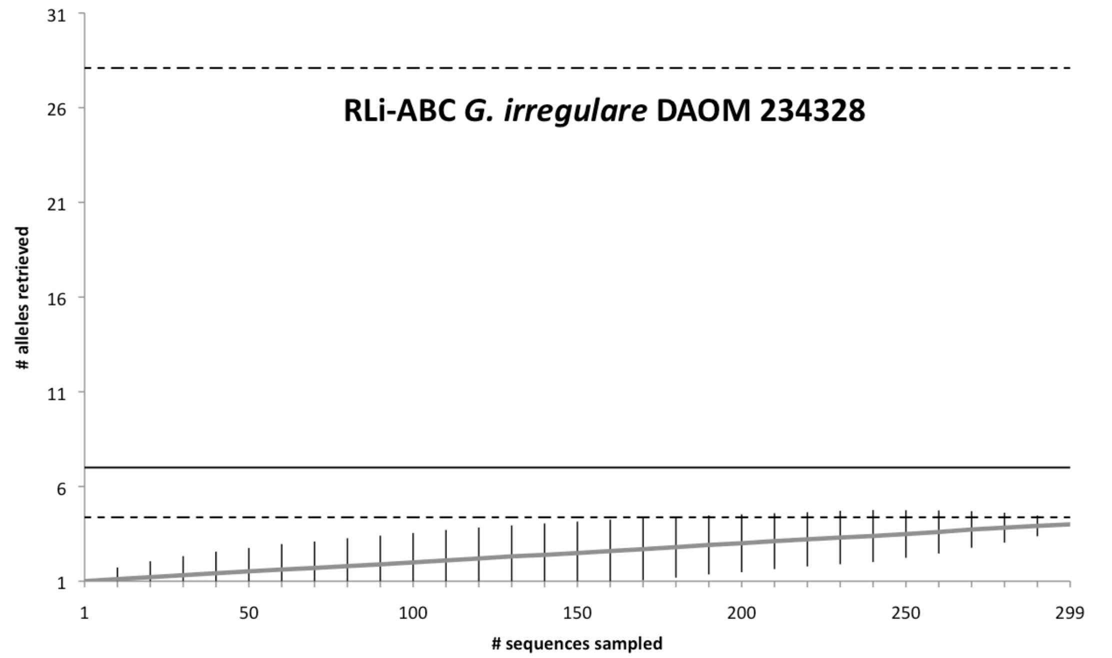

b)

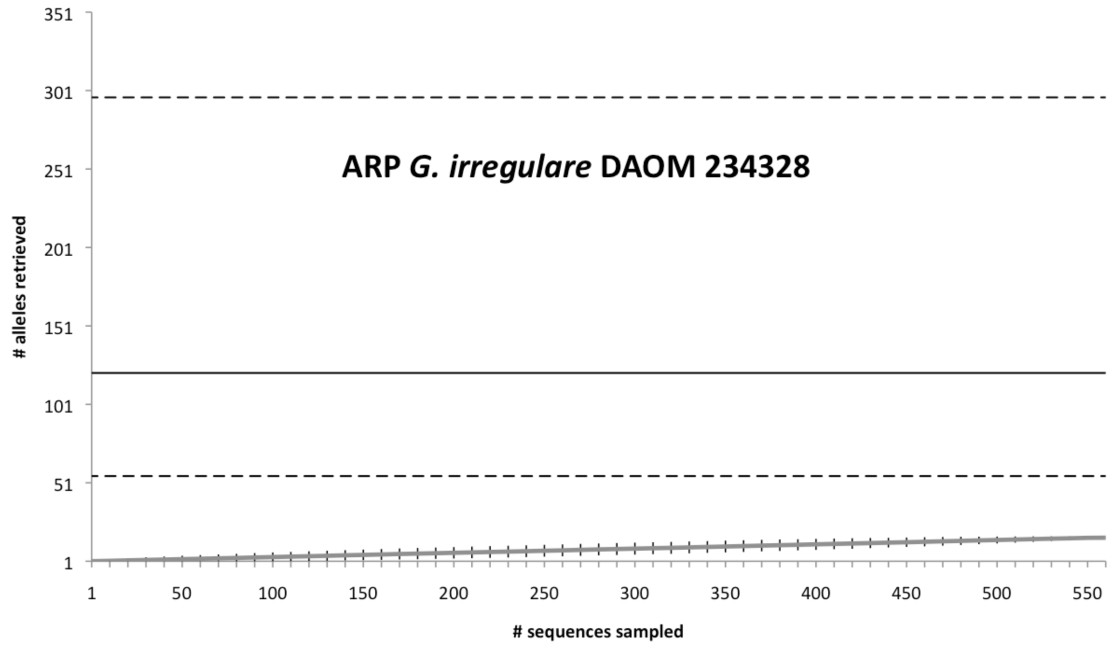

c)

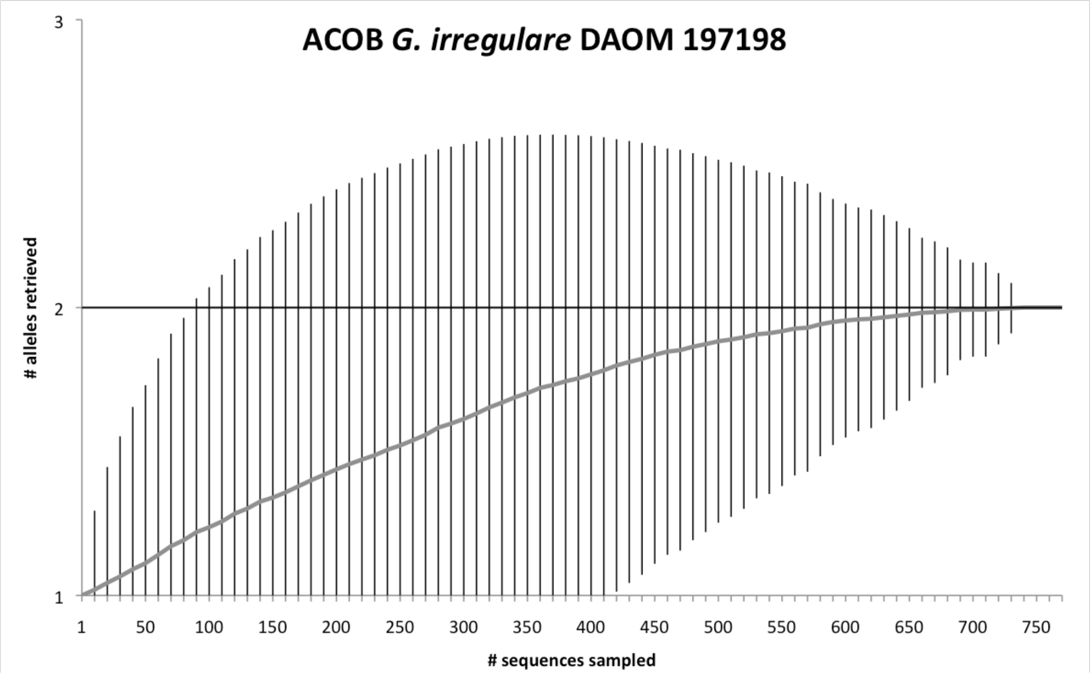

ACOB *G. irregulare* DAOM 197198
alleles retrieved
3
2
1
1
50
100
150
200
250
300
350
400
450
500
550
600
650
700
750
sequences sampled

d)

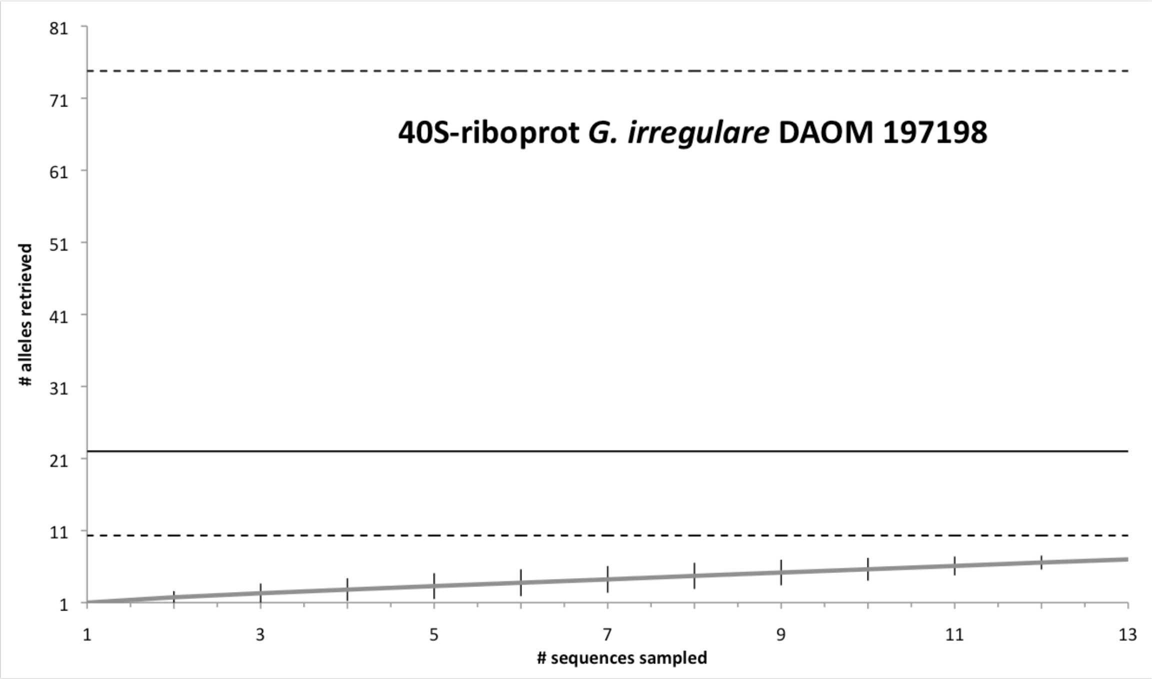

40S-riboprot *G. irregulare* DAOM 197198
alleles retrieved
81
71
61
51
41
31
21
11
1
1
3
5
7
9
11
13
sequences sampled

e)

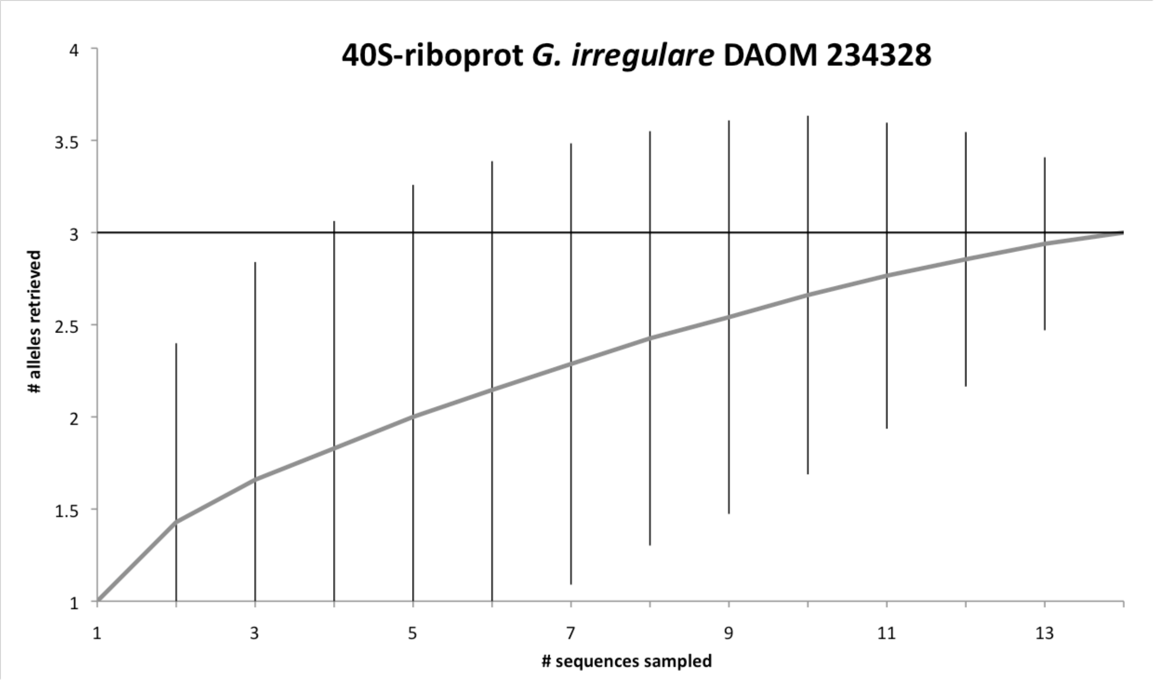
40S-riboprot *G. irregulare* DAOM 234328
alleles retrieved
sequences sampled
4
3.5
3
2.5
2
1.5
1
1
3
5
7
9
11
13

f)

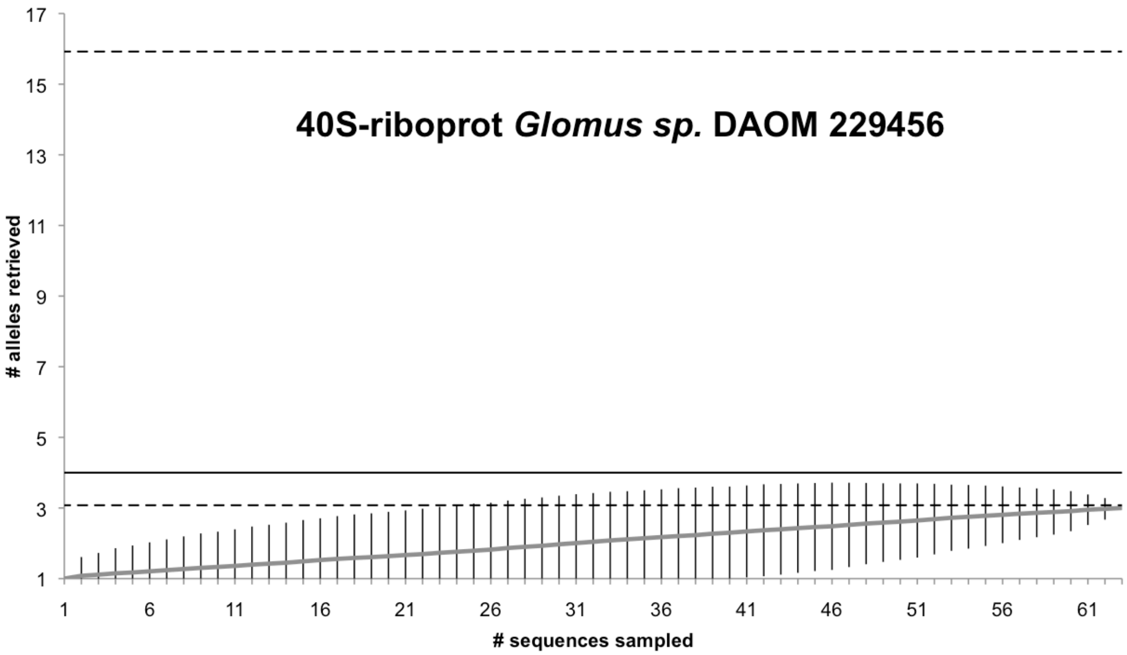
40S-riboprot *Glomus sp.* DAOM 229456
alleles retrieved
sequences sampled
17
15
13
11
9
7
5
3
1
1
6
11
16
21
26
31
36
41
46
51
56
61

g)

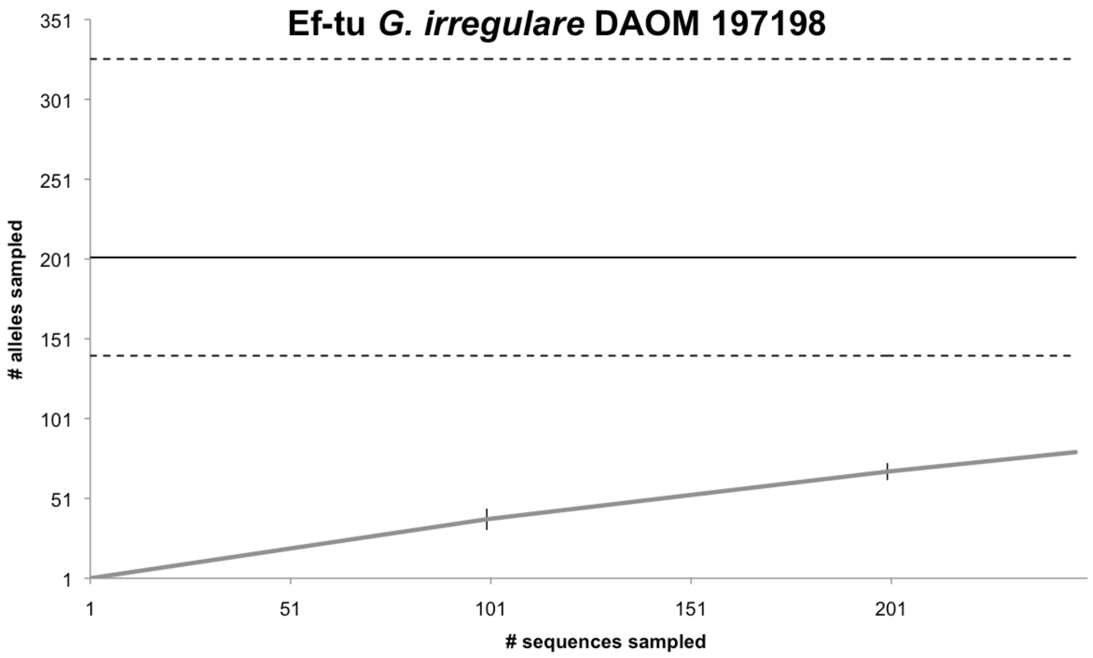
Ef-tu *G. irregulare* DAOM 197198
351
301
251
201
151
101
51
1
alleles sampled
1
51
101
151
201
sequences sampled

h)

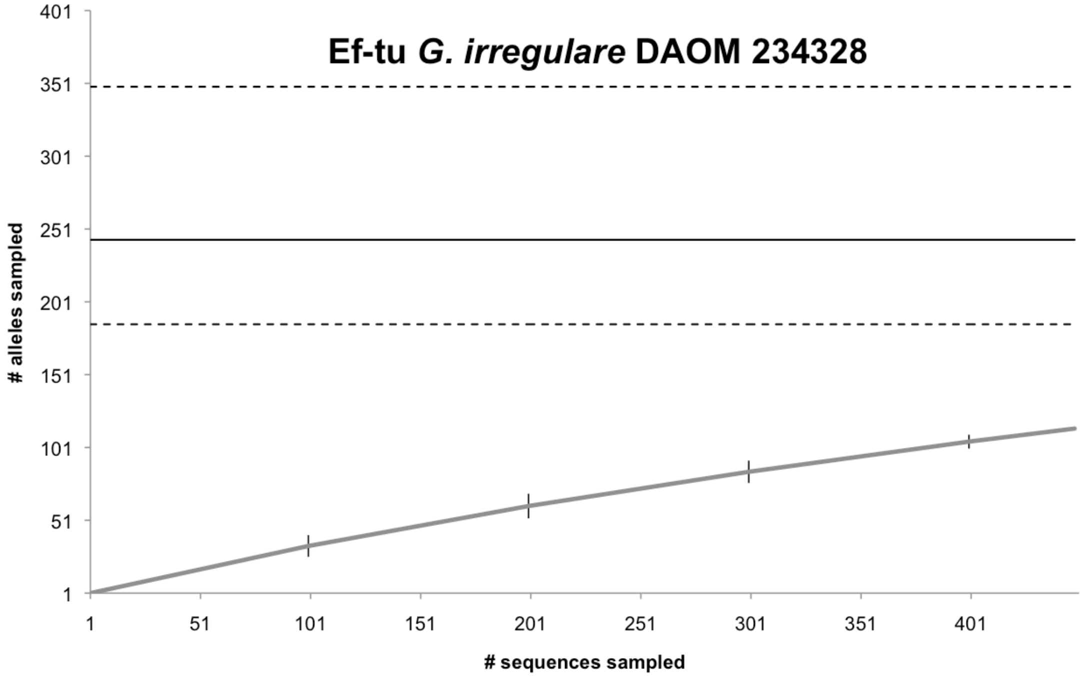
Ef-tu *G. irregulare* DAOM 234328
401
351
301
251
201
151
101
51
1
alleles sampled
1
51
101
151
201
251
301
351
401
sequences sampled

i)

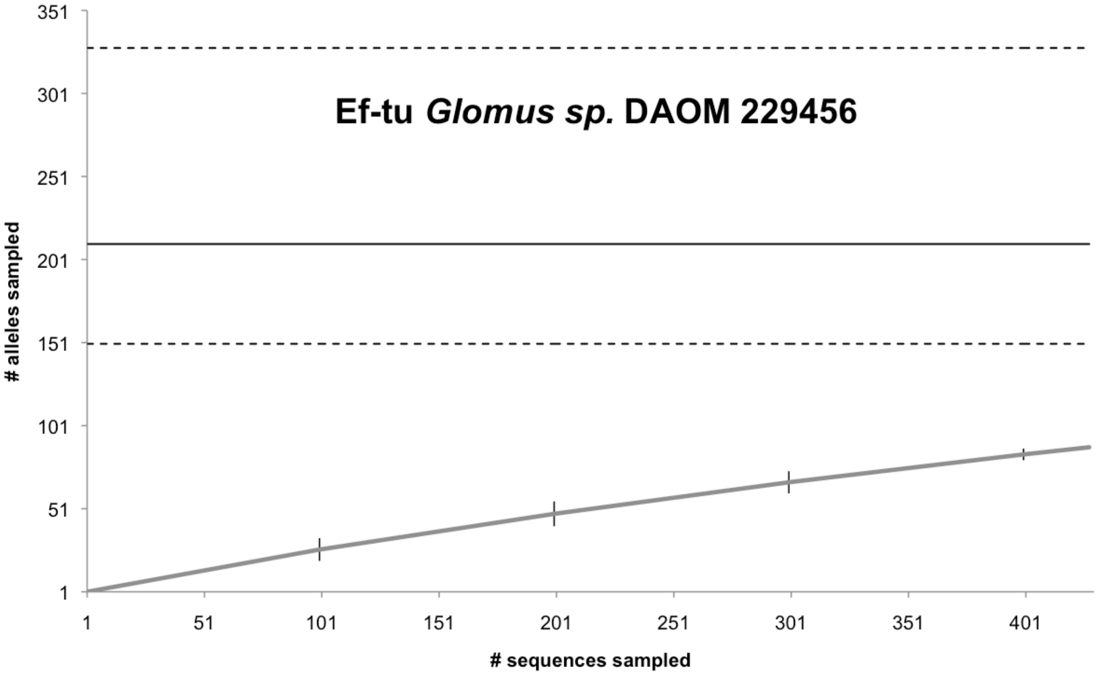

Figure S2. Rarefaction curves for single copy makers a) RLi-ABC *G. irregulare* DAOM 234328 b) 40S-riboprot *G. irregulare* DAOM 234328 c) ACOB *G. irregulare* DAOM 197198 d) ARP *G. irregulare* DAOM 197198 e) ARP *G. irregulare* DAOM 234328 f) ARP *Glomus sp* g) Ef-tu *G. irregulare* DAOM 197198 h) Ef-tu *G. irregulare* DAOM 234328 i) Ef-tu *Glomus sp* DAOM 229456. The number of recovered alleles (y axis, grey line, 95% confidence intervals indicated by vertical lines) is compared to the Chao1 value (1), which is the estimated minimum richness for each group (solid black line, 95% confidence intervals in dotted lines).

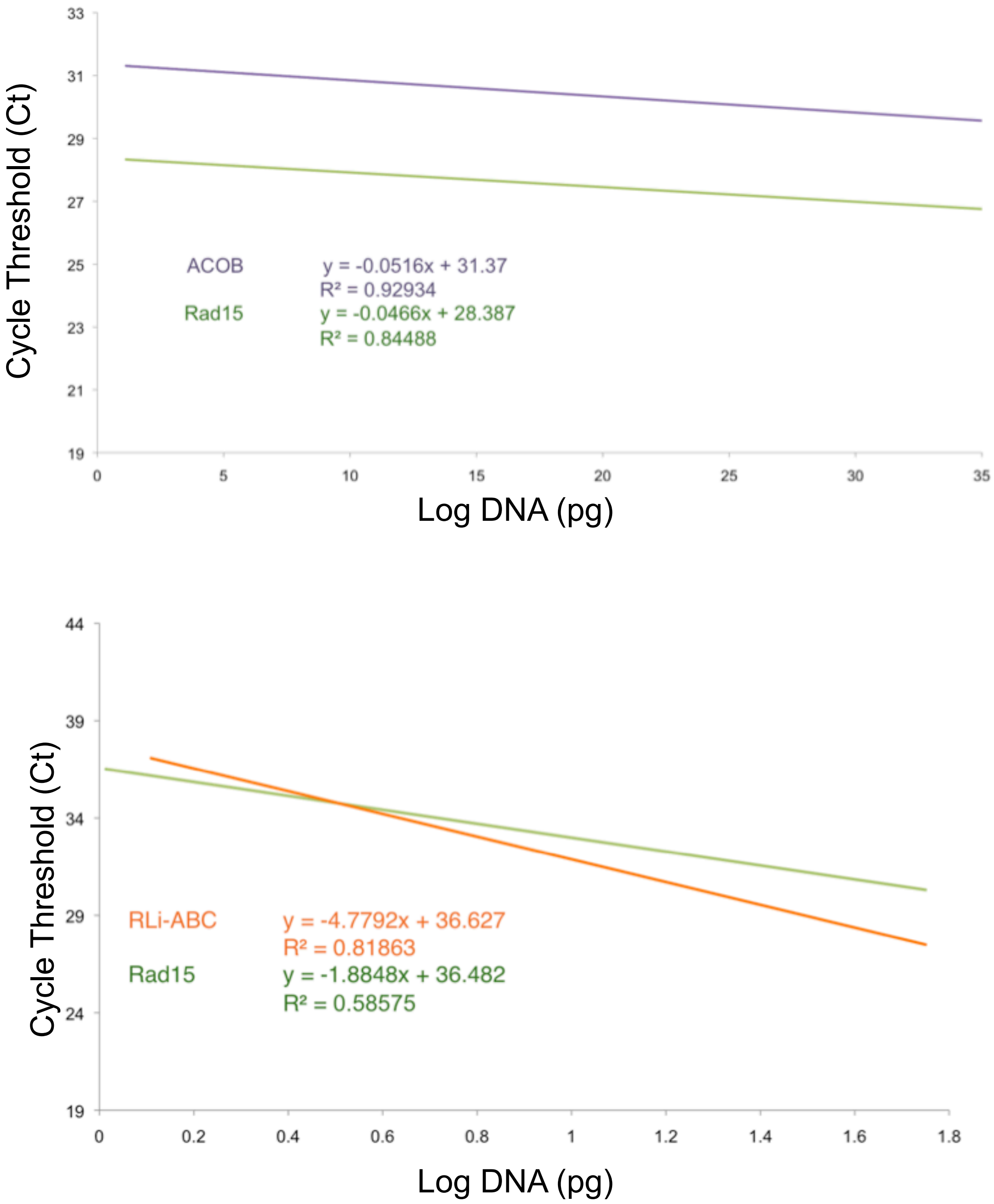

Figure S3. Results of real-time quantitative PCR for single copy markers. Linear regressions of the cycle threshold (Ct values) and the Log concentration of *R. irregularis* DAOM197198 genomic DNA (pg) that was used as template for the reaction using SYBR Green assays. Ct values of the markers ACOB and RLi-ABC were compared to Ct values of the marker Rad15.

Table S1 Genomes used for *Metasim* pyrosequencing simulations

Species	strain	genome size (Mb)	genes	repeats in genome (%)	status	coverage	source	accession no.
Candida albicans	sc5413	27.56	6,159[3]	14[4]	assembly	10.4	NCBI[3]	AACQ01000001-AACQ01000413
Candida albicans	wo1	21.68	6,107[3]	17[4]	complete	10	NCBI[3]	AAFO01000001-AAFO01000086
Candida dubliniensis	CD36	14.62	6,095	unknown	complete	11	NCBI[4]	FM992688-FM992695
Puccinia triticina	1-1 BBBD Race 1	126.64	unknown	unknown	assembly	20	PG[2]	ADAS01000001-ADAS01038776
P. graminis f. sp. *tritici*	CRL 75-36-700-3, race SCCL	88.64	21,073	36[5]	assembly	7.88	PG[2]	AAWC01000001-AAWC01004563
Tuber melanosporum	Mel28	125[6]	7,496	99[6]	assembly	10	NCBI	CABJ01000001-CABJ01004455

Information from NCBI website: http://www.ncbi.nlm.nih.gov/genomes/leuks.cgi, unless indicated otherwise

[1] *Puccinia* Group Sequencing Project, Broad Institute of Harvard and MIT (http://www.broadinstitute.org/)

[2] *Candida* Sequencing Project, Broad Institute of Harvard and MIT (http://www.broadinstitute.org/)

[3] Jackson AP, Gamble JA, Yeomans T, Moran GP, Saunders D, et al. (2009) Comparative genomics of the fungal pathogens *Candida dubliniensis* and *Candida albicans*. Genome Research 19: 2231-2244.

[4] Butler G, Rasmussen MD, Lin MF, Santos MAS, Sakthikumar S, et al. (2009) Evolution of pathogenicity and sexual reproduction in eight Candida genomes. Nature 459: 657-662.

[5] Duplessis S, Cuomo CA, Lin Y-C*, et al.* (2011) Obligate biotrophy features unraveled by the genomic analysis of rust fungi. *Proceedings of the National Academy of Sciences* **108**, 9166-9171.

[6] Martin F, Kohler A, Murat C*, et al.* (2010) Perigord black truffle genome uncovers evolutionary origins and mechanisms of symbiosis. *Nature* **464**, 1033-1038 (Table S3).

Table S2 Parameters used for *Metasim* pyrosequencing simulations

Rhizophagus spp.	strain	# reads from pyrosequencing run	mean length of reads (bp)	standard deviation of reads (bp)	# flows[1]	flow cycles[2]
R. irregularis	DAOM197198	398817	216	73	2392	598
R. irregularis (run 1)	DAOM 234179	485491	365	145	4585	1146
R. irregularis (run 2)	DAOM 234179	639222	371	142	4525	1131
Rhizophagus sp.	DAOM 229456	1078190	336	128	4083	1021

Every non-AMF genome was simulated with the parameters that had been observed for the AMF pyrosequencing runs above; *4 Rhizophagus* x 6 control genomes =24 simulations in total

[1]flows=3*(mean+(8*stddev))

[2]#cycles=(3*(mean+(8*stddev)))/4

Table S3 Primer sequence, annealing temperatures and amplicon length for five single copy markers

marker	Primer name	Sense	Test[1]	Primer sequence (5'-3')	Ta[2] (°C)	Length (bp)
RLi-ABC	NTPase_F1	F	PCR	CTGAGCGGCGGCGAACTGCAGC	49	340
	NTPase_R2	R	PCR	CAGATCATGTTCCACCAC		
ACOB	F1_2150	F	PCR	GTCAAGTGGGGTGGTGAG	48	310
	R1_2150	R	PCR	TTTGCCGCATTACTATCATCA		
	F_2150RT	F	CYBR	GCTGATATATTCTCCAGAGC		
ARP	F1_1631_gos3	F	PCR	AATGATTTTGTTAGATTATC	49	500
	R1_1631_gos3	R	PCR	GTAAAGATTACAGTGACTCTATCC		
		F	TaqMan	TGTGAATCTTGAACCTACAGCTTTG	60	98
		probe	TaqMan	ATATGACTGTAATCGATTTCGA		
		R	TaqMan	CGGAGTTGAAAGATGGTAAGCTACA		
40S-riboprot	F1_628_gos3	F	PCR	GTTCCTGTTACGAAACTTGGG	45	470
	R1_628_gos3	R	PCR	GCCAGTAACCTTGCAAGGAACAGT		
	R_628RT	R	CYBR	GCCAGTAACCTTGCAAGGAACAGT		
		F	TaqMan	AGTCAAAGAAAACAAGATCAAAACCA	59	132
		probe	TaqMan	ATATTCATTTCCCATCAAAG		
		R	TaqMan	GGCATAATCTTCATGACTTCATCTTTTA		
Ef-tu	F1_663_gos3	F	PCR	ATTGGACATGTAGATCACGGGAAA	48	310
	R1_663_gos3	R	PCR	ATGTTCACGAGTCTG		
	R_663RT	R	CYBR	CGTGTGCCGTAGCGATGGTGATA		
		F	TaqMan	CAATCACCAAATGTTTAGCAAGGA	59	151
		probe	TaqMan	AGGTCAAGCAAAATTCAAAGA		
		R	TaqMan	TGATACCCCTCGCTTTCTCTTC		
Rad15	Rad15 F	F	PCR	AGAGAGAATTATCGAATACGAGAAAATGAT	48	107
	Rad15 R	R	PCR	AGCCCATAATCTGTTTTCCCTCTT		
		F	TaqMan	TGACTAACTGTAGGGTGTAAGCAAAGG	60	200
		probe	TaqMan	CTTGAAGTTAATCCGATTCC		
		R	TaqMan	CCCAGGGTGTGGAAGAATCA		

[1]If new primers were designed for RT-PCR reactions with CYBR green fluorescent dye or TaqMan probes, the new primer is indicated in the table

[2]Annealing temperature

Table S4 Testing for differences between PID distributions

p-value\D	Glo	dub	gra	sci	tri	tub	wo
Gi1	x	0.7525	0.1683	0.7426	0.1287	0.2277	0.7525
dub	**2.20E-16**	x	0.7426	0.3267	0.7426	0.7426	0.0396
gra	0.1144	**2.20E-16**	x	0.7228	0.1386	0.1584	0.7327
sci	**2.20E-16**	**4.15E-05**	**2.20E-16**	x	0.7228	0.7228	0.2871
tri	0.3728	**2.20E-16**	0.29	**2.20E-16**	x	0.1782	0.7327
tub	0.01063	**2.20E-16**	0.1585	**2.20E-16**	0.08088	x	0.7327
wo	**2.20E-16**	1.00E+00	**2.20E-16**	**0.0004839**	**2.20E-16**	**2.20E-16**	x
Gi2	x	0.7525	0.2079	0.7426	0.1287	0.198	0.7525
dub	**2.20E-16**	x	0.7525	0.1287	0.7426	0.7426	0.0396
gra	0.0254	**2.20E-16**	x	0.7228	0.1485	0.2178	0.7327
sci	**2.20E-16**	0.3728	**2.20E-16**	x	0.7327	0.7327	0.0891
tri	0.3728	**2.20E-16**	0.2153	**2.20E-16**	x	0.1584	0.7426
tub	0.03811	**2.20E-16**	0.01659	**2.20E-16**	0.1585	x	0.7426
wo	**2.20E-16**	1.00E+00	**2.20E-16**	0.8175	**2.20E-16**	**2.20E-16**	x
Gsp	x	0.7525	0.3168	0.7525	0.1683	0.1683	0.7525
dub	**2.20E-16**	x	0.7426	0.0792	0.7525	0.7525	0.0297
gra	**7.91E-05**	**2.20E-16**	x	0.7327	0.3267	0.3168	0.7327
sci	**2.20E-16**	0.9093	**2.20E-16**	x	0.7426	0.7426	0.0693
tri	0.1144	**2.20E-16**	**4.15E-05**	**2.20E-16**	x	0.099	0.7426
tub	0.1144	**2.20E-16**	**7.91E-05**	**2.20E-16**	7.05E-01	x	0.7426
wo	**2.20E-16**	1.00	**2.20E-16**	0.9685	**2.20E-16**	**2.20E-16**	x
Gi	x	0.495	0.2475	0.4851	0.1683	0.1683	0.5149
dub	**3.56E-11**	x	0.5347	0.1188	0.5149	0.5347	0.0693
gra	0.004107	**5.79E-13**	x	0.5149	0.2574	0.1881	0.5347
sci	**9.48E-11**	0.474	**4.72E-12**	x	0.505	0.5248	0.1188
tri	0.1144	**4.72E-12**	0.002479	**1.31E-11**	x	0.1782	0.5248
tub	1.14E-01	**5.79E-13**	0.05607	**1.67E-12**	0.08088	x	0.5446
wo	**4.72E-12**	0.9685	**5.79E-13**	0.474	**1.67E-12**	**1.97E-13**	x

Statistical significance was tested Kolmogorov-Smirnov tests. D-values are listed in the upper right of the respective tables, p-values are listed in the upper left. Significant p-values are in bold font. * abbreviations for (simulated) runs*:* *R. irregularis* DAOM 234179 run1, Gi1; *Puccinia graminis,* gra; *P. triticina,* tri; *Tuber melanosporum,* tub; *Candida albicans* wo1, wo1; *C. albicans* sc5314, sc; *C. dubliniensis,* dub; *R. irregularis* DAOM 234179 run 2, Gi2; *R. irregularis* DAOM 197198, Gi; *Rhizophagus* sp. DAOM 229456, Gsp.

Table S5 Testing for differences between ORF assignment and annotation

		R. irregularis DAOM 234179 run 1						*R. irregularis* DAOM 234179 run 2								*R. irregularis* DAOM 197198								*Glomus sp.* DAOM 229456							
		gra		tri		tub		Rhizophagus		gra		tri		tub		Rhizophagus		gra		tri		tub		Rhizophagus		gra		tri		tub	
		V	p	V	p	V	p	V	p	V	p	V	p	V	p	V	p	V	p	V	p	V	p	V	p	V	p	V	p	V	p
DAOM 234179 run 1	Gi1	1	ns	0	ns	2	ns																								
	gra	na		1	ns	3	ns																								
	tri			na		5	ns																								
	tub					na																									
DAOM 234179 run 2	Gi2							na		3	ns	0	ns	3	ns																
	gra									na		0	ns	3	ns																
	tri											na		6	ns																
	tub													na																	
DAOM 197198	Gi															na		0	ns	1	ns	0	ns								
	gra																	na		1	na	3	ns								
	tri																			na		3	ns								
	tub																					na									
DAOM 229456	Gsp																							na		3	ns	3	ns	0	ns
	gra																									na		0	ns	0	ns
	tri																											na		0	ns
	tub																													na	

Statistical significance was tested with Wilcoxon Signed Rank tests in R. V and p-values are listed side by side; significant p-values are in bold font; *, $\alpha < 0.05$; **, $\alpha < 0.01$; ***, $\alpha < 0.001$; ns, not significant. Abbreviations: *Puccinia graminis*, gra; *P. triticina,* tri; *Tuber melanosporum,* tub.

Table S6 Testing for differences between KEGG annotation distributions

		R. irregularis DAOM 234179 run 1						*R. irregularis* DAOM 234179 run 2								*R. irregularis* DAOM 197198								*Rhizophagus* sp. DAOM 229456							
		gra		tri		tub		*Rhizophagus*		gra		tri		tub		*Rhizophagus*		gra		tri		tub		*Rhizophagus*		gra		tri		tub	
		D	p	D	p	D	p	D	p	D	p	D	p	D	p	D	p	D	p	D	p	D	p	D	p	D	p	D	p	D	p
DAOM 234179 run 1	Gi1	0.085	ns	0.085	ns	**0.167**	***	**0.139**	**	0.125	ns	0.093	ns	0.085	ns	**0.135**	*	**0.249**	***	**0.164**	**	**0.203**	***	**0.224**	***	**0.406**	***	0.093	ns	**0.441**	***
	gra			0.000	ns	**0.253**	***	0.089	ns	0.089	ns	**0.178**	***	**0.117**	*	**0.196**	***	**0.299**	***	**0.214**	***	**0.271**	***	**0.285**	***	**0.320**	***	**0.146**	**	**0.466**	***
	tri					**0.253**	***	0.089	ns	0.089	ns	**0.178**	***	**0.117**	*	**0.196**	***	**0.299**	***	**0.214**	***	**0.271**	***	**0.285**	***	**0.320**	***	**0.146**	**	**0.466**	***
	tub							**0.295**	***	**0.235**	***	0.110	ns	**0.196**	***	0.057	ns	**0.121**	*	0.057	ns	0.064	ns	0.107	ns	**0.573**	***	**0.153**	**	**0.327**	***
DAOM 234179 run 2	Gi2									0.082	ns	**0.224**	***	**0.206**	***	**0.274**	***	**0.388**	***	**0.303**	***	**0.342**	***	**0.342**	***	**0.399**	***	**0.160**	**	**0.555**	***
	gra											**0.160**	**	**0.135**	*	**0.192**	***	**0.306**	***	**0.221**	***	**0.260**	***	**0.278**	***	**0.338**	***	**0.128**	*	**0.498**	***
	tri													**0.121**	*	0.100	ns	**0.231**	***	0.089	ns	**0.153**	*	**0.203**	***	**0.498**	***	0.064	ns	**0.416**	***
	tub															**0.139**	**	**0.206**	***	**0.139**	**	**0.214**	***	**0.228**	***	**0.384**	***	0.089	ns	**0.370**	***
DAOM 197198	Gi																	**0.139**	**	0.032	ns	0.082	ns	0.110	ns	**0.516**	***	**0.135**	*	**0.320**	***
	gra																			**0.157**	**	0.078	ns	0.046	ns	**0.584**	***	**0.274**	***	**0.224**	***
	tri																					0.078	ns	**0.128**	*	**0.516**	***	**0.142**	**	**0.331**	***
	tub																							0.050	ns	**0.591**	***	**0.196**	***	**0.271**	***
DAOM 229456	Gsp																									**0.605**	***	**0.246**	***	**0.231**	***
	gra																											**0.466**	***	**0.733**	***
	tri																													**0.448**	***
	tub																														

Statistical significance was tested Kolmogorov-Smirnov tests. D and p-values are listed side by side; significant p-values are in bold font; *, α < 0.05; **, α < 0.01; ***, α < 0.001. Abbreviations: *Puccinia graminis*, gra; *P. triticina,* tri; *Tuber melanosporum,* tub.

Table S7 comparison of repeat structure for *Rhizophagus* and control genomes

Species name	GC level	bases masked	Total interspersed repeats	Simple repeats	Low complexity
Candida albicans wo1	33.47	5.55	3.24	1.63	0.73
Candida albicans sc5314	33.87	64.01	63.22	0.59	0.32
Candida dubliniensis	33.25	7.95	4.20	2.75	1.04
Puccinia graminis[1]	43.35	34.74	35.41	0.5	0.05
Puccinia triticina	46.34	44.78	46.38[1]	0.24	0.03
Tuber melanosporum[1]	44.86	57.30	57.51[1]	0.37	0.10
Rhizophagus irregularis DAOM 234179 run 1	28.44	58.84	55.65	0.7	4.1
Rhizophagus irregularis DAOM 234179 run 2	28.51	63.5	0.66	3.75	-
Rhizophagus sp. DAOM 229456	28.37	64.69	0.63	3.78	-

[1] confirms previously reported values, for *T. melanosporum* (Martin *et al.* 2010) and *P. graminis* (Feschotte *et al.* 2009)

Table S8 effect of different clustering and denoising approaches for allele count single copy markers

Marker	alleles			
	Preclustering difference[1]		SeqNoise	AmpliconNoise
	1 bp	2 bp		
RLi-ABC	3	1	20	15
ARP	15	2	61	30
ACOB	2	1	17	285
40S-riboprot	8	3	11	30
Ef-tu	103	2	47	111

[1]Only alleles that occur more than once are counted

Table S9 Psi-Blast results for single copy markers

marker	psi-Blast result
RLi-ABC	RNAse L inhibitor-type ATP binding cassette protein
ARP	actin-related protein (ARP) 2/3 complex subunit 2
ACOB	Inositol hexakisphosphate and diphosphoinositol-pentakisphosphate kinase
40S-riboprot	40S ribosomal protein S2
Ef-tu	Nuclear gene encoding a mitochondrial translation elongation factor Tu,

Closest match after 3 psi-BLAST iterations, database search in April 2013

Table S10 Polymorphism in the Sanger-sequenced clones from the TaqMan RT-PCR assay

Marker Name	n[1]	position[3]	alleles[2]	polymorphism
40S-riboprot	16	50-120	13	ATTTCCCATCAAAGAATTTCAAATCGTAGATCGCTTAATCAGCACTACATTAAAAGATGAAGTCAT-GAAGA
			1	........A...-.....
			1	.................A..-.....
			1	....T.....
Ef-tu	19	35-70	2	CAAAATTCAAAGATTACGGTGAAATTGTTAAAGCTCC
			16	............................A........
			1	.....C......................A........
ARP	23	1-60	14	----GAATCTTGAACCTACAGCTTTGGATATGACT--AGTCGATTTCGATGGTGTAGCTT
			5	TTGT...............................GT..............T........
			1	TTGT...............................GT...G..........N........
			1	--TT......................TG.------------------------.......
			1	TTGT...........................C...GT..............T........
			1	TTGT......................A---------------------------------
			1	

[1] Number of clones sampled
[2] sequence variants found in clones
[3] position of variable segment shown in basepairs along the allele

References

1. **Chao A, Chazdon RL, Colwell RK, Shen T-J.** 2005. A new statistical approach for assessing similarity of species composition with incidence and abundance data. Ecology Letters **8:**148-159.
2. **Martin F, Kohler A, Murat C, Balestrini R, Coutinho PM, Jaillon O, Montanini B, Morin E, Noel B, Percudani R, Porcel B, Rubini A, Amicucci A, Amselem J, Anthouard V, Arcioni S, Artiguenave F, Aury J-M, Ballario P, Bolchi A, Brenna A, Brun A, Buee M, Cantarel B, Chevalier G, Couloux A, Da Silva C, Denoeud F, Duplessis S, Ghignone S, Hilselberger B, Iotti M, Marcais B, Mello A, Miranda M, Pacioni G, Quesneville H, Riccioni C, Ruotolo R, Splivallo R, Stocchi V, Tisserant E, Viscomi AR, Zambonelli A, Zampieri E, Henrissat B, Lebrun M-H, Paolocci F, Bonfante P, Ottonello S, Wincker P.** 2010. Perigord black truffle genome uncovers evolutionary origins and mechanisms of symbiosis. Nature **advance online publication**.
3. **Martin F, Kohler A, Murat C, Balestrini R, Coutinho PM, Jaillon O, Montanini B, Morin E, Noel B, Percudani R, Porcel B, Rubini A, Amicucci A, Amselem J, Anthouard V, Arcioni S, Artiguenave F, Aury J-M, Ballario P, Bolchi A, Brenna A, Brun A, Buee M, Cantarel B, Chevalier G, Couloux A, Da Silva C, Denoeud F, Duplessis S, Ghignone S, Hilselberger B, Iotti M, Marcais B, Mello A, Miranda M, Pacioni G, Quesneville H, Riccioni C, Ruotolo R, Splivallo R, Stocchi V, Tisserant E, Viscomi AR, Zambonelli A, Zampieri E, Henrissat B, Lebrun M-H, Paolocci F, Bonfante P, Ottonello S, Wincker P.** 2010. Perigord black truffle genome uncovers evolutionary origins and mechanisms of symbiosis. Nature **464:**1033-1038.
4. **Feschotte Cd, Keswani U, Ranganathan N, Guibotsy ML, Levine D.** 2009. Exploring Repetitive DNA Landscapes Using REPCLASS, a Tool That Automates the Classification of Transposable Elements in Eukaryotic Genomes. Genome Biology and Evolution **1:**205-220.
